# Manganese Oxides Incubated in Acidic Forest Soil Retain More Organic Carbon Than Iron Oxides

**DOI:** 10.1021/acs.est.6c07327

**Published:** 2026-09-01

**Authors:** Kristen Bidas, Hui Li, Fernanda Santos, Erin C. Rooney, Benjamin Reinhart, Lilin He, Changwoo Do, Qian Zhao, Ryan Tappero, Elizabeth Herndon

**Affiliations:** † Environmental Sciences Division, Oak Ridge National Laboratory, Oak Ridge, Tennessee 37831, United States; ‡ Department of Earth and Planetary Sciences, University of Tennessee, Knoxville, Tennessee 37996, United States; § Department of Crop and Soil Sciences, 6798 North Carolina State University, Raleigh, North Carolina 27695, United States; ∥ USDA-NRCS-National Soil Survey Center, Lincoln, Nebraska 68508, United States; ⊥ X-ray Science Division, Argonne National Laboratory, Lemont, Illinois 60439, United States; # Neutron Scattering Division, 6146 Oak Ridge National Laboratory, Oak Ridge, Tennessee 37831, United States; ∇ Environmental Molecular Sciences Laboratory, Pacific Northwest National Laboratory, Richland, Washington 99354, United States; ○ National Synchrotron Light Source II, Brookhaven National Laboratory, Upton, New York 11973, United States

**Keywords:** manganese oxides, iron oxides, organic matter, carbon sequestration, organic matter degradation, oxidation, soils, transition metals

## Abstract

Iron (oxyhydr)­oxides are well-recognized contributors to soil carbon (C) storage, but the effects of manganese oxides on carbon storage and transformation are relatively unexplored. Here, the relative capacities of Fe and Mn oxides to bind and stabilize soil organic C were directly compared using an in situ incubation experiment. Quartz sands coated with either poorly crystalline Mn­(III/IV) oxides or Fe­(III) oxides, or left uncoated, were buried in a temperate forest soil for up to one year. Chemical extractions, X-ray absorption and photon spectroscopies, scanning electron microscopy, and neutron scattering were used to evaluate organic and mineral associations and transformations during the incubation. At the end of the 1-year study, Mn oxide stored ∼11× more carbon (42.0 ± 14.3 mg C/m^2^ oxide) than Fe oxide (3.72 ± 0.37 mg C/m^2^ oxide) per unit surface area. Manganese oxidation state changed over time, likely reflecting reactions between organic C and Mn oxide, while Fe oxidation state remained unaltered. Total organic C that was associated with Mn oxide was relatively enriched in carboxylic and O-alkyl-C compared to Fe oxides and quartz, either due to preferential sorption or oxidation on the mineral surface. Mn oxides also sorbed high amounts of Ca despite being incubated in an acidic, base-cation depleted soil, which potentially facilitated C sorption. Our study demonstrates that Mn oxides, with the assistance of Ca-bridging, have a greater potential to stabilize organic C than Fe oxides in acidic environments despite higher reactivity.

## Graphical abstract

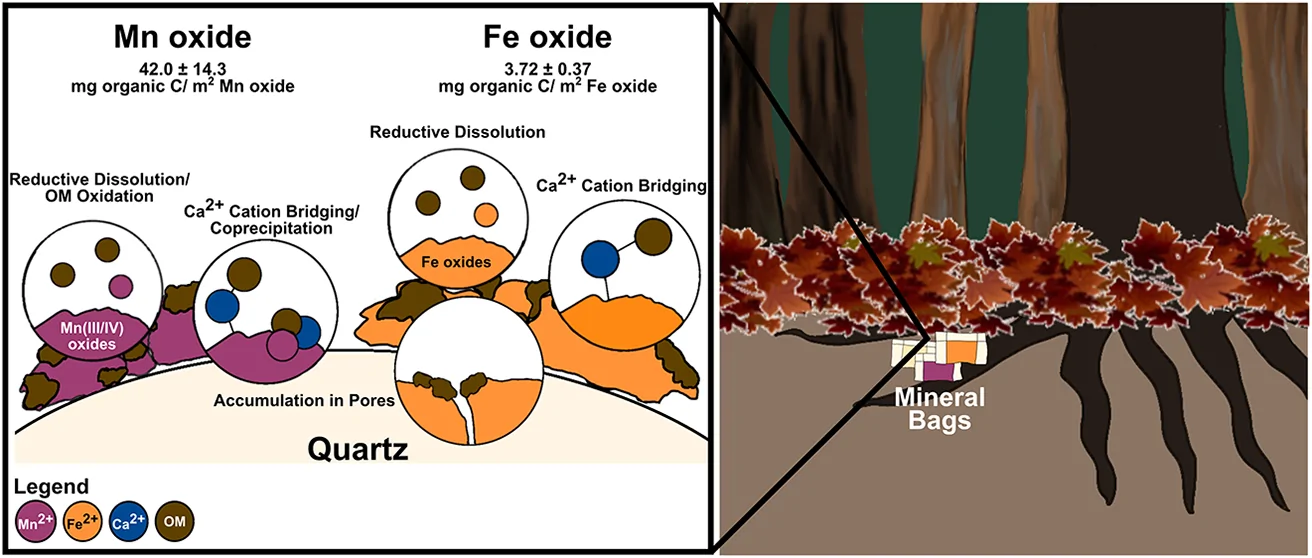

## Introduction

Soil is one of the largest terrestrial carbon (C) reservoirs and contains 3500–4800 Pg C, which is 4–6 times greater than C in the atmosphere and ∼8 times greater than C stored in live vegetation.^1,2^ The organic matter in soils can be sequestered in soil macro- and microaggregates and by interactions with minerals.^3,4^ Extensive research has been done on the ability of different clay minerals and secondary oxides, such as aluminum (Al) oxides and iron (Fe) oxides, to sequester C in soils.^5–11^ An estimated 600 Pg C is associated with these reactive minerals, particularly in forested biomes with high precipitation.^7^ Mineral association at least temporarily^12^ stabilizes organic matter against degradation; for example, organic matter associated with the Fe-oxide mineral ferrihydrite is less susceptible to degradation by microorganisms and has a longer residence time than dissolved organic matter in forest soil.^13^

Extensive literature has established that interactions between Mn oxides and individual organic compounds result in diverse reactions that can sequester or oxidize organic C (OC).^14–20^ However, only a few studies have examined how Mn oxides interact with natural organic matter, and fewer have examined these interactions in soil environments. Manganese can accumulate in surface soils via plant uptake and subsequent leaf litterfall^21–25^ as well as atmospheric inputs such as industrial pollution.^26^ Manganese (oxyhydr)­oxides (hereafter referred to as Mn oxides) are generally less abundant than Fe oxides in soils^27^ but are similarly redox-sensitive and are strong natural oxidants.^28,29^ These oxides are produced in soils through the weathering of primary minerals to release Mn­(II) and subsequent microbial oxidation of Mn­(II) to Mn­(III/IV) oxides.^30^ Soluble Mn­(III) complexes produced through Mn oxide dissolution or biotic Mn^2+^ oxidation are themselves potent intermediates that oxidize and depolymerize complex organic molecules prior to forming Mn oxides.^24,31^

Manganese oxides have high adsorption capacity but also high oxidation potential, meaning that organic matter that adsorbs to the mineral surface is also susceptible to oxidation.^30^ Adsorption of organic matter (OM) and ions to Mn oxides is possible via multiple different mechanisms, including ligand exchange surface complexation,^32^ hydrophobic interactions,^33^ electrostatic interactions,^34,35^ cation bridging,^33,36,37^ the polymerization of organic molecules,^38,39^ and physical trapping of organic molecules by Mn oxides.^40^ Manganese oxide associations with OM have been found in environments where Mn oxides accumulate, including brackish waters,^41^ cave deposits,^41^ and ferromanganese nodules in soils and sediments.^42–44^ However, the amount of C that adsorbs to Mn oxides and is stabilized versus oxidized to CO_2_ or into smaller molecules is not well understood, particularly in soil environments where the effects of Mn are often obscured within the complex soil matrix. In particular, the effects of Mn oxides on C storage are often difficult to decouple from Fe oxides, which are more abundant, co-occur with Mn, and are extracted via similar methods.

Several studies have examined how natural OM interacts with Fe oxides, particularly through coprecipitation and adsorption reactions.^32,45–50^ Short-range-ordered Fe oxides have been shown to adsorb OC because of their high surface areas, while ligand binding and coprecipitation^51,52^ mechanisms sequester OC when ferrihydrite and dissolved organic matter from forest soils are coprecipitated. The most common OC functional groups associated with Fe oxides are aromatic and carboxylic.^32^ Although several wastewater treatment studies have examined contaminant sorption to Fe oxide-coated and Mn oxide-coated sands,^53–58^ no studies have directly compared how Mn oxides and Fe oxides interact with OM in soils.

The goal of this study was to evaluate the capacity for Mn oxides to sequester OC relative to Fe oxides in soils. We hypothesized that net accumulation of OM would be greater for Fe oxides than for Mn oxides, as a portion of the adsorbed OM would react with but not be retained on the Mn oxide surfaces. To test this hypothesis, we compared the quantity and composition of OM that was associated with Mn oxides and Fe oxides using an *in situ* mineral bag incubation. Several studies have conducted incubations of mineral bags containing material such as mineral soil,^59,60^ vermiculite,^61^ and Fe oxide-coated sand,^62–64^ but no studies have investigated Mn oxides. This approach enables a direct comparison of how Fe and Mn oxides interact with and sequester natural organic matter in the soils.

## Methods

### Field Site

Walker Branch Watershed (WBW) (97.5 ha) is a temperate forested watershed located on the Oak Ridge Reservation in Oak Ridge, Tennessee. The watershed is located in the humid southern Appalachian region, with a mean annual rainfall of 134 cm and a mean annual temperature of 14.4 °C.^65^ This watershed was crop and pasture land until the land came under the management of the Atomic Energy Commission in 1941–1942.^66^ The hydrogeology,^67–70^ soil chemistry,^66,71,72^ and nutrient cycling^73–76^ at WBW have been reported previously. WBW is underlain by the Knox Formation: a siliceous, dolomitic limestone that is Late Cambrian to Early Ordovician in age.^72^ Soils in the watershed are mainly Ultisols with some Inceptisols present near the streams.^72^ The dominant secondary minerals in these soils are kaolinite, mica, and chloritized vermiculite.^77^ This experiment was conducted in a flat, upland area near the watershed ridge where soils are well-drained. The soil properties are reported in the Supporting Information.

### Mineral Incubation

Poorly crystalline Mn and Fe oxides were synthesized using methods for hydrous Mn oxide^78,79^ and ferrihydrite,^80^ respectively, then coated onto quartz (Qtz) sand (Lane Mountain Company, Valley, WA) by mixing the Qtz with HMO or ferrihydrite suspensions for 2 h, sieving to <100 μm, rinsing with deionized water to remove small particles, then drying for 24 h at 60 °C. The synthesized Mn oxide powder was previously characterized as hydrous Mn oxide^17^; however, because we could not definitively identify the mineral structure using XRD following their sorption to the quartz sand, we refer to the minerals as poorly crystalline Mn oxide and Fe oxide. While many laboratory studies use pure, synthesized Mn oxides, coating the oxides onto a substrate such as sand is more representative of how Mn oxides are found in the environment, e.g., coatings and varnishes,^81^ and limits particle movement out of the mesh bag during incubation. Although metal oxide coatings are more representative of the natural environment, the quartz does present challenges. For example, techniques like FTIR and XRD are dominated by the Qtz signal, limiting characterization of the small quantities of coated oxide.

Approximately 10 g of dry Mn oxide-coated sand, Fe oxide-coated sand, or uncoated quartz sand were sealed in 5 μm nylon mesh (Elko Filtering Co.) bags. One set of mineral bags was incubated at the soil surface under the litter layer (depth = 0 cm) for up to 365 days (7, 14, 28, 90, 180, and 365 days; *n* = 5 per treatment per time point) starting on August 13, 2020. Another set was incubated for 90 days at three depths corresponding to the O (0 cm), A (10 cm), and B (30 cm) soil horizons (3 per treatment per depth). After excavation, mineral bags were frozen at −20 °C and then freeze-dried prior to further analyses. Three soil cores were also collected in the upland soils in 0–10, 10–20, and 20–30 cm depth increments using a hand auger in August 2020. Five leaf litter replicates were collected in August 2020. The leaf litter consisted of mixed plant species and was dried and ground prior to spectroscopic analysis. Both soils and leaf litter were air-dried prior to analyses. See Supporting Methods for more information.

### Mineral Extractions and Analyses

Initial and incubated minerals were removed from the mesh bags and subsampled for characterization. To simulate interaction with rainwater, minerals were rinsed by mixing 1 g of material with 20 mL of 0.01 M KCl^82^ and shaken by hand for 30 s to obtain water-extractable OC (WEOC) and water-extractable cations. Pellets were then shaken with 25 mL of 0.11 M Na dithionite buffered with 0.11 M NaHCO_3_ for 1 h to extract reducible Fe and Mn,^83^ which are both effectively extracted by Na dithionite.^84–86^ Additional method details are provided in Supporting Information.

Total C (hereafter termed total OC (TOC)) and nitrogen (N) concentrations were analyzed using an Elementar Vario TOC cube (Hanau, Germany). To enable comparison between the minerals that were present in different concentrations, total C concentrations were normalized to dithionite-extractable Fe and Mn (molar ratios) and to the effective specific surface area of the Fe and Mn oxides. Brunauer–Emmett–Teller (BET) specific surface areas (SSAs) for Qtz, Mn oxide-coated sand, and Fe oxide-coated sand were determined using N_2_ adsorption–desorption isotherms measured with a Micromeritics Gemini VII 2390a surface area analyzer (Georgia). The effective specific surface areas (ESSA) for Mn oxide and Fe oxide, i.e., the differences in surface areas between the coated quartz and uncoated quartz normalized to the mass of each oxide, were calculated to estimate the surface area (m^2^ g oxide^–1^) available for sorption once oxides were coated onto the quartz sand. The ESSA calculation represents the surface area of Mn or Fe oxides and excludes the quartz sand.

Dissolved OC in the WEOC extracts, method blanks, and analytical blanks was measured using a Shimadzu TOC-L (Shimadzu Corp., Japan). Major elements (Fe, Mn, Ca, Mg, Al, P, Si) in the water and dithionite extracts were analyzed using a ThermoFisher Scientific iCAP 7400 ICP-OES instrument at Oak Ridge National Laboratory and with ICP-OES at the University of Georgia Extension Agricultural and Environmental Services Laboratories.

Carbon K-edge near-edge X-ray absorption fine structure (NEXAFS) spectra were collected for the minerals and leaf litter at the Spherical Grating Monochromator (SGM) beamline at the Canadian Light Source. Scans were collected between 270 and 320 at 0.10 eV resolution, normalized to boron nitride, and energy calibrated. NEXAFS spectra were processed (merged, baseline corrected, and normalized) using the Athena software (0.9.26).^87^ Relative proportions of C functional groups were determined by Gaussian fits to major peaks.^88^

Bulk Mn and Fe K-edge XANES were collected by using unfocused beams at beamline 6-BM of the National Synchrotron Light Source II (NSLS-II) and at beamline 12-BM of the Advanced Photon Source (APS). Spectra were collected between −200 to +642 eV around the Mn K-edge (6 539 eV) and −200 to +855 eV around the Fe K-edge (7 112 eV). The beam was calibrated to a Mn metal foil at 6 539 eV and Fe metal foil at 7 112 eV. Fe and Mn average oxidation states were determined from linear combination fits using single-valence reference standards.^89^ Postprocessing and fitting details are provided in Supporting Information.

Calcium K-edge XANES was acquired with an unfocused beam at the XFM beamline (4-BM) at the National Synchrotron Light Source II (NSLS-II). Calcium XANES were collected in fluorescence mode over an energy range of 4003–4132 eV using 0.25 eV steps over the edge. Additional information about the beamline and data processing is provided in the Supporting Information.

X-ray photon spectroscopy was used to evaluate the surface (∼10 nm) composition of the Qtz, Mn oxide- and Fe oxide-coated sands. The samples were measured by a Physical Electronics Quantera Scanning X-ray Microprobe (Physical Electronics, Germany), which uses a focused monochromatic Al Kα (1486.7 eV) source for excitation. The X-ray beam is incident to normal to the sample, and the photoelectron detector is 45° off normal. Binding energies were determined with an accuracy of ± 0.2 eV and calibrated using the C 1s peak at 284.8 eV. High-energy resolution spectra were collected for quantifying elemental composition and determining oxidation states of metal oxides using a pass energy of 69 eV with a step size of 0.125 eV. Additional details are available in the Supporting Methods.

X-ray microprobe analysis was completed on beamline 2–3 at the Stanford Synchrotron Radiation Lightsource (SSRL). Qtz, Mn oxide- and Fe oxide-coated sand were mounted to Kapton tape. The beamline uses a Si 111 ϕ = 90 monochromator and has a focused beam size of 5 μm. Fluorescence maps were taken for Fe, Mn, Al, Si, Ca, and Mg with a 5 × 5 μm spot size. XRF spectra and correlations between elements were analyzed in Sam’s MicroAnalysis Toolkit (SMAK).^90^

Small-Angle Neutron Scattering (SANS) is a technique that is sensitive to the high hydrogen (H) concentrations in OM and can be used to detect the accumulation of OM. SANS was performed at the extended Q-range small-angle neutron scattering diffractometer (EQ-SANS BL 6) at the Spallation Neutron Source at Oak Ridge National Laboratory. EQ-SANS has a Q range of 0.002 to 1.4 Å^–1^ and a detector resolution of 5.5 × 4.3 mm. Two sample-to-detector distances of 4 and 2.5 m were used. The Qtz, Mn oxide- and Fe oxide-coated sands were packed into titanium sample holders with quartz windows and a path length of 1 mm. Sample reduction was done using DRTSANS,^91^ and spectra were graphed in Igor Pro (WaveMetrics, Lake Oswego, OR).

### Statistics

Statistics were calculated using Origin Pro (OriginPro, version 2023b. OriginLab Corporation, Northampton, MA). Significant differences between time and treatment (Qtz, Mn oxide- or Fe oxide-coated sand) or treatment with depth were calculated using two-way ANOVA followed by a Tukey ad-hoc test with a 95% confidence level.

## Results

### Mineral Characteristics

Dithionite-extractable Fe and Mn, representing Fe and Mn in reducible metal oxides, did not significantly change from 0 to 365 d for the Fe oxide-coated sand (+314 ± 242 mg Fe kg^–1^; *n* = 3) or the Mn oxide-coated sand (−58 ± 95 Mn mg kg^–1^; *n* = 3) incubated under the litter layer (*p* > 0.05). Manganese and Fe were below detection for the uncoated Qtz in the dithionite extractions and SEM/EDS (Figure S2, Tables S5, S7), indicating that reducible oxides were not present on the sand before coating and did not accumulate over time. The Mn oxide and Fe oxide coatings were amorphous in appearance in the initial material and remained amorphous after the incubation (Figure S1).

Water-extractable Mn in the Mn oxide treatment increased by 200% in the first 7 days of incubation and then sharply decreased as the incubation progressed. Water-extractable Ca increased significantly for all three treatments over time (F = 14.5, *p* < 0.0001) and was higher in the Mn oxide and Fe oxide treatments than the Qtz (F = 59.1, *p* < 0.0001). Mn oxide-coated sand had the greatest increase in Ca, which peaked at ∼23× the initial concentration by day 28 (Figure 1). Water-extractable Ca decreased with soil depth (F = 20.8, *p* < 0.0001; Figure S5) and differed between treatments (F = 13.9, *p* < 0.0004); after 90 days, the Mn oxide-coated sand incubated in surface soils had the highest concentration of water-extractable Ca (25.7 ± 2.98 mg/kg). Water-extractable Mg was also detected and showed similar patterns to Ca but did not significantly change with time or treatment (Figure 1), although the interaction between factors was significant (F = 4.20, *p* < 0.002). Water-extractable Si was only detected in single replicates of Fe oxide-coated sand (Table S7). No water-soluble Al, P, Si, Fe, or Mn was detected in the uncoated sands, reflecting the relative purity of the initial quartz and negligible adsorption of the cations to Qtz (Table S7).

**Figure 1:**
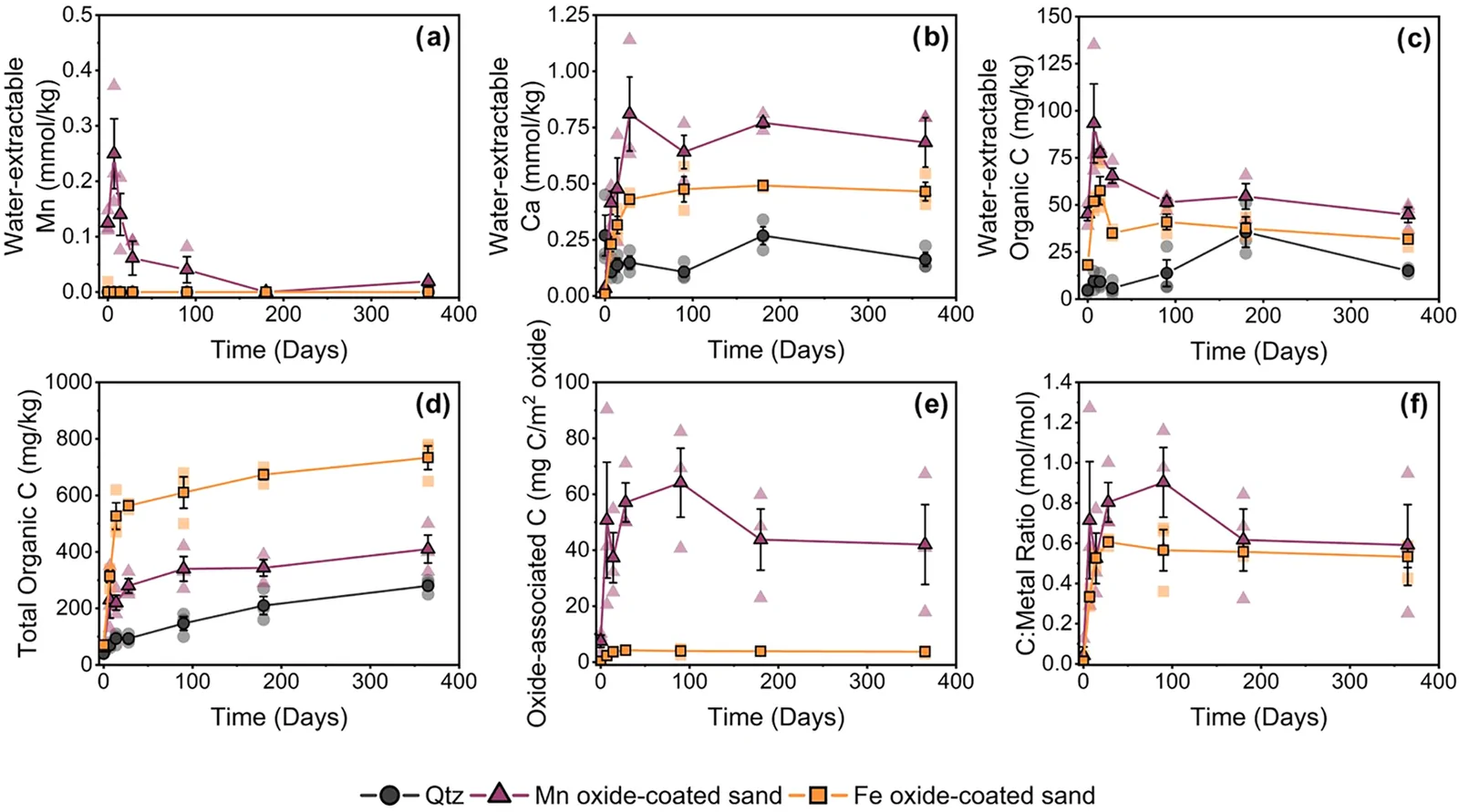
Concentrations of water-extractable (a–c) or total (d–f) elements in uncoated sand (Qtz), Mn oxide-coated sand, and Fe oxide-coated sand incubated in surface soils for up to 365 days. Water-extractable Mn (a) (mmol kg^–1^) increased for Mn oxide-coated sand. Water-extractable Ca (b) and OC (0.01 M KCl extract) (c) increased for all treatments but were highest for Mn oxide treatments. Total OC concentrations (mg C kg^–1^) were highest for Fe oxide-coated sands (d); however, total OC normalized to oxide surface area (mg C m^2^ oxide) (e) or to the metal concentration (mol C mol^–1^ metal) (f) was greatest for the Mn oxide-coated sand. Error bars represent the standard error of the mean for three replicates.

Synchrotron microprobe analysis showed that Ca was significantly correlated with Mn (*R* ^2^ = 0.855; F = 38 630, *p* < 0.0001) on the Mn oxide-coated sand (Figures 2, S6) and present as sorbed and/or organically complexed ions rather than carbonate or other mineral phases (Figure 2). No correlations between Fe and Ca were observed for Fe oxide-coated sand (Figure S9); however, the Fe oxide accumulated small quantities of Mn that were strongly correlated with Ca (*R* ^2^ = 0.822; F = 22 062, *p* < 0.0001; Figure S7). SEM/EDS analysis also showed a greater density of Ca associated with Mn and C than was observed for Fe (Figures 2, S8, Table S6).

**Figure 2:**
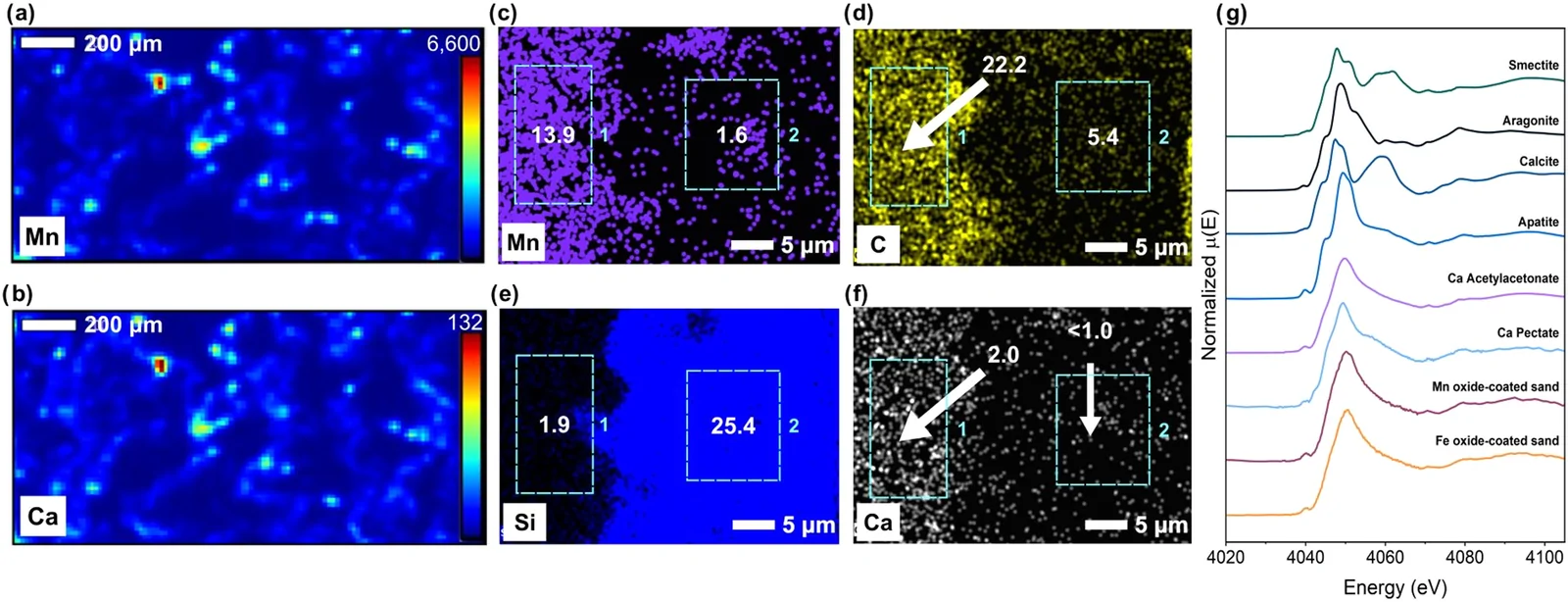
Mn oxides accumulated Ca during incubation in forest soil. Synchrotron X-ray microprobe fluorescence maps of Mn and Ca (a, b) and SEM EDS maps of Mn, C, Si and Ca (c–f) on Mn oxide-coated sand incubated for one year. The fluorescence intensities for panels (a) and (b) are shown on the right. The atomic % of each element for the SEM EDS maps is shown in the box on the map. (g) Ca XANES spectra obtained from the Mn-oxide and Fe-oxide-coated sands were consistent with reference spectra for organic-bound Ca (e.g., Ca acetylacetonate and Ca pectate) but not Ca-bearing minerals (e.g., smectite, aragonite, calcite, and apatite).

The average oxidation state (AOS) of Mn in the Mn oxide-coated sand increased slightly over time from 2.96 to 3.25, but this change was not significant (Figure 3). The percentage of Mn­(III) species decreased slightly, as evident by the decreasing shoulder peak present in the XANES spectrum (Figure 3). The decrease in Mn­(III) was concurrent with slight decreases in Mn­(II) and increases in Mn­(IV), although these changes were also not considered significant given the low number of replicates. Mn AOS on the Mn oxide surface (∼10 nm depth), determined using XPS analysis, decreased slightly from 3.26 to 3.10 by day 365 (Table S9), and the percentage of Mn­(IV) decreased from 26.2 to 10.2% while the percentage of Mn­(III) increased from 73.8 to 89.8% (Table S9). The AOS of Fe remained between 2.90 and 2.95 over time (Table S10). The Fe oxide surface was slightly more reduced, with 35 to 41% (±0.7%) Fe­(II) content and AOS = 2.59 ± 0.007 (Table S10).

**Figure 3:**
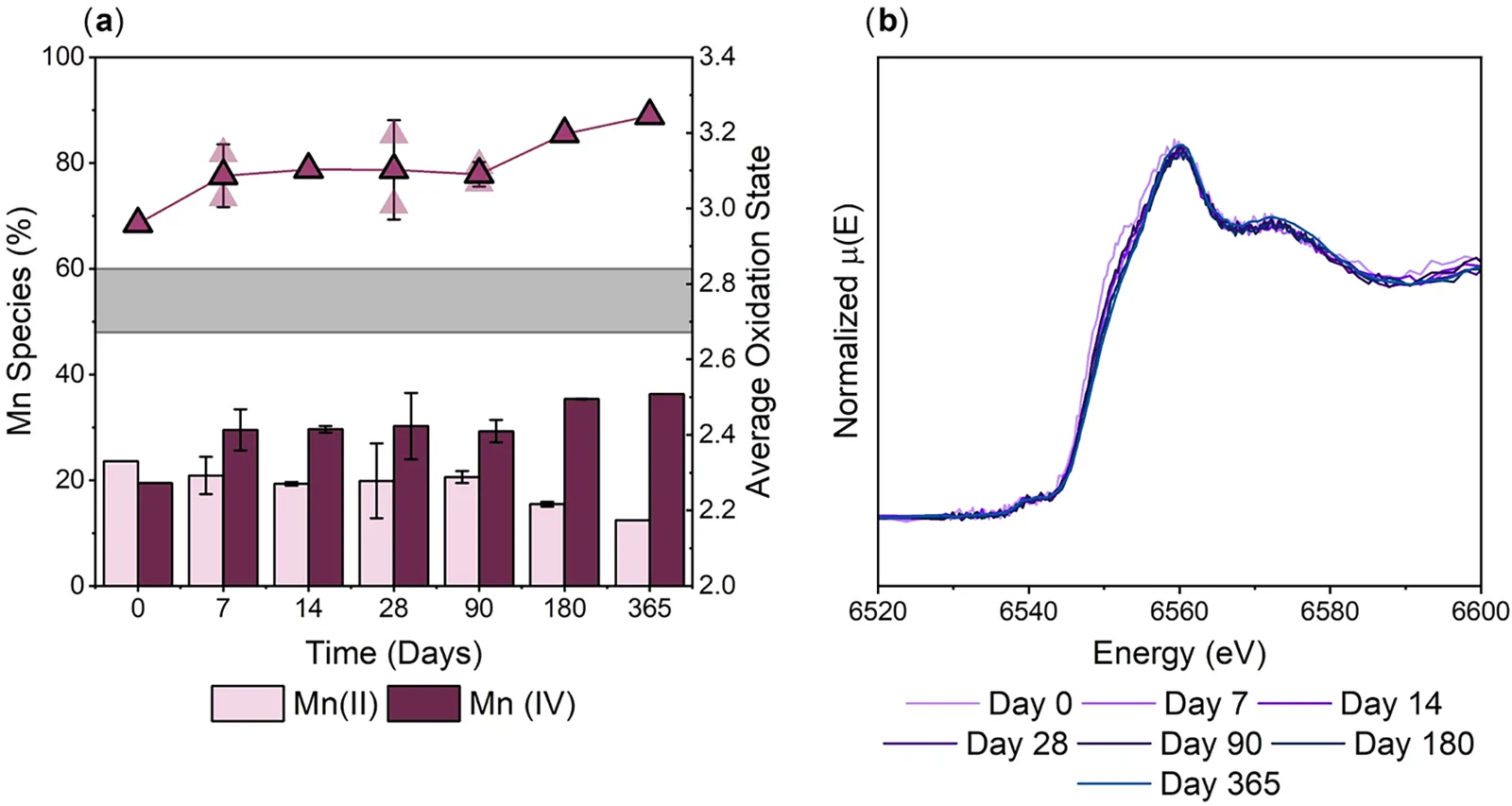
Increasing Mn oxidation state indicates Mn­(II) loss and Mn­(IV) oxide formation. The dark purple bars represent Mn­(IV) and light pink bars represent Mn­(II) (a). The gray band represents the range of Mn­(III) species, which did not change over time. Average oxidation states (AOS) of Mn increased over time in the Mn oxide-coated sand (triangles). Error bars represent the standard error of the mean; *n* = 2 except for day 0 (initial material) and day 365 (*n* = 1). Mn spectra shifted to higher energies gradually over time, indicating an increase in oxidation state (b).

### Organic Matter Accumulation and Characterization

Total OC increased over time and was higher in the Fe oxide-coated sand (increased from 70 mg kg^–1^ to 733 ± 42 mg kg^–1^) than the Mn oxide-coated sand (from 60 ± 10 mg kg^–1^ to 410 ± 49 mg kg^–1^) and uncoated sand (from 40 mg kg^–1^ to 280 ± 15 mg kg^–1^) after 365 days (Figure 1). However, higher total OC in the Fe oxide-coated sands could be attributed to higher concentrations of Fe oxides compared to Mn oxides in the initial material, i.e., 3629 ± 204 mg Fe kg-sand^–1^ versus 1017 ± 39 mg Mn kg-sand^–1^. Mn oxide accumulated more total OC over time than Fe oxide when normalized to mineral surface area (i.e., ESSA; F = 75.6, *p* < 0.0001) and per atom of metal (i.e., C/metal ratios) (Figure 1). Organic C associated with Mn oxides peaked on day 90 (64.1 ± 12.3 mg m^–2^ ESSA), while OC associated with Fe oxides plateaued by day 28 (4.23 ± 0.08 mg m^–2^ (ESSA)). Organic C normalized to SSA values reported in the literature (Figure S9, Table S4) had the same patterns as the ESSA-normalized OC (Figures 1, S9). Similarly, molar ratios of organic C:Mn (OC:Mn) peaked on day 90 (0.903 ± 0.173) and were greater than peak OC:Fe values (0.606 ± 0.011) (F = 5.09, *p* < 0.05) (Figure 1). Normalized OC concentrations were approximately ∼3–5× higher in mineral bags incubated at the soil surface compared to those incubated at 30 cm depth for both the Mn oxide- and Fe oxide-coated sands (F = 14.4, *p* < 0.0007) (Figure S10). As with the surface-incubated minerals, Mn oxide had significantly more associated OC than Fe oxide with increasing depth after normalization to surface area (F = 13.7, *p* < 0.0008, Figure S10). However, molar ratios were not significantly different between the Mn oxide- and Fe oxide-coated sand (F = 2.48, *p* = 0.142) in deeper soils (Figure S10).

Water-extractable OC (0.01 M KCl extract) was highest in the Mn oxide-coated sand and peaked on day 7 (93.3 ± 21.0 mg kg; 40.6% of TOC) before decreasing (Figure 1). Water-extractable OC for the Mn oxide-coated sand was significantly correlated with water-extractable Mn (F = 34.4, *p* < 0.0001) (Figure S11). The highest concentrations of WEOC in Fe oxide-coated sand (57.6 ± 7.4 mg kg^–1^; 10.9% of TOC) and uncoated sand (35.6 ± 8.1 mg kg^–1^; 16.9% of TOC) were comparatively low (Figure 1). Similar to TOC, WEOC was highest in mineral bags incubated under the litter layer and decreased with depth (F = 6.78, *p* < 0.007; Figure S10).

Total OC that was present in the Qtz, Mn oxide- and Fe oxide-coated sand treatments after one year was dominated by aliphatic-C followed by O-alkyl-C and carboxylic-C, as determined with NEXAFS spectroscopy (Figures S12 and S13). The relative proportions of quinone-C, phenolic-C and aromatic-C were roughly equal across all treatments. To isolate the composition of OC associated with Fe and Mn oxides, the concentrations of OC functional groups present in the Qtz treatments were subtracted from concentrations of OC associated with the Mn oxide- and Fe oxide-coated sands (Figure 4; see Supporting Methods for calculations). Organic C that was associated with Mn oxide had a high proportion of O-alkyl-C (42.4 ± 14.3%) with moderate contributions from carboxylic-C, aliphatic-C, and aromatic-C (15–30%) and smaller contributions of phenolic-C and quinone-C (<10%) (Figure 4). Organic C associated with Fe oxide had the highest proportion of carboxylic-C (26.1 ± 0.014%) with less o-alkyl-C (19.9 ± 0.026%) but more phenolic-C (15.0 ± 0.5%) than Mn oxide (Figure 4). Aliphatic-C, aromatic-C, and quinone-C were present in similar proportions (∼10–20%) for the Mn oxide and Fe oxide. In comparison, leaf litter from which the OC that is associated with surface-incubated mineral bags presumably derived had higher proportions of aromatic C (27.1 ± 0.9%) and carboxylic C (19.8 ± 1.0%) and lower proportions of O-alkyl C (7.82 ± 0.29%) (Figure 4). Carbonate peaks (∼300 to 301 eV)^92^ were not present in the spectra, indicating the absence of carbonate formation during incubation (Figure S12).

**Figure 4:**
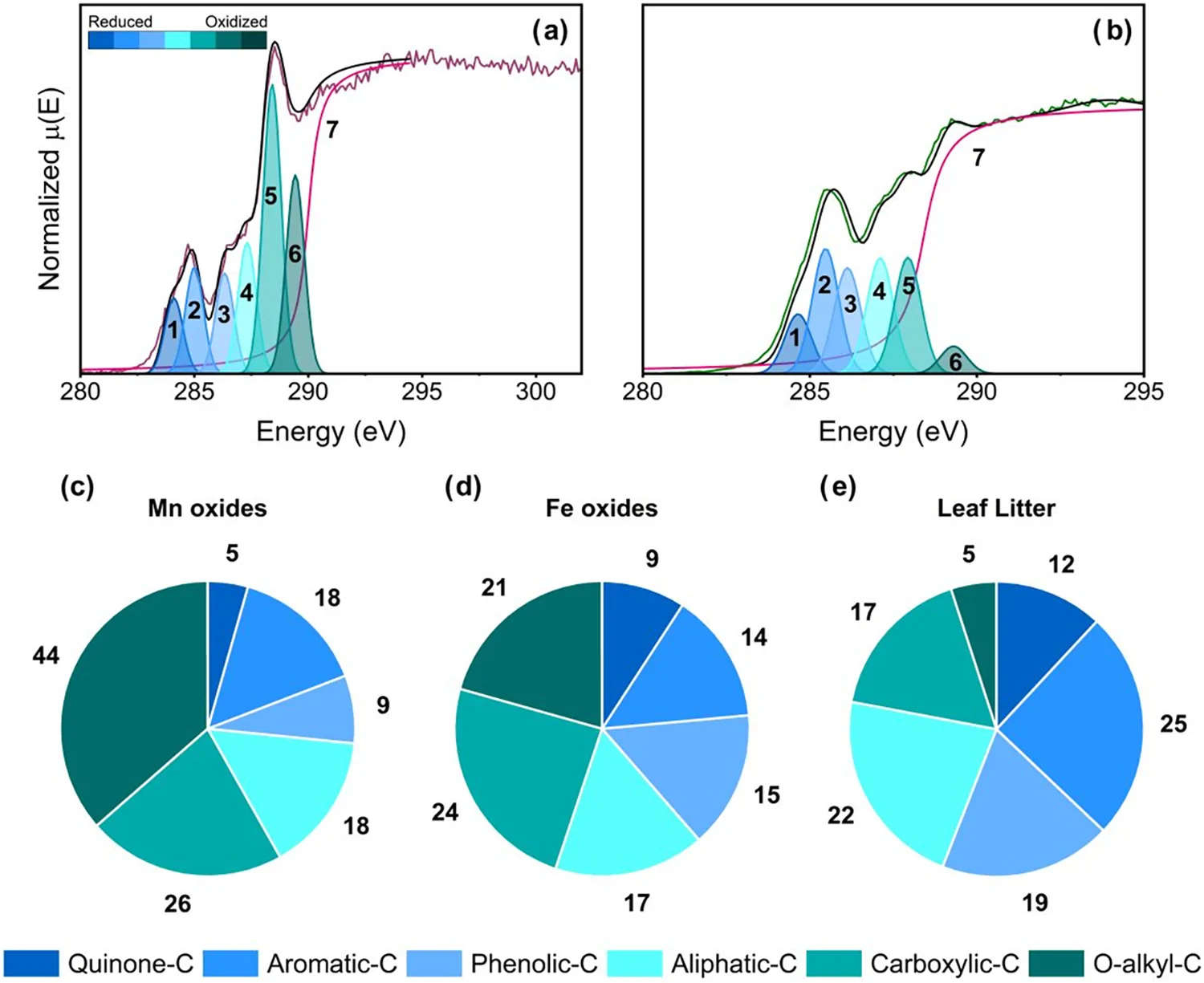
Organic C functional groups of organic matter associated with Mn and Fe oxides differed from the overlying leaf litter. Panels (a) and (b) show example NEXAFS spectra for the mineral bags after incubation under the leaf litter for 365 days and the leaf litter, respectively. Each Gaussian function corresponds to an OC functional group: (1) quinone-C; (2) aromatic-C; (3) phenolic-C; (4) aliphatic-C; (5) carboxylic-C; (6) O-alkyl-C; (7) arctangent function. The Gaussian peak colors match the colors in the pie graphs. The OC functional groups associated with the Mn oxide (c) and Fe oxide (d) coatings after 365 days and the leaf litter (e) are shown in the pie graphs with the percentage of each functional group next to the corresponding wedge in the graph (percentages with error presented in Table S26). Note that the composition of organic matter associated with Fe and Mn oxides, as shown in (c) and (d), excludes contributions of background organic matter that also collected in quartz treatments.

Organic matter on the surface (∼10 nm depth; measured with XPS) of the Qtz, Mn oxide- and Fe oxide-coated sand also showed an increase in oxidized OC over time (Figure S14). Initial materials were dominated by reduced C (C–C/C-H), while incubated minerals had higher proportions of polysaccharide/amino C (C–O/C-N) and oxidized C (C=O) groups. As such, both bulk and surface OC analyses indicate that initial minerals contained low concentrations of TOC consistent with reduced, aliphatic compounds, while incubated minerals contained ∼4–10× more total OC (Table S19) with higher proportions of oxidized C associated with Mn and Fe oxides.

Carbon was colocated with Mn but not Si in the Mn oxide-coated sands (Figure 2), indicating that OC that accumulated over time was associated with the Mn oxide coating and not the quartz. Carbon was similarly associated with Fe in the Fe oxide-coated sands but was present in small clusters, while Fe was more diffusely distributed across the quartz grain. Carbon on the incubated Qtz was concentrated on the rough, angular edges of the Qtz grain as small globular structures, with less C spread across the smoother surfaces, suggesting that organic matter was transported into the mesh bags but did not associate with the quartz surface. Small quantities of Fe were also detected on the Qtz with SEM/EDS but were not associated with increased C (Figure S8).

Small-angle neutron scattering (SANS) was employed to examine the characteristics of the minerals and determine whether the TOC was on the surface of the oxides or within pore spaces. The low Q (0.06–0.01 Å^–1^) represents inelastic scattering, while the middle (0.01–0.1 Å^–1^) and high Q (0.1–0.6 Å^–1^) ranges represent nanosized and larger features, respectively, such as particles or pores. Fe oxide-coated sand had increased intensity in the middle Q range (0.02–0.2 Å^–1^) relative to Qtz, indicative of Fe oxide nanoparticles sized ∼10 nm (Figure 5, Table S29). Spectra for the initial Mn oxide-coated sand and Qtz were similar, reflecting the large particle size for both the quartz (50–200 μm) and for the Mn oxide particles (∼450 nm)^93^ that fall outside the resolution range of the SANS technique. Increasing intensities between days 0 and 365 in the high Q and middle Q ranges (Figure 5, Table S29) for the uncoated and Mn oxide-coated sand are attributed to an increase in sorbed organic matter that contains hydrogen, which contributes to the signal. Fe oxide-coated sand had the same increase in intensity (0.004) as the Mn oxide-coated sand at high Q. The small increase at high Q, which likely reflects an increase in sorbed organic matter, is apparent despite the high initial incoherent scattering due to the high hydration of the Fe oxide. A small decrease in intensity and shift to lower Q values at ∼0.1 Å^–1^ in the Fe oxide treatment is consistent with an apparent increase in the particle size of the Fe oxide nanoparticles, potentially due to pore infilling by organic matter.

**Figure 5:**
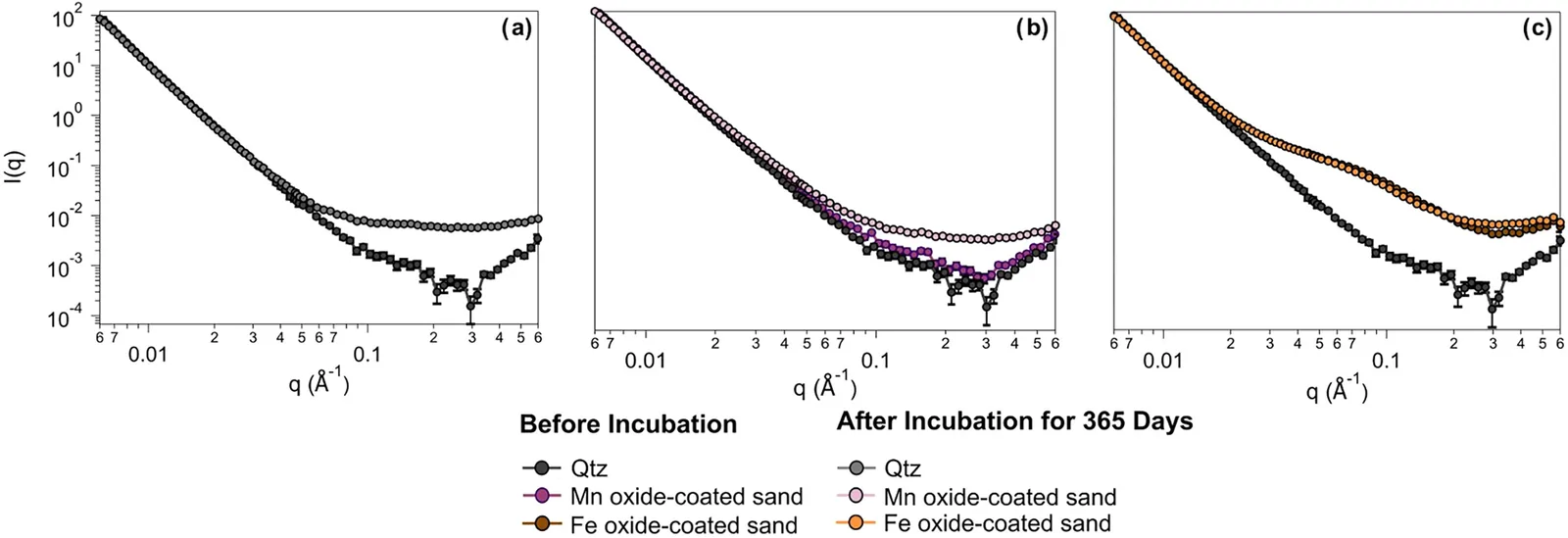
Increased neutron scattering intensity indicates organic matter increased over time for the Qtz, Mn oxide- and Fe oxide-coated sand. Small-angle neutron scattering (SANS) results for the Qtz (a), Mn oxide-coated sand (b), and Fe oxide-coated sand (c). The curves are log–log plots where *I*(*Q*) is scattering intensity, and *Q* is the scattering vector calculated using neutron wavelength (λ) and scattering angle. The intensity at high Q increased for all three treatments. The Fe oxide-coated sand has the smallest intensity increase after one year due to high initial scattering derived from the hydrated mineral.

## Discussion

The objective of this study was to evaluate the relative capacities of Mn and Fe oxides to sequester organic C in soil environments. To accomplish this objective, synthetic, C-poor Mn and Fe oxide minerals coated onto quartz sand were incubated in forest soils for up to one year. The quantity and composition of organic matter that was associated with these minerals were evaluated over time. Contrary to our hypothesis, we observed that Mn oxides retained more total organic C after one year than Fe oxides despite also having higher reactivity. As described below, the higher retention and/or reaction of organic C with Mn oxides may have been facilitated by Ca bridging, even in this acidic, cation-depleted forest soil.

While *in situ* field incubations are a valuable middle ground between a laboratory study and observational data from the field, this experimental design has its own limitations. The field incubation allowed for a direct comparison of OC interactions with Mn and Fe oxides under environmental conditions that are difficult to replicate in a laboratory setting. However, it is not possible to determine the exact mechanisms driving the observed results without additional controlled laboratory experiments. Therefore, we propose potential mechanisms based on our observations that lay the groundwork for future research into the interactions of OCs with Mn and Fe oxides that occur naturally in soil environments.

### Mineral Reactivity during Incubation

Manganese and Fe oxide concentrations did not change over time, indicating that Mn and Fe were retained on the sands rather than being leached into the surrounding soil. However, temporary increases in water-extractable Mn may indicate release of dissolved Mn^2+^ following Mn­(III/IV) reduction coupled with OC oxidation.^17,19,20,94^ Water-extractable OC from the Mn oxide-coated sand was significantly correlated with water-extractable Mn (Figure S11), suggesting the release of dissolved Mn^2+^ and small organic compounds as reaction products. Manganese oxides are well-known to undergo dissolution during reaction with organic compounds.^17,19,20,33,38,94^ Increases in bulk Mn AOS over time in the Mn oxide-coated sand treatments are consistent with a net decrease in Mn­(II) and an increase in the Mn­(IV) content. Reductive Mn dissolution likely resulted in preferential removal of reduced Mn from the initial Mn oxide,^14–16,95,96^ leaving primarily Mn­(IV). In addition, Mn AOS at the mineral surface decreased over time, consistent with an accumulation of minor amounts of Mn­(III) species. Mn­(II) has a strong affinity for sorption to Mn oxides,^78,97–100^ and small quantities of dissolved Mn­(II) released from Mn oxide likely resorbed on the Mn oxide surface. Several studies have suggested that Mn­(II) and Mn­(IV) comproportionate into Mn­(III) when Mn­(II) is adsorbed to Mn oxides,^37,93,101^ explaining the increase in Mn­(III) content at the surface. Alternatively, the increase of Mn­(III) at the surface could indicate the partial reduction of Mn­(IV). It is also possible that Mn^2+^ may derive from the surrounding soil instead of the incubated mineral (Table S1), with net inputs balancing net losses.

Unlike the Mn oxide-coated sand, water-extractable Fe remained below detection, and the Fe­(II) and Fe­(III) speciation of the bulk Fe oxide-coated sand did not change over time (Tables S7 and S10). Slight increases in Fe­(II) on the surface of the mineral suggest limited reactivity with OC, as has been demonstrated in previous studies;^102^ however, adsorption of OC to Fe oxide appeared to be greater than Fe oxide reactivity with OC in this study. Although Fe concentrations in Qtz remained below detection over time (Table S7), C and Fe were colocated in SEM images (Figure S8) of the Qtz sand incubated for 365 days, suggesting precipitation of small quantities of Fe oxides and adsorption of OC or coprecipitation of Fe and OC.

### Organic Matter Accumulation

Synthetic Mn oxide accumulated more OC per unit surface area (mg C m^–2^) than Fe oxide when incubated in forest soil for up to one year (Figures 1 and S9). This was true of OC normalized to either reported SSA^17^ or ESSA calculated for the oxides coated onto sand (Figures 1 and S9). Organic C still accumulated in Mn oxide treatments despite evidence for reactivity, indicating that some reaction products associated with the mineral surface or that additional organic compounds sorbed without undergoing reaction. From that perspective, Mn oxides have a greater potential to store OC compared to Fe oxides, but the total C storage in soil would depend on the relative abundance of each mineral.

The concentrations of total OC associated with Mn oxide in this study agree with concentrations reported for synthetic and biogenic Mn oxides incubated in brackish pond water^41^ but were lower than experiments that reacted OC with Mn oxides.^17,33,103^ In comparison, quantities of OC associated with Fe oxides were similar to previous studies^13,51,52,104^ although 2 orders of magnitude lower than maximum reported OC:Fe ratios.^105^ The relatively low amounts of organic matter associated with metal oxides in this study may reflect low soil C content (Table S2) and/or limited transport of organic matter into the mineral bags compared to experimental studies in which OC is relatively abundant. Water-extractable organic carbon is inferred to have derived from interactions between nonwater-soluble organic matter and the metal oxides. Although it is possible that WEOC was transported into the mineral bags from the surrounding soil or derived from microbial metabolites, WEOC concentrations were higher for oxide treatments than for Qtz and were particularly high for Mn oxide, reflecting its higher reactivity.

While the small quantities of OC in the initial materials were highly aliphatic, all treatments accumulated oxygen-containing functional groups during the incubation. Organic C that was associated with Mn oxide and Fe oxides was particularly enriched in oxygen-containing carboxylic-C and O-alkyl-C groups relative to the OC species associated with the uncoated Qtz (Figures 4, S13 and S14). Our findings agree with previous research that shows carboxylic-C associates with Fe oxides^32,49–51,106^ and Mn oxides^32,38,41^ and suggests that ligand binding may be an important OC adsorption mechanism.^33,38,49,50,103^ O-alkyl-C can be interpreted as polysaccharides, alcohols, and ethers.^88^ Estes et al.^41^ reported higher relative proportions of O-alkyl-C associated with Mn oxides in fungal cultures (42.89 ± 2.85%), which agrees with O-alkyl-C values reported in this study (42.4 ± 14.3%; Figure 4). Although the mesh size (5 μm) should exclude roots and larger hyphae, small hyphae and microorganisms were likely present in the mineral bags. The composition of the OC that accumulated in the bags was distinct from the overlying leaf litter, which was relatively enriched in quinone, aromatic, and phenolic compounds (Figure 4). The OC that accumulated in the bags buried under the litter layer likely reflects the more soluble, oxygenated compounds that leach from the litter,^6^ while OC that associated with the metal oxides may have undergone additional selective adsorption and/or (a)­biotic transformation.^6,31,32,38,107^

Iron oxides had visibly fewer surface pores than Mn oxides following incubation (Figure S1), which could potentially be explained by infilling with organic matter. Previous studies have shown that OC can accumulate in pores of Fe oxides,^48,51,106,108^ and decreasing intensity of nanometer-sized features in SANS may reflect this infilling (Figure 5, Table S29). Although previous research has also reported OC in Mn oxide pores,^33^ the relatively unchanged appearance of the pores and uniform accumulation across different-sized features (Figure S1) suggests that OC accumulation in pore spaces was not a dominant storage mechanism for Mn oxide in this study.

### Factors Promoting Organo-Mineral Associations

Water-extractable Ca concentrations increased over time for the Mn-oxide-coated sands and the Fe-oxide-coated sands, suggesting that Ca^2+^ ions from the surrounding soil solution sorbed to the oxide surfaces and bound OC to the minerals via cation bridging. Although well-recognized as an important OC stabilization mechanism in more alkaline soils,^109^ Ca cation bridging is a potentially overlooked contributor to C storage in acidic soils. Greater concentrations of water-soluble Ca were associated with the Mn oxide-coated sand than with Fe oxide-coated sand (Figure 1), indicating that cation-bridging may be a more important mechanism for OC association with Mn oxide. Indeed, colocation of Mn and Ca (Figure 2) but not Fe and Ca supports this conclusion, and Ca has been previously reported to increase adsorption of OC to Mn oxides in both laboratory and field studies.^33,61,110,111^ Calcium may also be coprecipitating with OC during the formation of fresh Mn oxides.^61^ Similar correlations between Ca, OC, and Mn oxides were observed in a mineral bag study where Mn oxides precipitated during vermiculite weathering.^61^ Alternatively, Ca may associate with negatively charged Mn oxide but not facilitate OC sorption, and future studies are needed to differentiate between these two processes.

Sorbed Ca may also promote cation bridging between OC and Fe oxides or direct bonding between the Fe oxide surface and carboxylic functional groups,^49,50^ albeit at a lower level than for Mn oxides. Ca significantly increased over time on the Fe oxide-coated sand (Figure 1) but plateaued at a lower concentration than that for Mn oxide-coated sand. In addition, there was only a weak correlation between the spatial distributions of Fe and Ca on the Fe oxide-coated sand (Figure S7), indicating that Ca may have facilitated initial OC sorption but was not retained on the Fe oxide surface. Alternatively, Fe–Ca associations may not have been captured by the small area probed with microscopy techniques. Regardless, while Ca likely promoted organo-mineral associations for both oxides, we infer that Ca plays a greater role in OC stabilization for Mn oxides than Fe oxides, possibly because Mn oxide surfaces are more negatively charged than Fe oxide surfaces in moderately acidic conditions.^112^ The contribution of Ca to OC stabilization occurred despite the minerals being incubated in a highly weathered Ultisol that is depleted in base cations.^72^

### Implications for Soil Environments

Although Mn concentrations are generally lower than Fe concentrations in soils, Mn oxides may play an outsized role in the OC transformation and stabilization. Our study demonstrates that the poorly crystalline Mn oxides have the potential to sequester more OC than the analogous poorly crystalline Fe oxides in a temperate forest soil, particularly in surface soils where Mn oxides can accumulate.^29^ Ferrihydrite and other Fe oxides may exert more influence over C dynamics in subsoils and at redox interfaces where Fe accumulates and can bind OM or oxidize OM through Fenton reactions.^31,107,113^ In this study, OC associated with ferrihydrite incubated in surface soils through sorption and/or cation bridging but showed minor signs of reaction with the mineral surface. In comparison, several mechanisms, including OM oxidation by Mn, are likely to contribute to OM stabilization by Mn oxide. Briefly, nonreactive OM can bind to the mineral surface directly or through electrostatic interactions. Sorption and subsequent oxidation of reactive OM by the Mn oxide surface produces dissolved Mn^2+^ that reprecipitates as fresh Mn­(IV) oxides, trapping adsorbed OM and providing additional surface area for OM to accumulate. Although OM can also react with Fe oxides, Mn oxides have a higher redox potential and are generally more reactive toward organic compounds. In addition, this research indicates that Ca is important for OM sequestration in acidic environments. Cation bridging between OM and the Mn oxide surface likely facilitates sorption that can either stabilize an organic compound against decomposition or promote electron transfer that leads to OM oxidation. Calcium may also facilitate OM sorption to Fe oxide through Fe–Ca–OM ternary complexes; however, Ca was observed to associate primarily with Mn oxides in this study.

Manganese may thus exert critical control on C retention and transformation in surface soils where both OM and Mn accumulate,^25,114^ with implications for soil C storage. Previous research has demonstrated that reactive Mn­(III) intermediates produced through microbial activity drive OM oxidation in surface soils^24,31^ and may decrease long-term C storage across diverse ecosystems.^25,115–117^ Manganese oxides form as a byproduct of microbial Mn­(III) cycling and accumulate in surface soils as Mn-rich leaf litter decomposes.^24^ This study demonstrates that Mn oxides that form in soils may both oxidize organic compounds, likely producing small molecules and CO_2_, and also bind and stabilize organic matter more effectively than Fe oxides. Interactions between Mn oxides and OM may be particularly important in acidic soils where Mn–OM reactions are favored and where enhanced Mn biocycling by plants increases Mn accumulation in surface soils.^25,114^

## Supplementary Material





## Data Availability

Data is available in Supporting Information and accessed via https://data.ess-dive.lbl.gov/datasets/doi:10.15485/3412618.

## Notes

Additional methods and calculations; soil properties from the field site where mineral bags were incubated; additional graphs and data tables (PDF)
Fe and Mn XANES and C NEXAFS spectra; and neutron scattering spectra (XLSX)

## ¶

Department of Sustainable Earth Systems Sciences, The University of Texas at Dallas, Richardson, Texas 75080, United States

## References

[1] Lehmann J., Kleber M. (2015). The contentious nature of soil organic matter. Nature.

[2] Shi Z., Allison S. D., He Y., Levine P. A., Hoyt A. M., Beem-Miller J., Zhu Q., Wieder W. R., Trumbore S., Randerson J. T. (2020). The age
distribution of global soil carbon inferred
from radiocarbon measurements. Nat. Geosci..

[3] Blanco-Canqui H., Lal R. (2004). Mechanisms of Carbon Sequestration in Soil Aggregates. Crit. Rev. Plant Sci..

[4] Wiesmeier M., Urbanski L., Hobley E., Lang B., von Lützow M., Marin-Spiotta E., van Wesemael B., Rabot E., Ließ M., Garcia-Franco N., Wollschläger U., Vogel H.-J., Kögel-Knabner I. (2019). Soil organic
carbon storage as a key function of soils - A review of drivers and
indicators at various scales. Geoderma.

[5] Hall S. J., Ye C., Weintraub S. R., Hockaday W. C. (2020). Molecular trade-offs in soil organic
carbon composition at continental scale. Nat.
Geosci..

[6] Kleber M., Eusterhues K., Keiluweit M., Mikutta C., Mikutta R., Nico P. S. (2015). Mineral–Organic
Associations: Formation, Properties,
and Relevance in Soil Environments. Adv. Agron..

[7] Kramer M. G., Chadwick O. A. (2018). Climate-driven thresholds in reactive mineral retention
of soil carbon at the global scale. Nat. Clim.
Change.

[8] Lalonde K., Mucci A., Ouellet A., Gélinas Y. (2012). Preservation
of organic matter in sediments promoted by iron. Nature.

[9] Slessarev E. W., Chadwick O. A., Sokol N. W., Nuccio E. E., Pett-Ridge J. (2022). Rock weathering
controls the potential for soil carbon storage at a continental scale. Biogeochemistry.

[10] Zhao Q., Poulson S. R., Obrist D., Sumaila S., Dynes J. J., McBeth J. M., Yang Y. (2016). Iron-bound
organic carbon in forest
soils: quantification and characterization. Biogeosciences.

[11] Rasmussen C., Heckman K., Wieder W. R., Keiluweit M., Lawrence C. R., Berhe A. A., Blankinship J. C., Crow S. E., Druhan J. L., Hicks Pries C. E., Marin-Spiotta E., Plante A. F., Schädel C., Schimel J. P., Sierra C. A., Thompson A., Wagai R. (2018). Beyond clay:
towards an improved set of variables for predicting soil organic matter
content. Biogeochemistry.

[12] Keiluweit M., Bougoure J. J., Nico P. S., Pett-Ridge J., Weber P. K., Kleber M. (2015). Mineral protection
of soil carbon
counteracted by root exudates. Nat. Clim. Change.

[13] Eusterhues K., Hädrich A., Neidhardt J., Küsel K., Keller T. F., Jandt K. D., Totsche K. U. (2014). Reduction of ferrihydrite
with adsorbed and coprecipitated organic matter: microbial reduction
by *Geobacter bremensis* vs. abiotic reduction by Na-dithionite. Biogeosciences.

[14] Duckworth O. W., Sposito G. (2005). Siderophore–Manganese­(III)
Interactions. I.
Air-Oxidation of Manganese­(II) Promoted by Desferrioxamine B. Environ. Sci. Technol..

[15] Duckworth O. W., Sposito G. (2005). Siderophore–Manganese­(III)
Interactions II.
Manganite Dissolution Promoted by Desferrioxamine B. Environ. Sci. Technol..

[16] Duckworth O. W., Sposito G. (2007). Siderophore-promoted
dissolution of synthetic and biogenic
layer-type Mn oxides. Chem. Geol..

[17] Li H., Reinhart B., Moller S., Herndon E. (2023). Effects of C/Mn Ratios
on the Sorption and Oxidative Degradation of Small Organic Molecules
on Mn-Oxides. Environ. Sci. Technol..

[18] Remucal C. K., Ginder-Vogel M. (2014). A critical
review of the reactivity of manganese oxides
with organic contaminants. Environ. Sci. Process
Impacts.

[19] Stone A. T., Morgan J. J. (1984). Reduction and dissolution of manganese (III) and manganese
(IV) oxides by organics: 2. Survey of the reactivity of organics. Environ. Sci. Technol..

[20] Stone A. T., Morgan J. J. (1984). Reduction and dissolution
of manganese (III) and manganese
(IV) oxides by organics. 1. Reaction with hydroquinone. Environ. Sci. Technol..

[21] Herndon E. M., Jin L., Andrews D. M., Eissenstat D. M., Brantley S. L. (2015). Importance of vegetation
for manganese cycling in temperate forested watersheds. Global Biogeochem. Cycles.

[22] Herndon E. M., Martínez C. E., Brantley S. L. (2014). Spectroscopic (XANES/XRF) characterization
of contaminant manganese cycling in a temperate watershed. Biogeochemistry.

[23] Jobbágy E. G., Jackson R. B. (2004). The Uplift of Soil
Nutrients by Plants: Biogeochemical
Consequences Across Scales. Ecology.

[24] Keiluweit M., Nico P., Harmon M. E., Mao J., Pett-Ridge J., Kleber M. (2015). Long-term litter decomposition controlled by manganese
redox cycling. Proc. Natl. Acad. Sci. U.S.A..

[25] Santos F., Herndon E. (2023). Plant-Soil Relationships Influence Observed Trends
Between Manganese and Carbon Across Biomes. Global Biogeochem. Cycles.

[26] Herndon E. M., Lixin J., Brantley S. (2011). Soils Reveal
Widespread Manganese
Enrichment from Industrial Inputs. Environ.
Sci. Technol..

[27] Weil, R. R. ; Brady, N. C. The Nature and Properties of Soils; Pearson Education Limited, 2017.

[28] Sparks, D. L. Environmental Soil Chemistry; Elsevier, 2003.

[29] Li H., Santos F., Butler K., Herndon E. (2021). A Critical Review on
the Multiple Roles of Manganese in Stabilizing and Destabilizing Soil
Organic Matter. Environ. Sci. Technol..

[30] Tebo B. M., Bargar J. R., Clement B. G., Dick G. J., Murray K. J., Parker D., Verity R., Webb S. M. (2004). Biogenic Manganese
Oxides: Properties and Mechanisms of Formation. Annu. Rev. Earth Planet. Sci..

[31] Jones M. E., LaCroix R. E., Zeigler J., Ying S. C., Nico P. S., Keiluweit M. (2020). Enzymes, Manganese,
or Iron? Drivers of Oxidative Organic
Matter Decomposition in Soils. Environ. Sci.
Technol..

[32] Chorover J., Amistadi M. K. (2001). Reaction of forest floor organic matter at goethite,
birnessite and smectite surfaces. Geochim. Cosmochim.
Acta.

[33] Allard S., Gutierrez L., Fontaine C., Croue J. P., Gallard H. (2017). Organic matter
interactions with natural manganese oxide and synthetic birnessite. Sci. Total Environ..

[34] Joshi T. P., Zhang G., Cheng H., Liu R., Liu H., Qu J. (2017). Transformation of para arsanilic
acid by manganese oxide: Adsorption,
oxidation, and influencing factors. Water Res..

[35] Wang X., Yao J., Wang S., Pan X., Xiao R., Huang Q., Wang Z., Qu R. (2018). Phototransformation
of estrogens
mediated by Mn­(III), not by reactive oxygen species, in the presence
of humic acids. Chemosphere.

[36] Cheng H., Yang T., Jiang J., Lu X., Wang P., Ma J. (2020). Mn­(2+) effect on manganese oxides
(MnOx) nanoparticles aggregation
in solution: Chemical adsorption and cation bridging. Environ. Pollut..

[37] Zhu M., Ginder-Vogel M., Parikh S. J., Feng X.-H., Sparks D. L. (2010). Cation
Effects on the Layer Structure of Biogenic Mn-Oxides. Environ. Sci. Technol..

[38] Johnson K., Purvis G., Lopez-Capel E., Peacock C., Gray N., Wagner T., Marz C., Bowen L., Ojeda J., Finlay N., Robertson S., Worrall F., Greenwell C. (2015). Towards a
mechanistic understanding of carbon stabilization in manganese oxides. Nat. Commun..

[39] Wang Z., Zhao H., Shi Z., Zhao H., Chen S., Chen Z., Yuan Y., Zhang C., Jia B., Jia H. (2025). Manganese Dioxides Induce the Transformation and Protection of Dissolved
Organic Matter Simultaneously: A Significance of Crystallinity. Environ. Sci. Technol..

[40] Totsche K. U., Amelung W., Gerzabek M. H., Guggenberger G., Klumpp E., Knief C., Lehndorff E., Mikutta R., Peth S., Prechtel A., Ray N., Kögel-Knabner I. (2018). Microaggregates in soils. J.
Plant Nutr. Soil Sci..

[41] Estes E. R., Andeer P. F., Nordlund D., Wankel S. D., Hansel C. M. (2017). Biogenic
manganese oxides as reservoirs of organic carbon and proteins in terrestrial
and marine environments. Geobiology.

[42] González F., Somoza L., Lunar R., Martínez-Frías J., Rubí J. A. M., Torres T., Ortiz J. E., Díaz-del-Río V. (2010). Internal features,
mineralogy and geochemistry of ferromanganese nodules from the Gulf
of Cadiz: The role of the Mediterranean Outflow Water undercurrent. J. Mar. Syst..

[43] Moore O. W., Curti L., Woulds C., Bradley J. A., Babakhani P., Mills B. J. W., Homoky W. B., Xiao K.-Q., Bray A. W., Fisher B. J., Kazemian M., Kaulich B., Dale A. W., Peacock C. L. (2023). Long-term organic
carbon preservation enhanced by iron
and manganese. Nature.

[44] Rennert T., Händel M., Höschen C., Lugmeier J., Steffens M., Totsche K. U. (2014). A NanoSIMS
study on the distribution of soil organic
matter, iron and manganese in a nodule from a S tagnosol. Eur. J. Soil Sci..

[45] Gu B., Schmitt J., Chen Z., Liang L., McCarthy J. F. (1994). Adsorption
and desorption of natural organic matter on iron oxide: mechanisms
and models. Environ. Sci. Technol..

[46] Gu B., Schmitt J., Chen Z., Liang L., McCarthy J. F. (1995). Adsorption
and desorption of different organic matter fractions on iron oxide. Geochim. Cosmochim. Acta.

[47] Kaiser K., Mikutta R., Guggenberger G. (2007). Increased
Stability of Organic Matter
Sorbed to Ferrihydrite and Goethite on Aging. Soil Sci. Soc. Am. J..

[48] Mikutta C., Lang F., Kaupenjohann M. (2004). Soil Organic
Matter Clogs Mineral
Pores. Soil Sci. Soc. Am. J..

[49] Sowers T. D., Adhikari D., Wang J., Yang Y., Sparks D. L. (2018). Spatial
Associations and Chemical Composition of Organic Carbon Sequestered
in Fe, Ca, and Organic Carbon Ternary Systems. Environ. Sci. Technol..

[50] Sowers T. D., Stuckey J. W., Sparks D. L. (2018). The synergistic
effect of calcium
on organic carbon sequestration to ferrihydrite. Geochem. Trans..

[51] Chen C., Dynes J. J., Wang J., Sparks D. L. (2014). Properties of Fe-Organic
Matter Associations via Coprecipitation versus Adsorption. Environ. Sci. Technol..

[52] Eusterhues K., Wagner F. E., Häusler W., Hanzlik M., Knicker H., Totsche K. U., Kögel-Knabner I., Schwertmann U. (2008). Characterization
of ferrihydrite-soil organic matter coprecipitates by X-ray diffraction
and Mossbauer spectroscopy. Environ. Sci. Technol..

[53] Boujelben N., Bouzid J., Elouear Z., Feki M. (2010). Retention of nickel
from aqueous solutions using iron oxide and manganese oxide coated
sand: kinetic and thermodynamic studies. Environ.
Technol..

[54] Kan C.-C., Aganon M. C., Futalan C. M., Dalida M. L. P. (2013). Adsorption
of
Mn2+ from aqueous solution using Fe and Mn oxide-coated sand. J. Environ. Sci..

[55] Mažeikienė A., Vaišku̅naitė R., Šarko J. (2021). Sand from
groundwater treatment coated with iron and manganese used for phosphorus
removal from wastewater. Sci. Total Environ..

[56] Tiwari D., Yu M. R., Kim M. N., Lee S. M., Kwon O. H., Choi K. M., Lim G. J., Yang J. K. (2007). Potential application
of manganese coated sand in the removal of Mn­(II) from aqueous solutions. Water Sci. Technol..

[57] Wu K., Liu R., Liu H., Zhao X., Qu J. (2011). Arsenic­(III,V) Adsorption
on Iron-Oxide-Coated Manganese Sand and Quartz Sand: Comparison of
Different Carriers and Adsorption Capacities. Environ. Eng. Sci..

[58] Yang J. K., Song K. H., Kim B. K., Hong S. C., Cho D. E., Chang Y. Y. (2007). Arsenic removal by iron and manganese coated sand. Water Sci. Technol..

[59] Fernandez I. J., Rustad L. E., Norton S. A., Kahl J. S., Cosby B. J. (2003). Experimental
Acidification Causes Soil Base-Cation Depletion at the Bear Brook
Watershed in Maine. Soil Sci. Soc. Am. J..

[60] Mitchell M. J., David M., Fernandez I., Fuller R., Nadelhoffer K., Rustad L., Stam A. (1994). Response of buried mineral soil bags
to experimental acidification of forest ecosystem. Soil Sci. Soc. Am. J..

[61] Van
Der Kellen I., Derrien D., Ghanbaja J., Turpault M.-P. (2022). Recent
weathering promotes C storage inside large phyllosilicate particles
in forest soil. Geochim. Cosmochim. Acta.

[62] Barczok M., Smith C., Di Domenico N., Kinsman-Costello L., Singer D., Herndon E. (2023). Influence of contrasting
redox conditions
on iron (oxyhydr)­oxide transformation and associated phosphate sorption. Biogeochemistry.

[63] Barczok M., Smith C., Kinsman-Costello L., Patzner M., Bryce C., Kappler A., Singer D., Herndon E. (2024). Iron transformation
mediates phosphate retention across a permafrost thaw gradient. Commun. Earth Environ..

[64] Patzner M. S., Kainz N., Lundin E., Barczok M., Smith C., Herndon E., Kinsman-Costello L., Fischer S., Straub D., Kleindienst S., Kappler A., Bryce C. (2022). Seasonal Fluctuations
in Iron Cycling in Thawing Permafrost Peatlands. Environ. Sci. Technol..

[65] NEON . Oak Ridge NEON/ORNL 2025 https://www.neonscience.org/field-sites/ornl (accessed Dec 12, 2024).

[66] Lietzke, D. Soils of Walker Branch Watershed; ORNL Oak Ridge National Laboratory: US, 1994.

[67] Lutz B. D., Mulholland P. J., Bernhardt E. S. (2012). Long-term
data reveal patterns and
controls on stream water chemistry in a forested stream: Walker Branch,
Tennessee. Ecol. Monogr..

[68] Luxmoore R. J., Grizzard T., Patterson M. (1981). Hydraulic
properties of Fullerton
cherty silt loam. Soil Sci. Soc. Am. J..

[69] Mulholland P. J. (1993). Hydrometric
and stream chemistry evidence of three storm flowpaths in Walker Branch
Watershed. J. Hydrol..

[70] Wilson G., Jardine P., Luxmoore R., Zelazny L., Lietzke D., Todd D. (1991). Hydrogeochemical processes
controlling subsurface transport from
an upper subcatchment of Walker Branch watershed during storm events.
1. Hydrologic transport processes. J. Hydrol..

[71] Johnson, D. W. ; Cole, D. ; Horng, F. ; Van Miegroet, H. ; Todd, D. Chemical Characteristics of Two Forested Ultisols and Two Forested Inceptisols Relevant to Anion Production and Mobility, ORNL/TM-7646, Union Carbide Corp. Nuclear Division, Oak Ridge National Lab 1981.

[72] Peters, L. ; Grigal, D. ; Curlin, J. ; Selvidge, W. Walker Branch Watershed Project: Chemical, Physical and Morphological Properties of the Soils of Walker Branch Watershed Oak Ridge National Lab: Tennessee; 1970.

[73] Grizzard, T. ; Henderson, G. ; Clebsch, E. ; Reichle, D. Seasonal Nutrient Dynamics of Foliage and Litterfall on Walker Branch Watershed, A Deciduous Forest Ecosystem Oak Ridge National Lab: Tennessee; 1976.

[74] Johnson D. W., Todd D. W. (1990). Nutrient cycling in forests of Walker
Branch Watershed,
Tennessee: roles of uptake and leaching in causing soil changes. American Society of Agronomy, Crop Science Society of America,
and Soil Science Society of America.

[75] Johnson D. W., Henderson G. S., Todd D. E. (1988). Changes in nutrient distribution
in forests and soils of Walker Branch watershed, Tennessee, over an
eleven-year period. Biogeochemistry.

[76] Johnson D. W., Todd D. E., Trettin C. F., Sedinger J. S. (2007). Soil Carbon and
Nitrogen Changes in Forests of Walker Branch Watershed, 1972 to 2004. Soil Sci. Soc. Am. J..

[77] Johnson, D. W. ; Van Hook, R. I. Analysis of Biogeochemical Cycling Processes in Walker Branch Watershed; Springer Science & Business Media, 1989.

[78] Lafferty B. J., Ginder-Vogel M., Sparks D. L. (2010). Arsenite oxidation by a poorly crystalline
manganese-oxide 1. Stirred-flow experiments. Environ. Sci. Technol..

[79] Morgan J. J., Stumm W. (1964). Colloid-chemical properties
of manganese dioxide. J. Colloid Sci..

[80] Brooks S. C., Taylor D. L., Jardine P. M. (1996). Reactive transport of EDTA-complexed
cobalt in the presence of ferrihydrite. Geochim.
Cosmochim. Acta.

[81] Post J. E. (1999). Manganese
oxide minerals: Crystal structures and economic and environmental
significance. Proc. Natl. Acad. Sci. U.S.A..

[82] Hobara S., Koba K., Ae N., Giblin A. E., Kushida K., Shaver G. R. (2013). Geochemical Influences on Solubility of Soil Organic
Carbon in Arctic Tundra Ecosystems. Soil Sci.
Soc. Am. J..

[83] Paludan C., Jensen H. S. (1995). Sequential extraction of phosphorus in freshwater wetland
and lake sediment: significance of humic acids. Wetlands.

[84] ANDERSON B. J., Jenne E. (1970). Free-iron and-manganese oxide content of reference clays. Soil Sci..

[85] Biswas T., Gawande S. (1964). Relation of manganese
in genesis of catenary soils. J. Indian Soc.
Soil Sci..

[86] Daniels R.
B., Brasfield J., Riecken F. (1962). Distribution of Sodium Hydrosulfite
Extractable Managanese in Some Iowa Soil Profiles. Soil Sci. Soc. Am. J..

[87] Ravel B., Newville M. (2005). ATHENA, ARTEMIS, HEPHAESTUS:
data analysis for X-ray
absorption spectroscopy using IFEFFIT. J. Synchrotron
Radiat..

[88] Solomon D., Lehmann J., Kinyangi J., Liang B., Schäfer T. (2005). Carbon K-Edge
NEXAFS and FTIR-ATR Spectroscopic Investigation of Organic Carbon
Speciation in Soils. Soil Sci. Soc. Am. J..

[89] Manceau A., Marcus M. A., Grangeon S. (2012). Determination
of Mn valence states
in mixed-valent manganates by XANES spectroscopy. Am. Mineral..

[90] Webb, S. The MicroAnalysis Toolkit: X-ray fluorescence image processing software. In AIP Conference Proceedings; American Institute of Physics, 2011; Vol. 1365, pp 196–199.

[91] Heller W. T., Hetrick J., Bilheux J., Calvo J. M. B., Chen W.-R., DeBeer-Schmitt L., Do C., Doucet M., Fitzsimmons M. R., Godoy W. F., Granroth G. E., Hahn S., He L., Islam F., Lin J., Littrell K. C., McDonnell M., McGaha J., Peterson P. F., Pingali S. V., Qian S., Savici A. T., Shang Y., Stanley C. B., Urban V. S., Whitfield R. E., Zhang C., Zhou W., Billings J. J., Cuneo M. J., Leal R. M. F., Wang T., Wu B. (2022). drtsans: The
data reduction toolkit for small-angle neutron scattering at Oak Ridge
National Laboratory. SoftwareX.

[92] Rasmussen M. H., Jaye C., Fischer D., Weidner T. (2023). A library of calcium
mineral reference spectra recorded by parallel imaging using NEXAFS
spectromicroscopy. J. Electron Spectrosc. Relat.
Phenom..

[93] Lafferty B. J., Ginder-Vogel M., Zhu M., Livi K. J., Sparks D. L. (2010). Arsenite
oxidation by a poorly crystalline manganese-oxide. 2. Results from
X-ray absorption spectroscopy and X-ray diffraction. Environ. Sci. Technol..

[94] McBride M. B. (1987). Adsorption
and Oxidation of Phenolic Compounds by Iron and Manganese Oxides. Soil Sci. Soc. Am. J..

[95] Akafia M. M., Harrington J. M., Bargar J. R., Duckworth O. W. (2014). Metal oxyhydroxide
dissolution as promoted by structurally diverse siderophores and oxalate. Geochim. Cosmochim. Acta.

[96] Peña J., Duckworth O. W., Bargar J. R., Sposito G. (2007). Dissolution of hausmannite
(Mn3O4) in the presence of the trihydroxamate siderophore desferrioxamine
B. Geochim. Cosmochim. Acta.

[97] Eary L. E., Rai D. (1987). Kinetics of chromium
(III) oxidation to chromium (VI) by reaction
with manganese dioxide. Environ. Sci. Technol..

[98] Manning B. A., Fendorf S. E., Bostick B., Suarez D. L. (2002). Arsenic­(III) Oxidation
and Arsenic­(V) Adsorption Reactions on Synthetic Birnessite. Environ. Sci. Technol..

[99] Toner B., Manceau A., Webb S. M., Sposito G. (2006). Zinc sorption to biogenic
hexagonal-birnessite particles within a hydrated bacterial biofilm. Geochim. Cosmochim. Acta.

[100] Wang Z., Lee S.-W., Kapoor P., Tebo B. M., Giammar D. E. (2013). Uraninite oxidation and dissolution
induced by manganese
oxide: A redox reaction between two insoluble minerals. Geochim. Cosmochim. Acta.

[101] Perez-Benito J. F. (2002). Reduction of Colloidal Manganese
Dioxide by Manganese­(II). J. Colloid Interface
Sci..

[102] Possinger A. R., Zachman M. J., Dynes J. J., Regier T. Z., Kourkoutis L. F., Lehmann J. (2021). Co-precipitation induces changes
to iron and carbon chemistry and spatial distribution at the nanometer
scale. Geochim. Cosmochim. Acta.

[103] Stuckey J. W., Goodwin C., Wang J., Kaplan L. A., Vidal-Esquivel P., Beebe T. P., Sparks D. L. (2018). Impacts
of hydrous manganese oxide on the retention and lability of dissolved
organic matter. Geochem. Trans..

[104] Coward E. K., Ohno T., Plante A. F. (2018). Adsorption
and Molecular
Fractionation of Dissolved Organic Matter on Iron-Bearing Mineral
Matrices of Varying Crystallinity. Environ.
Sci. Technol..

[105] Wagai R., Mayer L. M. (2007). Sorptive stabilization of organic
matter in soils by hydrous iron oxides. Geochim.
Cosmochim. Acta.

[106] Kaiser K., Guggenberger G. (2007). Sorptive stabilization of organic
matter by microporous goethite: sorption into small pores vs. surface
complexation. Eur. J. Soil Sci..

[107] Chen C., Hall S. J., Coward E., Thompson A. (2020). Iron-mediated
organic matter decomposition in humid soils can counteract protection. Nat. Commun..

[108] Kaiser K., Guggenberger G. (2003). Mineral surfaces and soil organic
matter. Eur. J. Soil Sci..

[109] Rowley M. C., Grand S., Verrecchia É. P. (2018). Calcium-mediated stabilisation of soil organic carbon. Biogeochemistry.

[110] Tipping E., Heaton M. (1983). The adsorption of aquatic
humic substances
by two oxides of manganese. Geochim. Cosmochim.
Acta.

[111] Wang Q., Yang P., Zhu M. (2019). Effects of metal cations
on coupled birnessite structural transformation and natural organic
matter adsorption and oxidation. Geochim. Cosmochim.
Acta.

[112] Sposito G. (1998). On Points
of Zero Charge. Environ.
Sci. Technol..

[113] Hall S. J., Silver W. L. (2013). Iron oxidation stimulates organic
matter decomposition in humid tropical forest soils. Global Change Biol..

[114] Sulman B. N., Herndon E. M. (2024). Modeling Interactive
Effects of Manganese
Bioavailability, Nitrogen Deposition, and Warming on Soil Carbon Storage. J. Geophys. Res.: Biogeosci..

[115] Berg B., Erhagen B., Johansson M.-B., Nilsson M., Stendahl J., Trum F., Vesterdal L. (2015). Manganese
in the litter fall-forest floor continuum of boreal and temperate
pine and spruce forest ecosystems – A review. For. Ecol. Manage..

[116] Possinger A. R., Heckman K. A., Bowman M. M., Gallo A. C., Hatten J. A., Matosziuk L. M., Nave L. E., SanClements M. D., Swanston C. W., Weiglein T. L., Strahm B. D. (2022). Lignin and fungal
abundance modify manganese effects on soil organic carbon persistence
at the continental scale. Geoderma.

[117] Stendahl J., Berg B., Lindahl B. D. (2017). Manganese
availability
is negatively associated with carbon storage in northern coniferous
forest humus layers. Sci. Rep..

