# Supplementary material for: Manganese Oxides Incubated in Acidic Forest Soil Retain More Organic Carbon Than Iron Oxides
Source: Environ Sci Technol. 2026 Sep 1;60(36):25578–89. doi: 10.1021/acs.est.6c07327 (PMC13585057; doi:10.1021/acs.est.6c07327)
Supplement: Supplementary file 1 [file es6c07327_si_001.pdf]

# Manganese oxides incubated in acidic forest soil retain more organic carbon than iron oxides
## Supplementary Material

Kristen Bidas[1,2*] (0000-0002-4467-7107), Hui Li[1,3] (0000-0002-9374-5305), Fernanda Santos[1] (0000-0001-9155-5623), Erin C. Rooney[2,4] (0000-0002-9121-7699), Benjamin Reinhart[5] (0000-0001-5654-8823), Lilin He[6] (0000-0002-9560-8101), Changwoo Do[6] (0000-0001-8358-8417), Qian Zhao[7] (0000-0003-4489-3691), Ryan Tappero[8] (0000-0002-3560-2461), Elizabeth Herndon (0000-0002-9194-5493)[1*]

1. Environmental Sciences Division, Oak Ridge National Laboratory, Oak Ridge, Tennessee 37831, United States;
2. Department of Earth and Planetary Sciences, University of Tennessee, Knoxville, TN, 37996, United States;
3. Department of Crop and Soil Sciences, North Carolina State University, NC 27695, United States;
4. USDA-NRCS-National Soil Survey Center, Lincoln, NE, 68508, United States;
5. X-ray Science Division, Argonne National Laboratory, Lemont, Illinois, 60439, United States;
6. Neutron Scattering Division, Oak Ridge National Laboratory, Oak Ridge, Tennessee 37831, United States
7. Environmental Molecular Sciences Laboratory, Pacific Northwest National Laboratory, Richland, WA, 99354 United States
8. National Synchrotron Light Source II, Brookhaven National Laboratory, Upton, NY, 11973, United States

Corresponding authors: Kristen Bidas (kbidas@alum.utk.edu) & Elizabeth Herndon (herndonem@ornl.gov)

Running title: **Mineral-Carbon Interactions in Soils**

Submitted to *Environmental Science & Technology*

**Number of pages:** 41

**Number of Figures:** 14

**Number of Tables:** 29

Notice: This manuscript has been authored by UT-Battelle, LLC, under contract DE-AC05-00OR22725 with the US Department of Energy (DOE). The US government retains and the publisher, by accepting the article for publication, acknowledges that the US government retains a nonexclusive, paid-up, irrevocable, worldwide license to publish or reproduce the published form of this manuscript, or allow others to do so, for US government purposes. DOE will provide public access to these results of federally sponsored research in accordance with the DOE Public Access Plan (https://www.energy.gov/doe-public-access-plan).

# Supplementary Methods

**Mineral Bag Incubation**

The initial (non-coated) Qtz particles ranged in size from 50 µm to 200 µm in diameter and were angular in shape. Some areas of the Qtz were smooth and then surrounded by grooves, pits and crevices, with flakes of nano-sized Qtz present on the surface (**Figure S1**). Although the Mn oxide and Fe oxide synthesis methods were for hydrous Mn oxide[1, 2] and ferrihydrite[3] respectively, the coatings were not confirmed as hydrous Mn oxides or ferrihydrite; instead they were considered poorly crystalline oxides. Both the Mn and Fe oxide coatings were poorly crystalline with a 'fluffy' appearance and with small pores distributed throughout the oxides. In the initial coated sands, Mn oxide and Fe oxide were unevenly coated on the surfaces of the sand grains (**Figure S1**), concentrating mostly in crevices with a few nanometer-sized globules coating the smooth surfaces. Low concentrations of C were evenly distributed across the initial Qtz, Mn oxide-coated sand and Fe oxide-coated sand, consistent with the total C analyses (**Figures S2**).

**Soil Collection and Analyses**

Three soil cores were collected in August 2020 to analyze the soil characteristics of the sampling site. These cores were hand augered to 30 cm and sectioned into 0-10 cm, 10-20 cm, and 20-30 cm depth intervals. The soils were air dried upon return to the lab. Prior to analysis, soils were sieved through a 2 mm sieve and gently homogenized with a mortar and pestal. Soil pH was measured by adding 10 mL of 0.01 M $CaCl_2$ to 10 g of soil and mixing[4]. After 10 minutes the pH of the soil slurry was measured three times and the averaged recorded. Total C and N concentrations were analyzed using an Elementar Vario TOC cube (Hanau, Germany). Water-extractable cations and organic C were extracted by adding 20 mL of 0.01 M KCl to 1 g of soil and shaken for one hour on an end-over-end rotator. The soil slurry was centrifuged for 20 minutes at 4,000 x g and the supernatant filtered through 0.22 µm PES syringe filters. The supernatants were stored in either an amber vial preserved with 1%: HCl for water-extractable organic C (WEOC) analysis or 2% trace metal grade $HNO_3$ for water-extractable cation analysis.

Dithionite-extractable cations were extracted using 0.11 M Na dithionite buffered with 0.11 M $NaHCO_3$ (DB solution)[5]. One gram of soil was mixed with 25 mL of DB solution and shaken on an end-over-end rotator for one hour. After centrifuging for 10 minutes at 4,000 x g the supernatant was poured off into a separate container. The soil was rinsed once with 25 mL of DB solution, centrifuged as previously stated, and then with 25 mL deoxygenated Milli-Q $H_2O$ and centrifuged for 15 minutes at 4,000 x g. The combined supernatant extraction and rinses were combined into one container. The supernatant was filtered through a 25 mm diameter, 0.45 µm PES filter and then aerated for one hour before preserving with 5 mL of 1 M $H_2SO_4$. The addition of the BD solution and deoxygenated Milli-Q $H_2O$ were done in an anaerobic glovebag with a 95% $N_2$/5% $H_2$ atmosphere. Extracts were analyzed using a ThermoFisher Scientific iCAP 7400 ICP-OES at Oak Ridge National Laboratory for dissolved Fe, Mn, Ca, Mg, Al, P and Si.

## Mineral Bag Analyses

*Scanning Electron Microscopy/Energy-Dispersive X-ray Spectroscopy (SEM/EDS)*

SEM/EDS was done on a Merlin FE-SEM (Zeiss, Germany) at the Center for Nanophase Materials Science with a GEMINI column, an SE2 detector and an In-Lens detector, and a Bruker EDS (Bruker, Germany) and 1 nm resolution. Samples were mounted onto copper tape and imaged at 1 keV with 25% In-Lens detector signal and 75% SE2 detector signal using the SmartSEM software (Zeiss, Germany). EDS spectra were generated at 10 keV and analyzed using Bruker ESPRIT software (Bruker, Germany).

*BET Specific Surface Area*

Brunauer-Emmett-Teller (BET) specific surface area (SSA) for Qtz, Mn oxide-coated sand, and Fe oxide-coated sand were calculated using $N_2$ adsorption-desorption isotherms measured with a Micromeritics Gemini VII 2390a surface area analyzer (Georgia, USA). The initial material for each treatment was degassed at 80º C for 24 hours on a Flow Prep 60 degasser prior to measurement. The multi-point (n = 5) point regression was applied to calculate SSA.

*Mineral extractions*

Total C and nitrogen (N) concentrations for the initial and incubated mineral bags were analyzed using an Elementar Vario TOC cube (Hanau, Germany). To simulate interaction with rainwater, the initial and incubated minerals were rinsed by mixing 1 g of material with 20 mL of 0.01 M KCl[6] and shaken by hand to obtain water-extractable organic C (WEOC). The rinse was filtered through 0.1 µm PES filters into two separate containers; one filtered aliquot was acidified with trace metal grade nitric acid to ~2% $HNO_3$ for analysis on inductively coupled plasma optical emissions spectroscopy (ICP-OES), and one aliquot was acidified with trace metal grade hydrochloric acid to ~1% HCl for organic C analysis. The pellet was then shaken with 25 mL of 0.11 M Na dithionite buffered with 0.11 M $NaHCO_3$ for one hour to extract Fe and Mn[7]. The pellet was rinsed with 25 mL of the $NaHCO_3$-buffered dithionite solution and 25 mL of degassed reagent grade water. The extractant and rinses were filtered through a 25 mm diameter 0.2 µm filter, bubbled for at least an hour with air, and acidified with 5 mL of 1 M $H_2SO_4$. All solutions were added to the pellets in a glovebag under $N_2$/$H_2$ atmosphere. Each extraction included method blanks consisting of the reagents but with no added solid material. Dissolved organic C in the WEOC extracts, method blanks, and analytical blanks were measured using a Shimadzu TOC-L (Shimadzu Corp., Japan). Dissolved Fe, Mn and major cations (Ca, Mg, Al, P, Si) concentrations in the WEOC and dithionite extracts were analyzed using a ThermoFisher Scientific iCAP 7400 ICP-OES at Oak Ridge National Laboratory and with ICP-OES at the University of Georgia Extension Agricultural and Environmental Services Laboratories.

*X-ray Absorption Near Edge Structure (XANES) Spectroscopy*

XANES spectra were processed (merged, baseline corrected and normalized using the Athena software (0.9.26)[8]). The average oxidation state (AOS) and relative proportions of different oxidation states of Mn and Fe in the Mn oxide- and Fe oxide-coated sands were calculated using linear combination fitting (LCF) using Athena. Manganese and Fe XANES spectra were fit between -20 to +30 eV using single-valence reference standards. Prior to fitting the Mn reference spectra were calibrated to 6,537.7 eV and the Fe reference spectra were calibrated to 7,110.75 eV to match the calibration of the reference standards. Seventeen Mn

reference standards from Manceau, et al. [9] were used to fit Mn in the Mn oxide-coated sands: pyrolusite, ramsdellite, $Ca_2Mn_3O_8$, potassium birnessite, groutite, feitknechtite, manganite, $Mn_2O_3$, $MnPO_4$, hureaulite, fungi, rhodochrosite, manganosite, pyroxmangite, tephroite, Mn sulfate (s) and Mn sulfate (aq). Ten Fe reference standards were used to fit the AOS of Fe oxide in the Fe oxide-coated sands: goethite, hematite, 2L ferrihydrite, Fe chloride, lepidocrocite, vivanite, biotite, chlorite, FeO and Fe sulfate(s). The fits with the lowest reduced chi squared values were chosen as the best fit (**Figure S3**, **Tables S13-18**).

*Tender Energy X-ray Absorption Near Edge Structure (XANES) spectroscopy*

The XFM beamline at the National Synchrotron Light Source II (NSLS-II) typically uses Kirkpatrick-Baez (KB) mirrors to deliver microfocused X-rays from Si(111) double crystal monochromator but can be easily configured for unfocused beam (0.6 x 0.7 mm). Data collection for the "tender energy" XANES was performed in a custom helium sample environment that is isolated from a Canberra SXD-7 SDD detector array by an Ultralene$^R$ (4 um thick) window. Samples were oriented at 45° to the incident beam and the XRF detector positioned 90 to the incident beam. Data were pre-processed using Larix in the LARCH package[10]. The Ca XANES spectra were plotted with reference spectra of Ca pectate, Ca acetylacetonate, apatite, calcite, aragonite and smectite[11] in Origin Pro (OriginPro, Version 2023b. OriginLab Corporation, Northampton, MA, USA).

*Carbon Near Edge X-ray Absorption Fine Structure (NEXAFS) spectroscopy*

Gaussian peak fitting according to Solomon, et al. [12] was used to determine the organic C functional groups associated with the mineral-coated sand. The different energies for each functional group are the following: 284.3 eV C 1s-pi* transition: quinone type-C, protonated and alkylated aromatic-C; 285 eV molecular orbital of aromatic-C = protonated and alkylated to carbonyl-substituted aromatic-C; maybe olefinic-C; 286.5 eV phenolic-C; O-substituted aryl-C; 287.3 eV 1s-3p/sigma* aliphatic-C; 288.4 eV 1s-pi* transition carboxylic-C because C is electron deficient in C=O bonding; 289.3 eV 1s-pi* O-alkyl-C of polysaccharides, alcohols and ethers. Two more transitions from 1s-2π* were at 289.1 eV and 289.7 eV. While fitting the data the peak position was allowed to vary, which resulted in the following peak positions:

Arctangent 290 eV; quinone-C 284.3 eV; aromatic-C 285 eV; phenolic-C 287.3 eV; aliphatic-C 288.4 eV; carboxylic-C 289.3 eV; O-alkyl-C 289.3 eV; Sigma 1* 281.9 eV; and Sigma 2* 289.7 eV. The full width half maximum of the gaussian peaks and the arctangent were fixed at 0.4 (**Table S25**). Gaussian peak positions representative of carbonate minerals (~300 eV[13]) were not included in the analysis as the spectra for the mineral-coated sand did not have peaks at ~300 eV (**Figure S12, Table S25**).

*X-ray photoelectron spectroscopy (XPS) analysis*

Deconvolutions of C 1s, Fe 2p, and Mn 2p spectra were done with CasaXPS software package (**Figure S4**). The binding energy of C1s XPS located at 284.6 eV for C=C, 285 eV for C-C/C-H, 286.2 eV for C-O, 286.7 eV for C-O-C, 287.6 eV for C=O, and 289.1 eV for COO[14-16]. The binding energy of $Fe^{2+}$ $2p_{3/2}$ and $Fe^{3+}$ $2p_{3/2}$ located at 711.7 and 713.9 eV, respectively[17]. The binding energy of $Fe^{2+}$ $2p_{1/2}$ and $Fe^{3+}$ $2p_{1/2}$ located at 725.6 and 727.6 eV, respectively[17]. The Mn $2p_{3/2}$ peak was deconvoluted into 641.4–642.1 eV for $Mn^{3+}$ and 642.2–643.4 eV for $Mn^{4+}$, respectively[18].

*Synchrotron Microprobe X-ray Fluorescence (XRF) Mapping*

Hard XRF imaging and µXANES maps were done at the Stanford Synchrotron Radiation Lightsource (SSRL) on beamline 2-3. Qtz, Mn oxide- and Fe oxide-coated sand were mounted to Kapton tape. The beamline uses a Si 111 $\phi = 90$ monochromator and has a beam size of 2 x 2 µm. Elemental maps were taken for Fe, Mn, Al, Si, Ca and Mg with a 5 x 5 µm grid size. XRF spectra and correlations between elements were analyzed in Sam's MicroAnalysis Toolkit (SMAK)[19].

## Normalized Organic C Calculations

To estimate how much organic C associated with the Mn oxide and Fe oxide coating the sand, organic C from the Mn oxide- and Fe oxide-coated sand was normalized using an effective specific surface area (SSA) and SSA values from the literature. The effective SSA represents the specific surface areas of the Mn and Fe oxides coated to the sand. The area normalized organic C

concentrations using the effective SSA were compared with area normalized organic C using the literature values.

**1**

$$Normalized\ Organic\ C = \frac{\left(\frac{(C_{MO} - C_Q)}{m_{MO}}\right)}{SSA_{MO}}$$

Where $C_Q$ = Total organic C (mg $g^{-1}$) of Qtz, $C_{MO}$ = Total organic C (mg $g^{-1}$) of metal oxide (MO) treatment per g sand, $g_{MO}$ = Mass of either Mn oxide or Fe oxide (m) per g of sand and $SSA_{MO}$ = Specific surface area of either Mn oxide or Fe oxide. Prior to subtraction, the organic C associated with Qtz was averaged for each time point and depth (n = 3 replicates per treatment). The masses of the Mn oxide or Fe oxide per gram of sand were calculated using the chemical formulas $MnO_2$ for the Mn oxides and $Fe^{3+}_{10}O_{14}(OH)_2$ for the Fe oxides. Two SSA measurements were used: SSA from the literature (Mn oxide = 111.7 $m^2$ $g^{-1}$; Fe oxide = 64 $m^2$ $g^{-1}$)[20, 21] and a calculated effective SSA using the measured BET surface area for the Qtz, Mn oxide-coated sand, and Fe oxide-coated sand (**Table S4**). The effective SSA was calculated using the following formula:

$$Effective\ Specific\ Surface\ Area = \frac{SSA_{MOCS} - SSA_{Qtz}}{m_{MO}}$$

Where $SSA_{MOCS}$ was the specific surface area of the metal oxide-coated sand and $m_{MO}$ was the mass of the Mn oxide or Fe oxide prior to incubation (0.016 ± 0.001 g and 0.053 ± 0.005 g, respectively).

**Organic C Functional Groups Associated with Mn oxides and Fe oxides**

Since the organic C functional groups (OCFG) were similar for the Qtz, Mn oxide- and Fe oxide-coated sand, the OCFG associated with either the Mn oxide or Fe oxide was calculated to determine if there were differences between the mineral-coated sands. To do this, the OCFG

of the mineral-coated sands was converted from a percentage to mg $kg^{-1}$ and taking the difference between the OCFG of the Mn oxide- and Fe oxide-coated sand and the Qtz using the following set of equations:

**S2**

$$OCFG_{oxide}\left(\frac{mg}{kg}\right) = \left(\frac{Total\ C_{oxide}(\frac{mg}{kg}) * OCFG_{oxide}\ (\%)}{100}\right)$$

Where $OCFG_{oxide}$ is the OCFG associated with either the Mn oxide- or Fe oxide-coated sand and Total organic $C_{oxide}$ is the total organic C concentration of the Mn oxide- and Fe oxide-coated sand. To calculate the concentration of OCFG associated with only the Mn oxide or Fe oxide, the Qtz OCFG replicates ($OCFG_{Qtz}$; n = 2) were averaged together and the average subtracted from each replicate of the $OCFG_{oxides}$.

**S3**

$$OCFG_{sub}\left(\frac{mg}{kg}\right) = OCFG_{oxide}\left(\frac{mg}{kg}\right) - OCFG_{QTZ}(\frac{mg}{kg})$$

Where $OCFG_{sub}$ is the difference between $OCFG_{oxide}$ and Qtz OCFG ($OCFG_{Qtz}$). The $OCFG_{oxide}$ was converted into a percentage using the difference between the total organic $C_{oxide}$ the total organic C of the Qtz (Total $C_{Qtz}$):

**S4**

$$OCFG_{oxide}(\%) = \frac{OCFG_{oxide}\left(\frac{mg}{kg}\right)}{\left(Total\ C_{oxide}\left(\frac{mg}{kg}\right) - Total\ C_{Qtz}\left(\frac{mg}{kg}\right)\right)} * 100$$

The individual replicates of the Mn oxide- or Fe oxide-coated sand were averaged together and the standard error of the mean calculated.

# Supplementary Results

**WBW Soil Characteristics**

Walker Branch watershed soils were acidic, with pH decreasing with depth (4.81 ± 0.25 to 4.06 ± 0.04) (**Table S1**). Total organic C and WEOC decreased with depth, ranging from 28,894 ± 3,329 mg $kg^{-1}$ to 5,982 ± 541 mg $kg^{-1}$ for organic C and 274 ± 97 mg $L^{-1}$ to 81.6 ± 17.2 mg $L^{-1}$ for WEOC (**Table 2**). Like organic C, organic N concentrations and C/N ratios were greatest in surface soils and decreased with depth (

**Table** S2). Water-extractable Mn did not change with depth, but dithionite-extractable Mn did and ranged from 958 ± 89 mg $kg^{-1}$ 376 ± 73 mg $kg^{-1}$ (**Table S1**, **Table S3**). Dithionite-extractable Fe increased with depth from 1,235 ± 100 mg $kg^{-1}$ 2,392 ± 313 mg $kg^{-1}$ (**Table S3**); Fe was not detected in the water-extractable fraction. Water-extractable and dithionite-extractable Ca and Mg also decreased with depth (**Table S1**, **Table S3**). Silicon and P were not detected in the water-extractable or dithionite-extractable fractions. Aluminum was not detected in the dithionite-extractable fraction but was detected in the water-detectable fraction at 25 cm median core depth (**Table S1**, **Table S3**).

**References**

(1) Lafferty, B. J.; Ginder-Vogel, M.; Sparks, D. L. Arsenite oxidation by a poorly crystalline manganese-oxide 1. Stirred-flow experiments. *Environmental science & technology* **2010**, *44* (22), 8460-8466.

(2) Morgan, J. J.; Stumm, W. Colloid-chemical properties of manganese dioxide. *Journal of Colloid Science* **1964**, *19* (4), 347-359. DOI: https://doi.org/10.1016/0095-8522(64)90036-4.

(3) Brooks, S. C.; Taylor, D. L.; Jardine, P. M. Reactive transport of EDTA-complexed cobalt in the presence of ferrihydrite. *Geochimica et Cosmochimica Acta* **1996**, *60* (11), 1899-1908. DOI: https://doi.org/10.1016/0016-7037(96)00064-6.

(4) Thomas, G. W. Soil pH and soil acidity. In *Methods of Soil Analysis*, Soil Science Society of America Inc., 1996; pp 475-490.

(5) Poulton, S. W.; Canfield, D. E. Development of a sequential extraction procedure for iron: implications for iron partitioning in continentally derived particulates. *Chemical Geology* **2005**, *214* (3-4), 209-221. DOI: 10.1016/j.chemgeo.2004.09.003.

(6) Hobara, S.; Koba, K.; Ae, N.; Giblin, A. E.; Kushida, K.; Shaver, G. R. Geochemical Influences on Solubility of Soil Organic Carbon in Arctic Tundra Ecosystems. *Soil Science Society of America Journal* **2013**, *77* (2), 473-481. DOI: 10.2136/sssaj2012.0199.

(7) Paludan, C.; Jensen, H. S. Sequential extraction of phosphorus in freshwater wetland and lake sediment: significance of humic acids. *Wetlands* **1995**, *15* (4), 365-373.

(8) Ravel, B.; Newville, M. ATHENA, ARTEMIS, HEPHAESTUS: data analysis for X-ray absorption spectroscopy using IFEFFIT. *Journal of Synchrotron Radiation* **2005**, *12* (4), 537-541. DOI: doi:10.1107/S0909049505012719.

(9) Manceau, A.; Marcus, M. A.; Grangeon, S. Determination of Mn valence states in mixed-valent manganates by XANES spectroscopy. *American Mineralogist* **2012**, *97* (5-6), 816-827.

(10) Newville, M. Larch: an analysis package for XAFS and related spectroscopies. In *Journal of Physics: Conference Series*, 2013; IOP Publishing: Vol. 430, p 012007.

(11) Berens, M. J.; Michaud, A. B.; VanderJeugdt, E.; Miah, I.; Sutor, F. W.; Emerson, D.; Bowden, W. B.; Kinsman-Costello, L.; Weintraub, M. N.; Herndon, E. M. Phosphorus Interactions with Iron in Undisturbed and Disturbed Arctic Tundra Ecosystems. *Environmental Science & Technology* **2024**, *58* (26), 11400-11410. DOI: 10.1021/acs.est.3c09072.

(12) Solomon, D.; Lehmann, J.; Kinyangi, J.; Liang, B.; Schäfer, T. Carbon K-Edge NEXAFS and FTIR-ATR Spectroscopic Investigation of Organic Carbon Speciation in Soils. *Soil Science Society of America Journal* **2005**, *69*, 107-119. DOI: 10.2136/sssaj2005.0107dup.

(13) Rasmussen, M. H.; Jaye, C.; Fischer, D.; Weidner, T. A library of calcium mineral reference spectra recorded by parallel imaging using NEXAFS spectromicroscopy. *Journal of Electron Spectroscopy and Related Phenomena* **2023**, *266*, 147361. DOI: https://doi.org/10.1016/j.elspec.2023.147361.

(14) Cheng, C.-H.; Lehmann, J.; Thies, J. E.; Burton, S. D.; Engelhard, M. H. Oxidation of black carbon by biotic and abiotic processes. *Organic Geochemistry* **2006**, *37* (11), 1477-1488. DOI: https://doi.org/10.1016/j.orggeochem.2006.06.022.

(15) Huang, Y.-L.; Tien, H.-W.; Ma, C.-C. M.; Yang, S.-Y.; Wu, S.-Y.; Liu, H.-Y.; Mai, Y.-W. Effect of extended polymer chains on properties of transparent graphene nanosheets conductive film. *Journal of Materials Chemistry* **2011**, *21* (45), 18236-18241.

(16) Proctor, A.; Sherwood, P. M. X-ray photoelectron spectroscopic studies of carbon fibre surfaces. III—Industrially treated fibres and the effect of heat and exposure to oxygen. *Surface and Interface Analysis* **1982**, *4* (5), 212-219.

(17) Grosvenor, A.; Kobe, B.; Biesinger, M. C.; McIntyre, N. Investigation of multiplet splitting of Fe 2p XPS spectra and bonding in iron compounds. *Surface and Interface Analysis: An International Journal devoted to the development and application of techniques for the analysis of surfaces, interfaces and thin films* **2004**, *36* (12), 1564-1574.

(18) Santos, V.; Pereira, M.; Órfão, J.; Figueiredo, J. The role of lattice oxygen on the activity of manganese oxides towards the oxidation of volatile organic compounds. *Applied Catalysis B: Environmental* **2010**, *99* (1-2), 353-363.

(19) Webb, S. The MicroAnalysis Toolkit: X-ray fluorescence image processing software. In *AIP Conference Proceedings*, 2011; American Institute of Physics: Vol. 1365, pp 196-199.

(20) Li, H.; Bölscher, T.; Winnick, M.; Tfaily, M. M.; Cardon, Z. G.; Keiluweit, M. Simple Plant and Microbial Exudates Destabilize Mineral-Associated Organic Matter via Multiple Pathways. *Environmental Science & Technology* **2021**, *55* (5), 3389-3398. DOI: 10.1021/acs.est.0c04592.

(21) Li, H.; Reinhart, B.; Moller, S.; Herndon, E. Effects of C/Mn Ratios on the Sorption and Oxidative Degradation of Small Organic Molecules on Mn-Oxides. *Environmental Science & Technology* **2023**, *57* (1), 741-750. DOI: 10.1021/acs.est.2c03633.

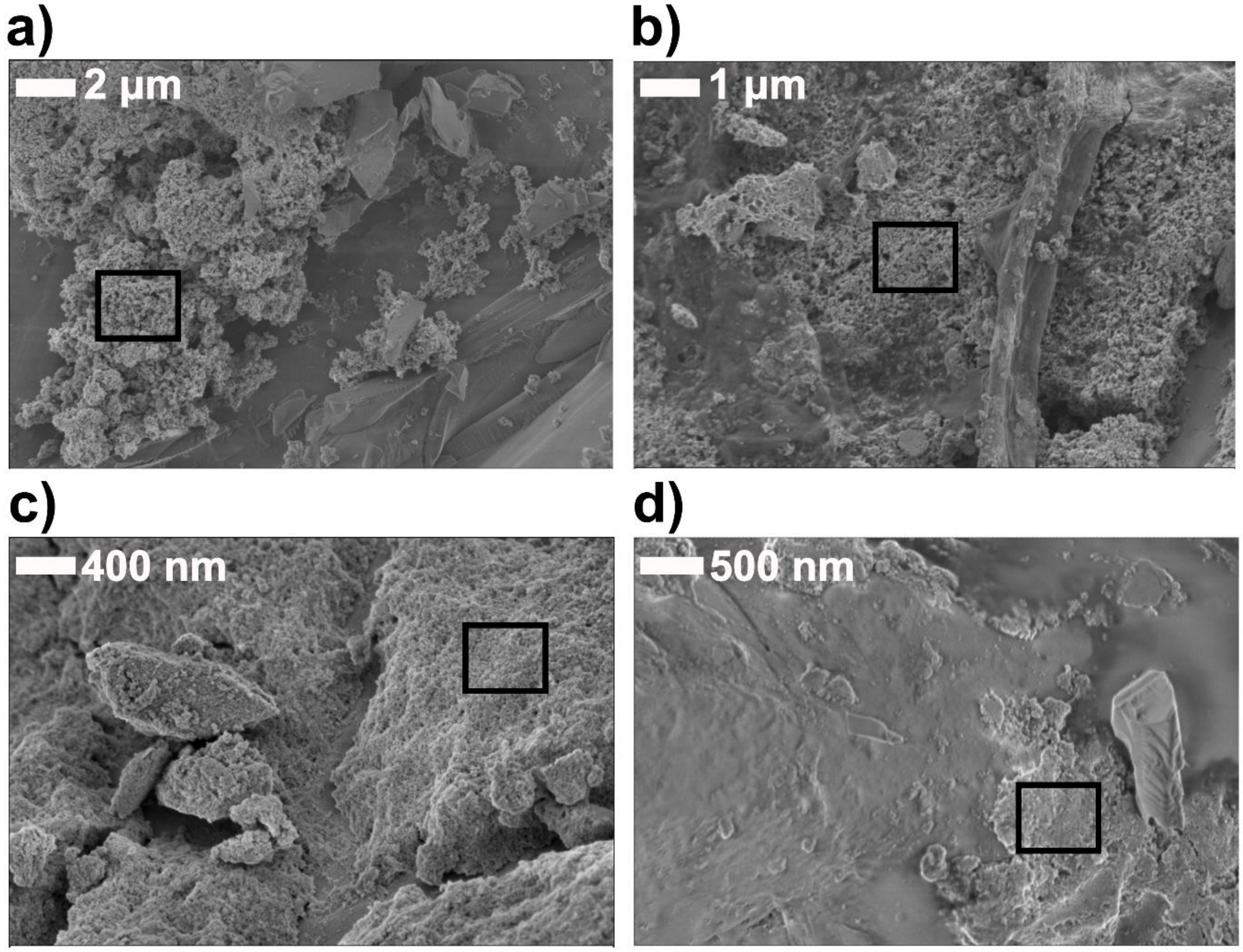


**Figure S1** The Fe oxide coating has fewer visible pores than prior to the incubation but Mn oxide-coated sand still has visible surface pores after incubation for 365 days. Panels (a, c) are the Mn oxide-coated sand (a) and Fe oxide-coated sand (c) prior to the incubation. Panels (b) and (d) are the Mn oxide-coated sand (b) and Fe oxide-coated sand (d) after incubating for 365 days. The black boxes highlight areas of oxide accumulation where pores may be before and after incubation. Micrographs were taken at 10,000x (a, b), 15,000x (c) and 20,000x (d) magnification.

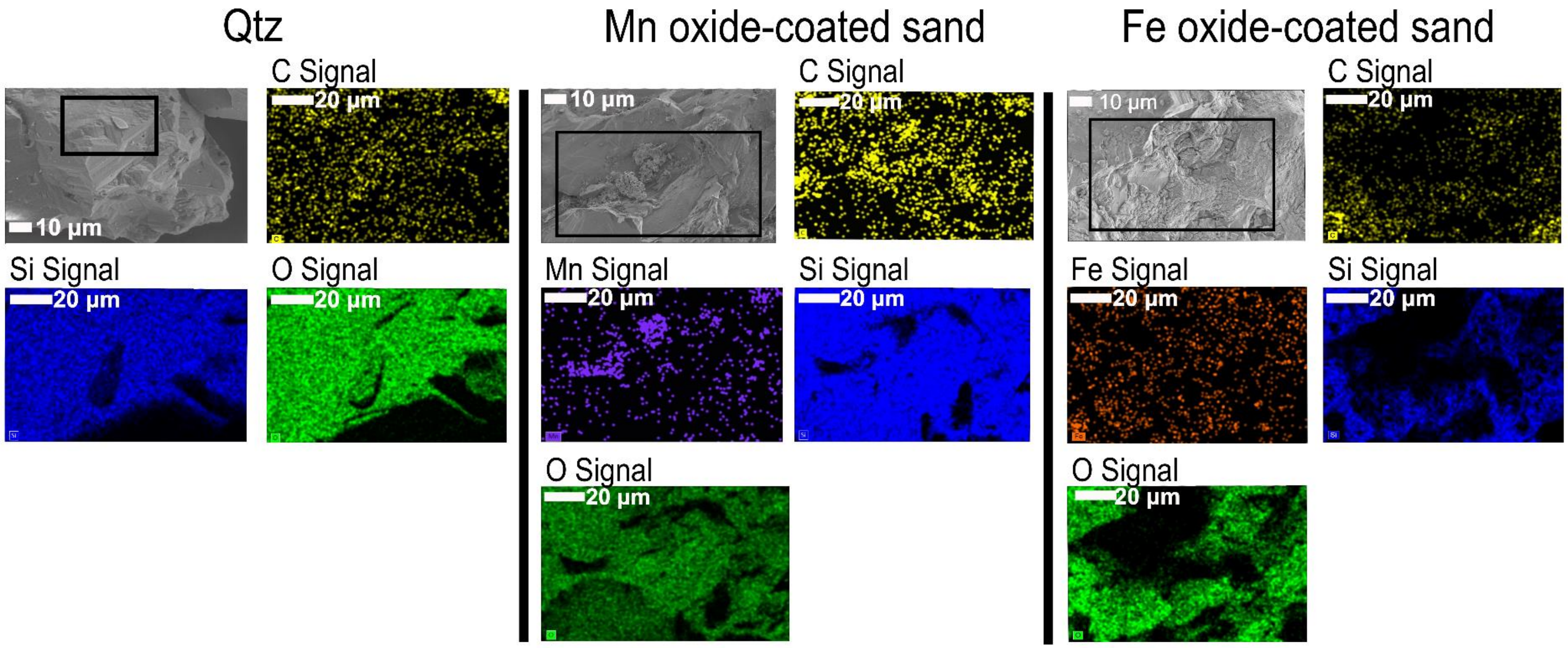


**Figure S2** The mineral-coated sand had a very low C signal prior to incubation. SEM/EDS micrographs of Qtz, Mn oxide-coated sand and Fe oxide-coated sand prior to incubation. Each sample has a micrograph taken at 1 eV at 1,000× magnification. Fe (orange), Mn (purple), C (yellow), Si (blue), Al (turquoise), O (green) and K (magenta) were mapped using EDS at 10 keV on the area outlined by the black box. The scalebar for the SEM micrographs is 10 µm and the scalebar for the EDS micrographs is 20 µm. Elements below the detection limit are not shown.

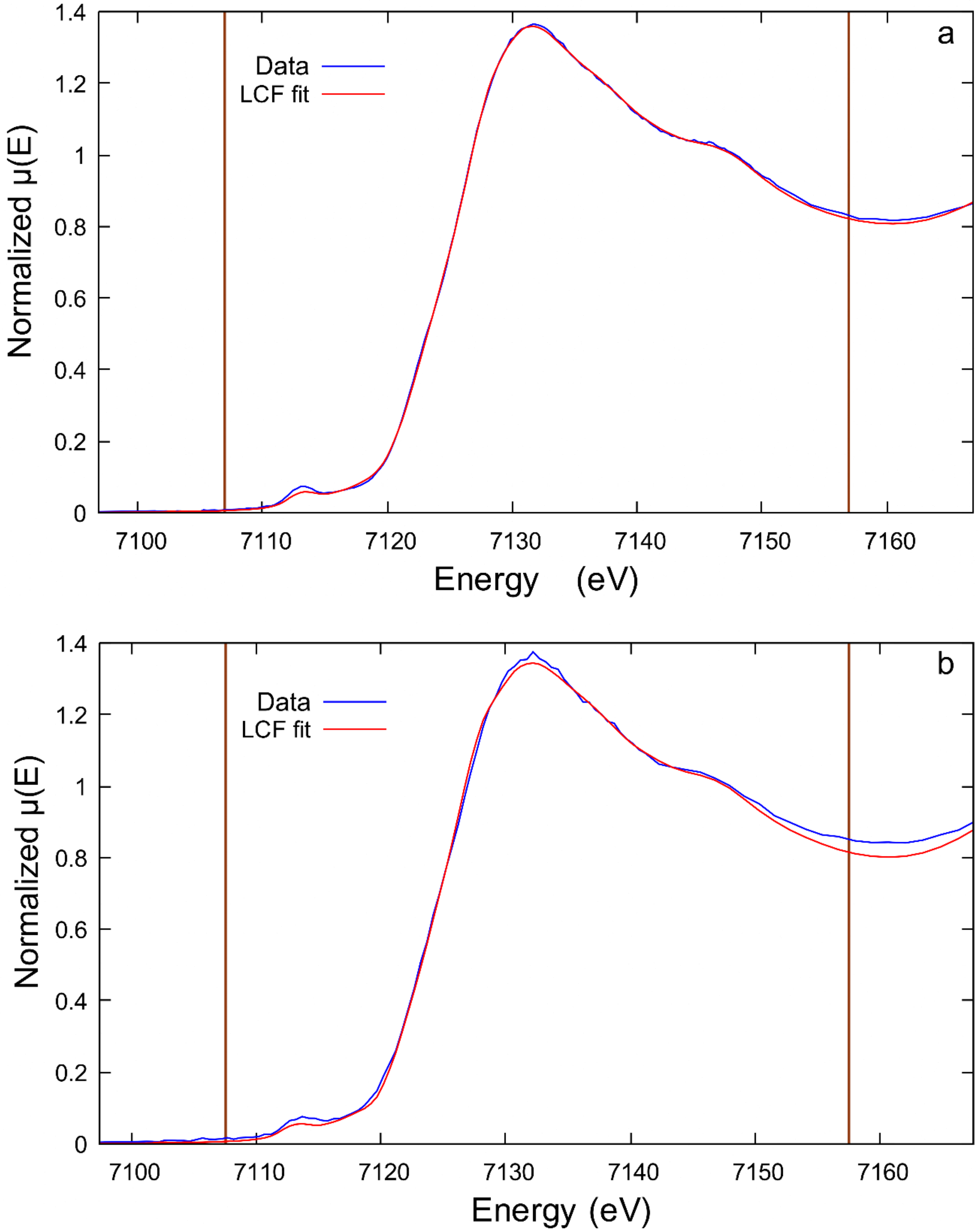


**Figure S3** Example XANES LCF fits. The fits are Fe oxide-coated sand from day 14 (a) and day 365 (b). The day 14 reduced $\chi^2$ value was 0.0000434 and the day 365 reduced $\chi^2$ value was 0.0021599. The brown lines represent -20 eV and 30 eV above and below the Fe K-edge.

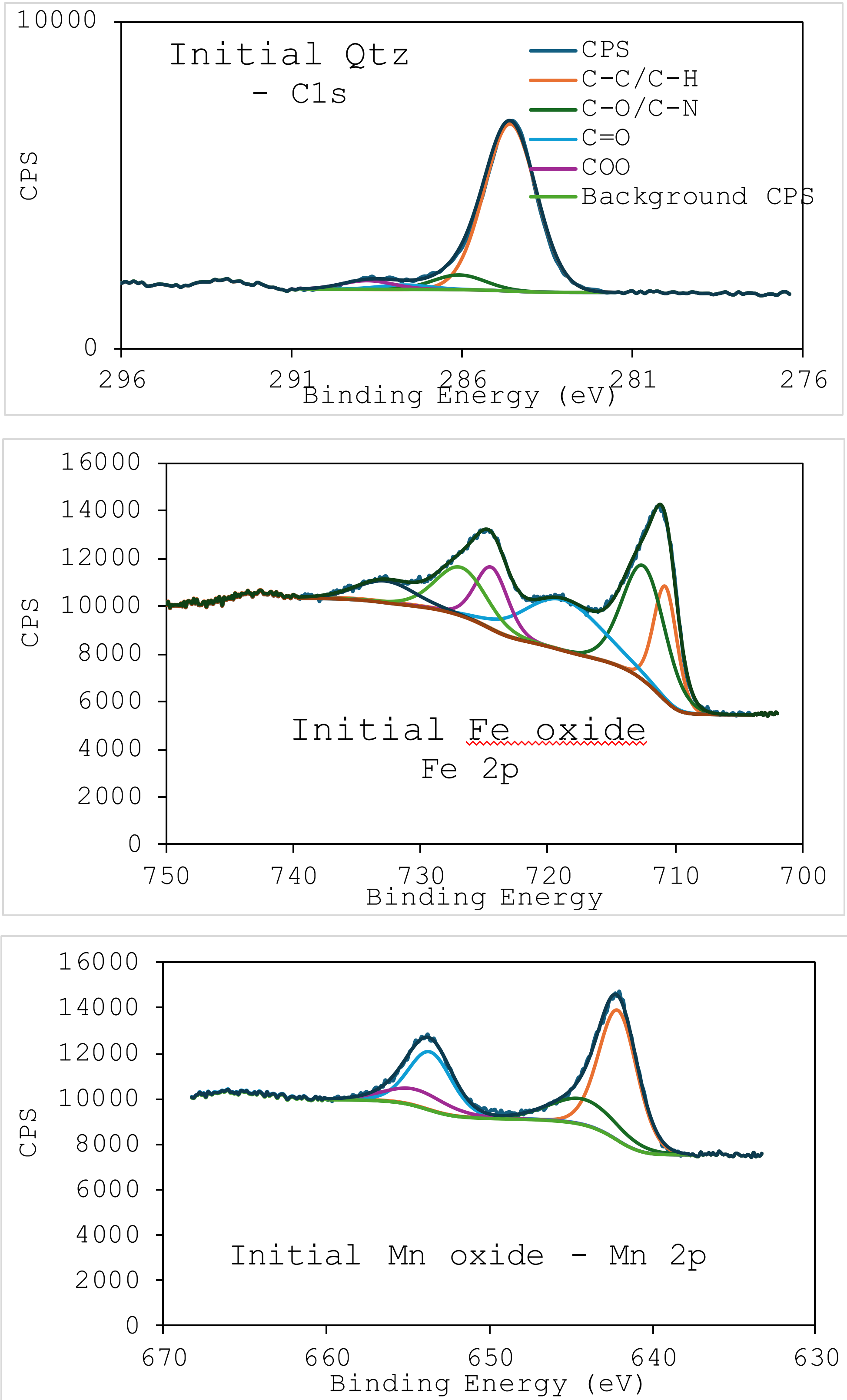


**Figure S4** Example XPS Fe, Mn and C fits.

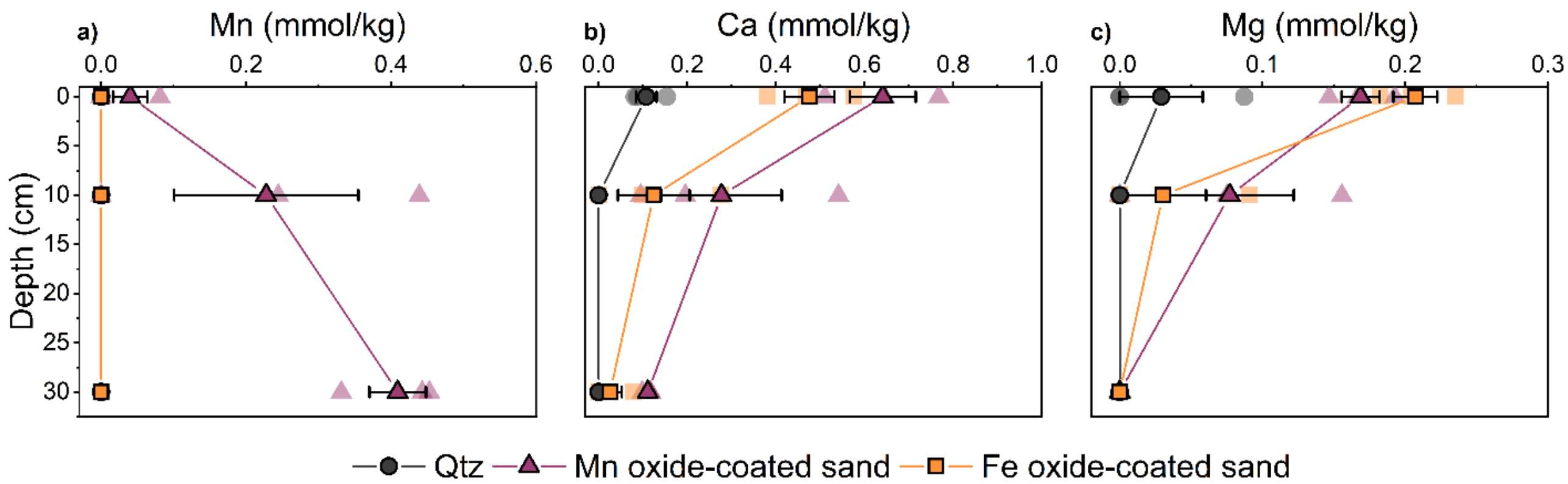


**Figure S5** Water-extractable Ca decreased with depth while water-extractable Mn increased with depth**.** Mn, Ca and Mg concentrations from the WEOC rinse for mineral bags incubated at 0, 10 and 30 cm. Ca decreased with depth for the coated and uncoated sands. Mn oxide- and Fe oxide-coated sand had some Mg associated at 0 and 10 cm while the uncoated sand had Mg only at 0 cm depth. Mn concentrations remained constant with depth for the Mn oxide-coated sand; no Mn was detected for the Fe oxide-coated sand or Qtz. Error bars represent standard error of the mean; n = 3 for all samples.

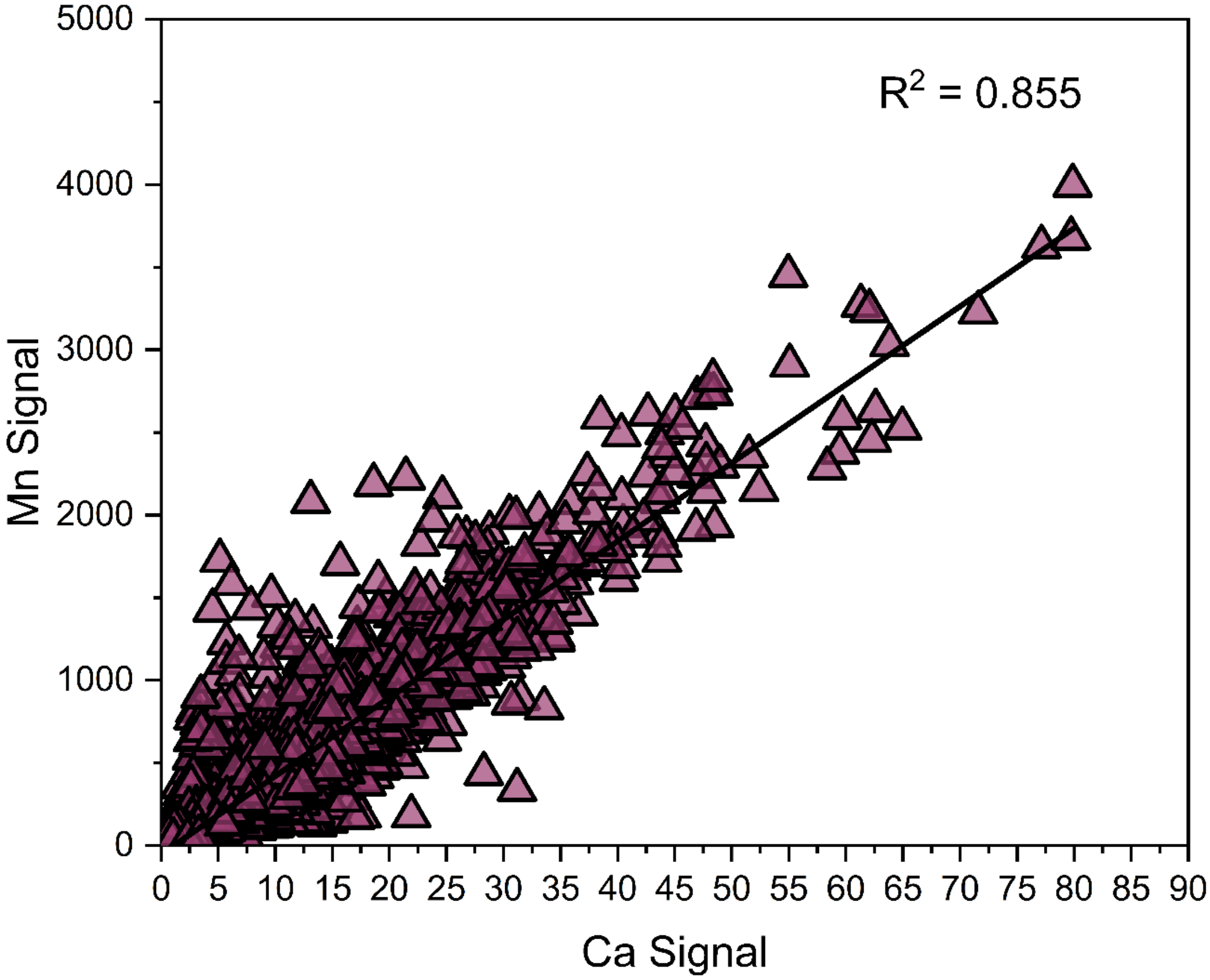


**Figure S6** Mn and Ca were strongly correlated ($R^2 = 0.855$; $F = 38{,}630$, $p<0.0001$) on the Mn oxide-coated sand incubated for 365 days.

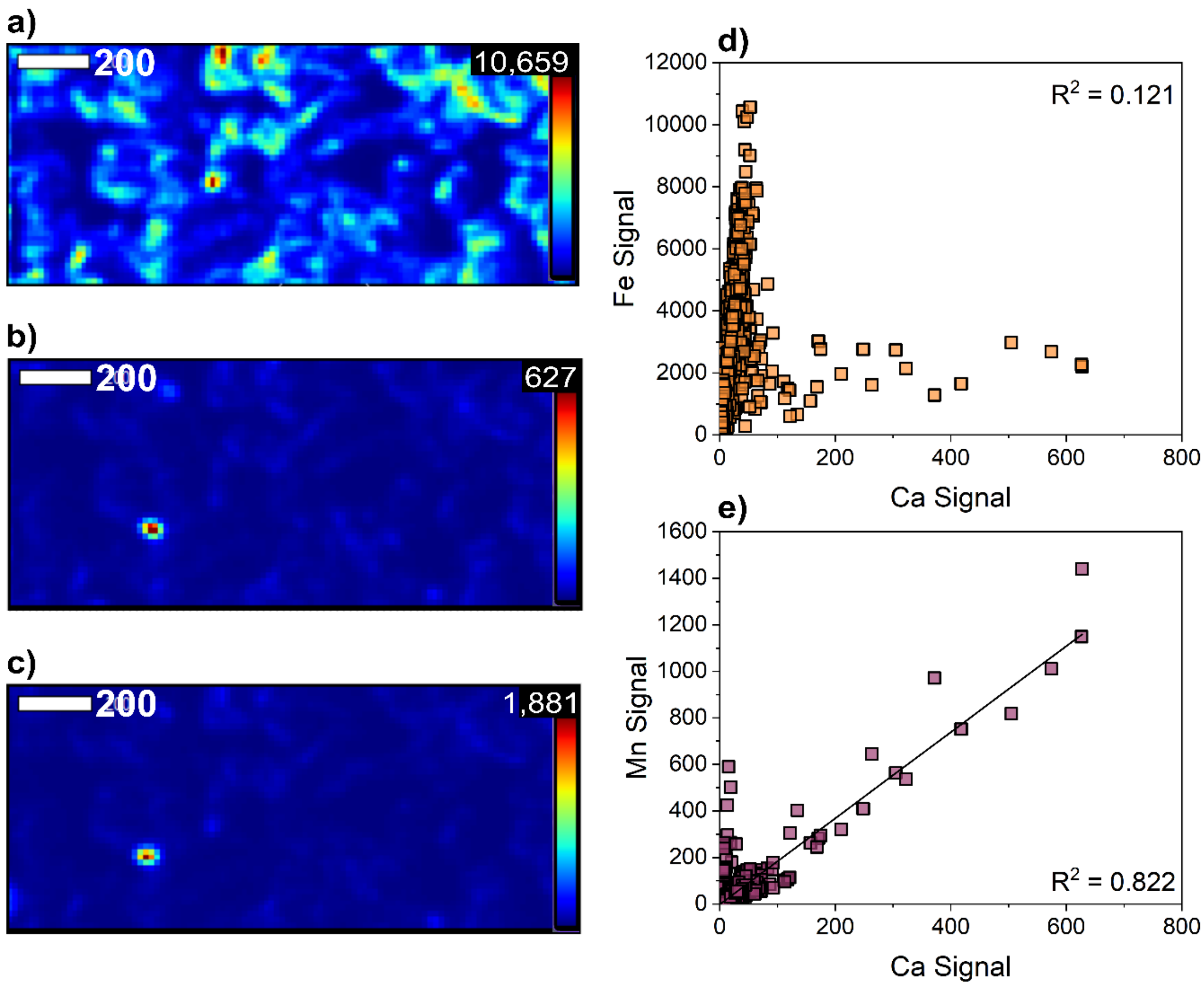


**Figure S7** XRF maps of Fe (a), Ca (b) and Mn (c) show a hotspot of Ca and Mn on the Fe oxide-coated sand incubated for 365 days. Iron and Ca were not correlated (panel d; $R^2$ = 0.121; F = 619, p<0.0001), however Mn and Ca were strongly correlated (panel e; $R^2$ = 0.822; F = 22,062, p<0.0001). Fe fluorescence intensities were 17x greater than Ca fluorescence intensities, while Mn fluorescence intensities were 2x greater than Ca fluorescence intensities.

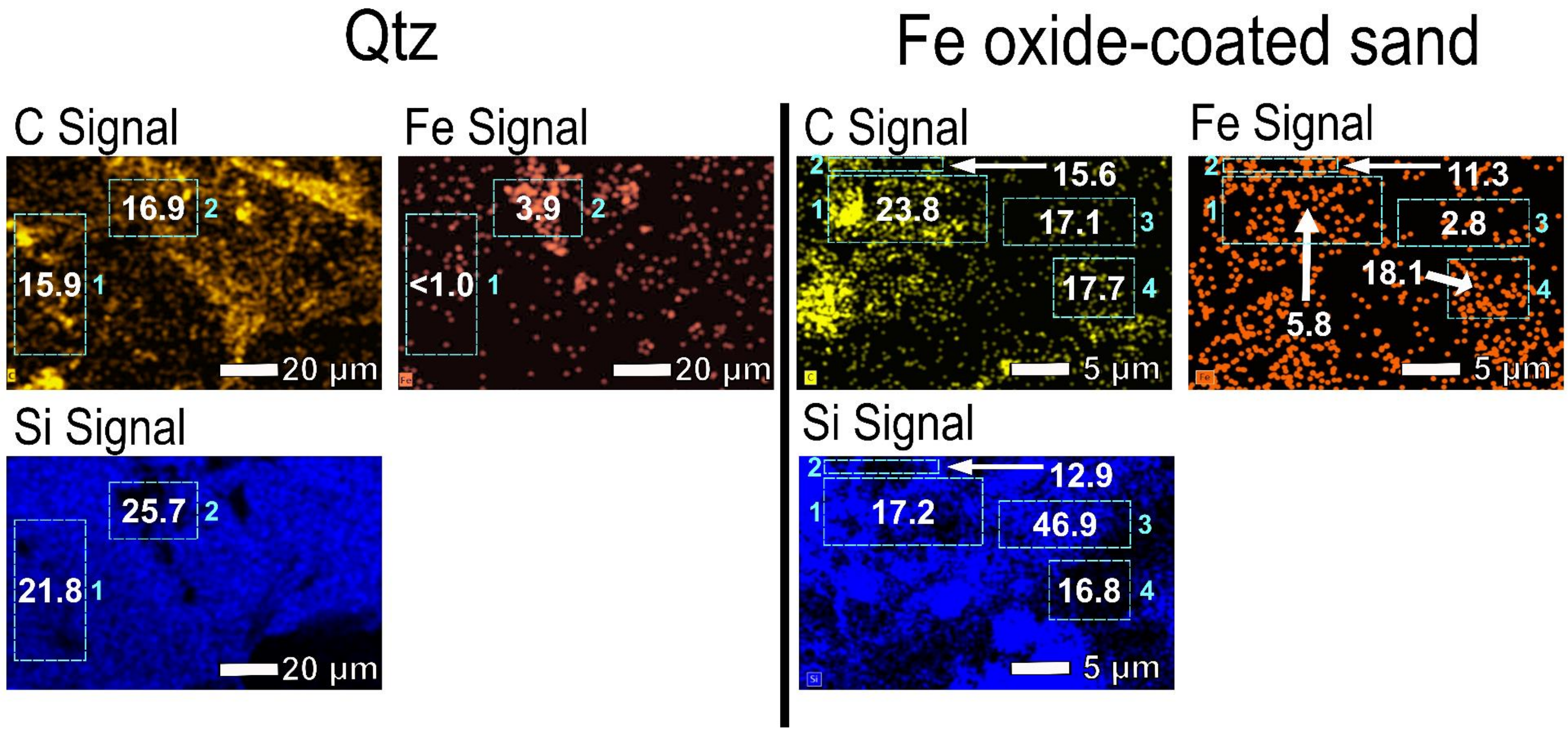


**Figure S8** Carbon associated with metal oxides on oxide-coated quartz sand. SEM/EDS micrographs of Qtz, Mn oxide-coated sand and Fe oxide-coated sand incubated for 365 days. Each sample has a micrograph taken at 10 eV at 1,000× magnification (Qtz) and 10 eV at 5,000x magnification (Fe oxide-coated sand). Fe (orange), Mn (purple), C (yellow) and Si (blue) were mapped using EDS at 10 keV on the area outlined by the black box. The scalebar for the SEM micrographs is 10 µm and the scalebar for the EDS micrographs is 20 µm. Elements below the detection limit are not shown.

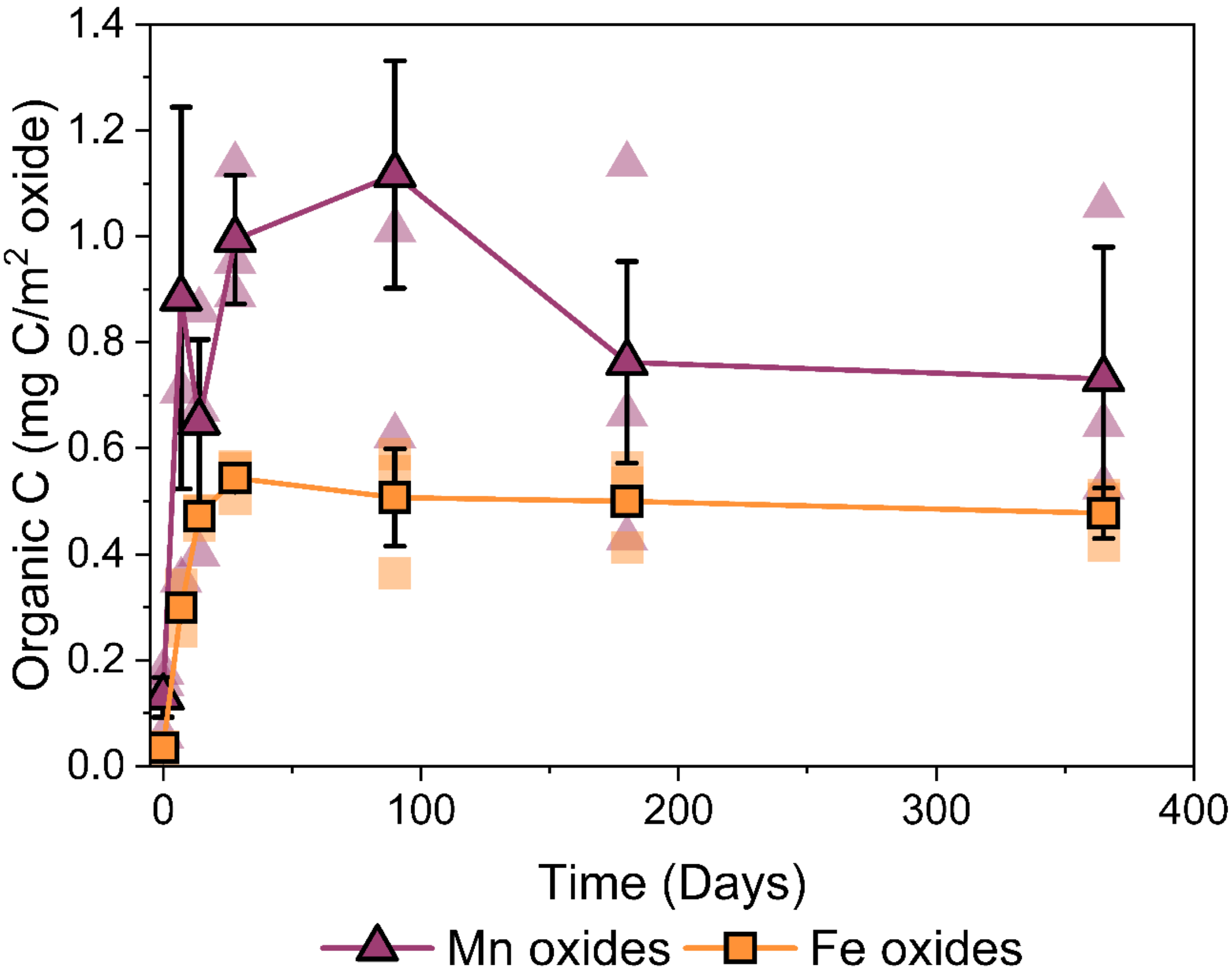


**Figure S9** Organic C normalized to the literature SSA for the Mn oxide- and Fe oxide-coated sands. The organic C changed significantly for both the Mn oxide and Fe oxide-coated sands ($F = 4.7$, $p<0.002$) and the Mn oxide-coated sand accumulated significantly more organic C than the Fe oxide-coated sand ($F = 17.4$, $p<0.0003$).The semi-transparent symbols represent field replicates. The purple triangles represent Mn oxides and the orange squares represent Fe oxides. The error bars represent the standard error of the mean.

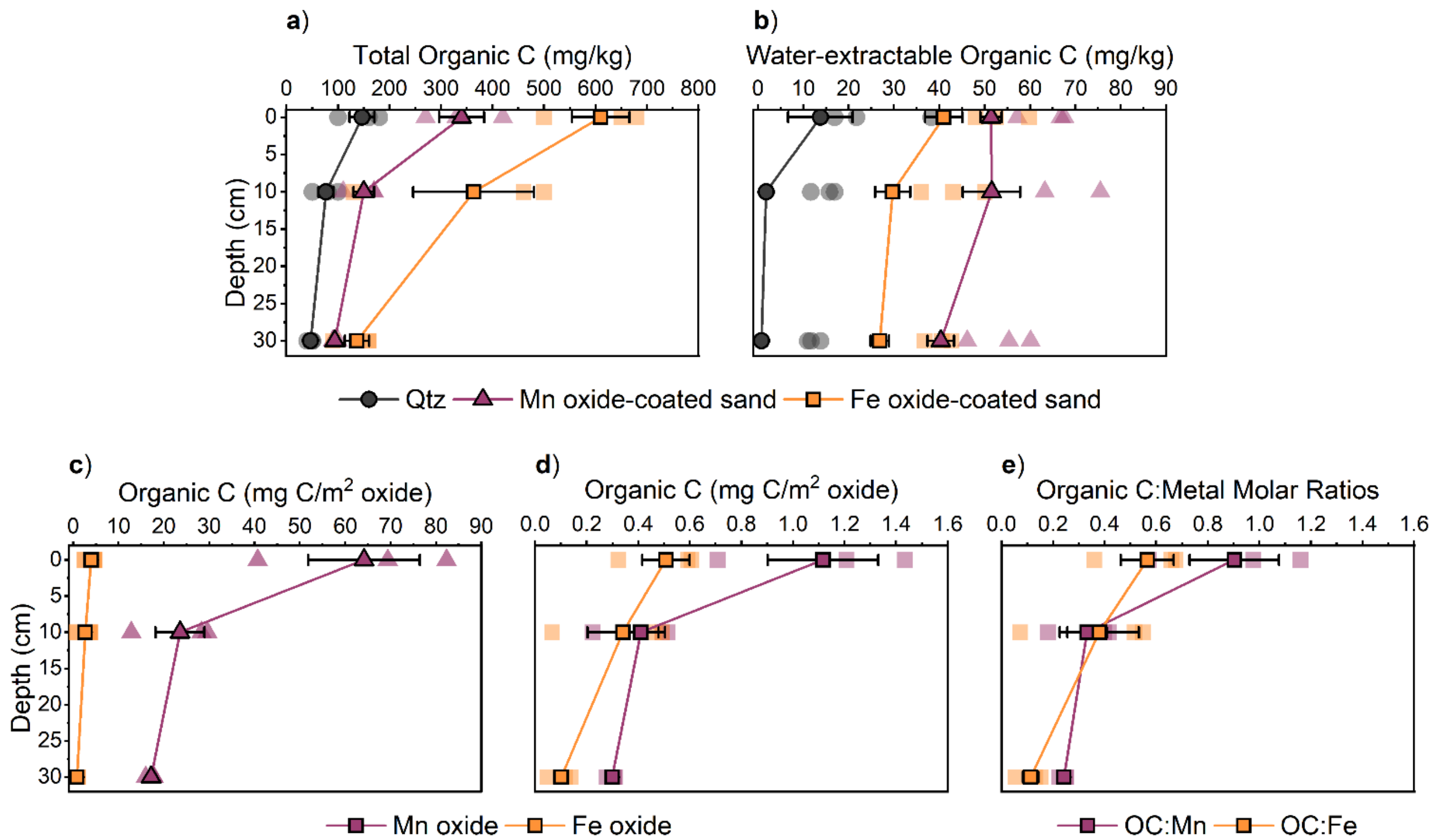


**Figure S10** Total organic C (a) ($F = 14.4$, $p<0.0007$) and WEOC ($F = 6.78$, $p<0.007$) (b) decreased with depth for all three treatments. Mn oxides accumulated more organic C with depth after normalizing organic C values using the effective SSA values ($F = 52.0$, $p<0.0001$) (c) and using the literature SSA values ($F = 6.66$, $p<0.03$) (**Table S23**) (d). Normalized organic C decreased significantly with depth when calculated using both the ESSA ($F = 11.9$, $p<0.002$) and literature values (10.1, $p<0.003$) (**Table S23**) (c, d). However, OC:molar ratios for OC:Mn and OC:Fe did not significantly decrease with depth ($F = 2.48$, $p = 0.142$) (e). Semi-transparent symbols represent replicates while filled symbols represent the average. Error bars are the standard error of three field replicates.

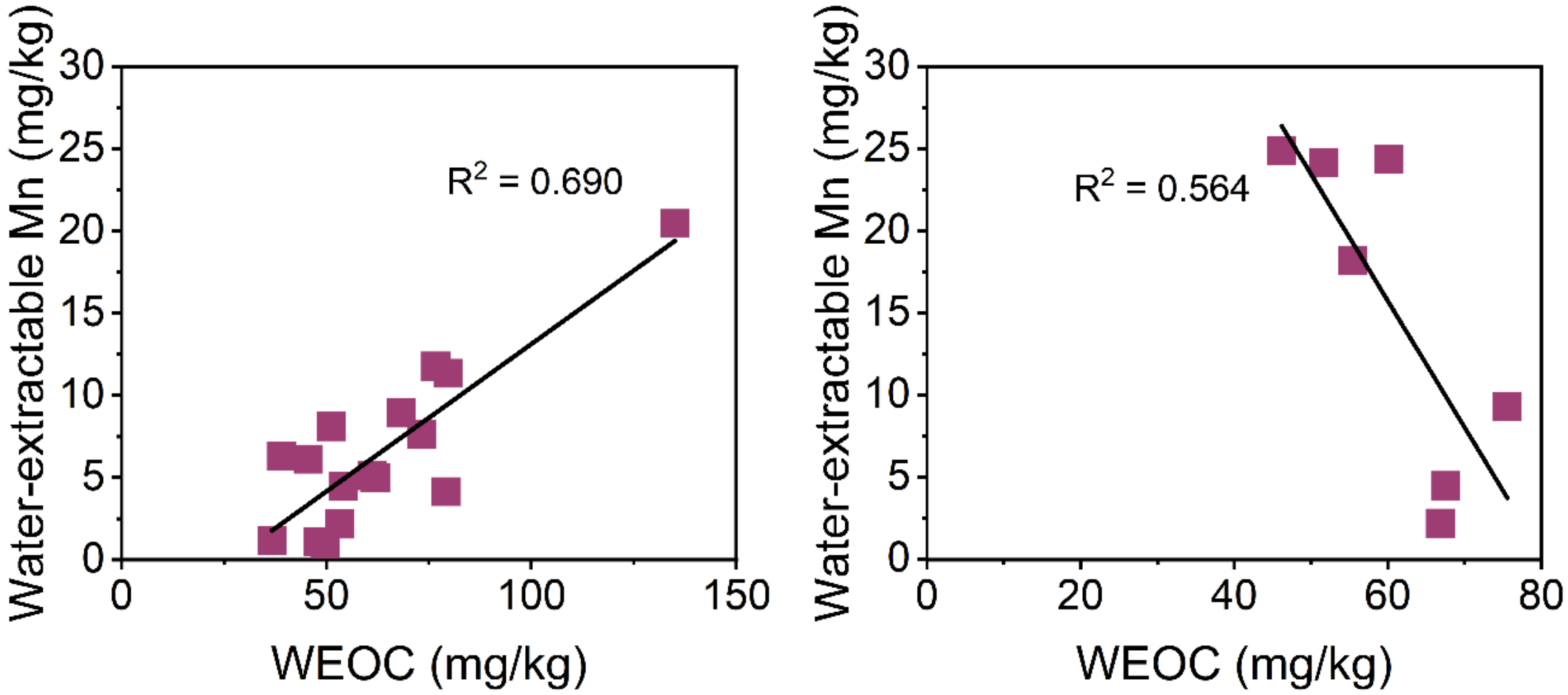


**Figure S11** Water-extractable Mn and WEOC were correlated over time (left) and with depth (right**).** WEOC and water-extractable Mn were significantly correlated over the 365 day incubation ($F = 34.4$, $p<0.0001$). Water-extractable Mn and WEOC were also significantly correlated with depth ($F = 8.77$, $p<0.05$).

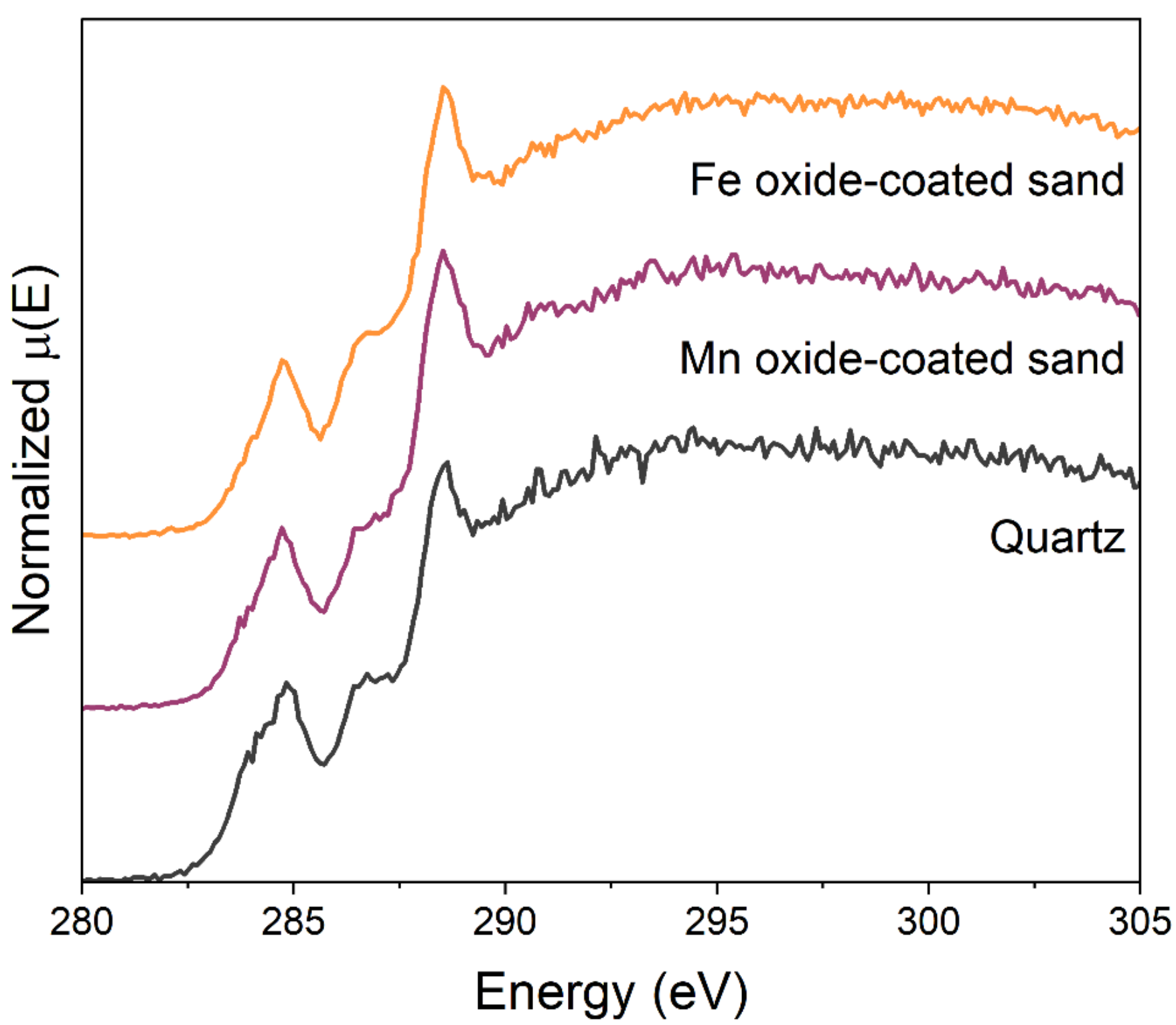


**Figure S12** Carbon K-edge NEXAFS spectra for the Qtz, Mn oxide-coated sand and the Fe oxide-coated sand. The peaks between 280 and 290 eV represent organic C functional groups. Peaks for carbonate minerals, such as calcite, would have a representative peak at ~300 eV[13].

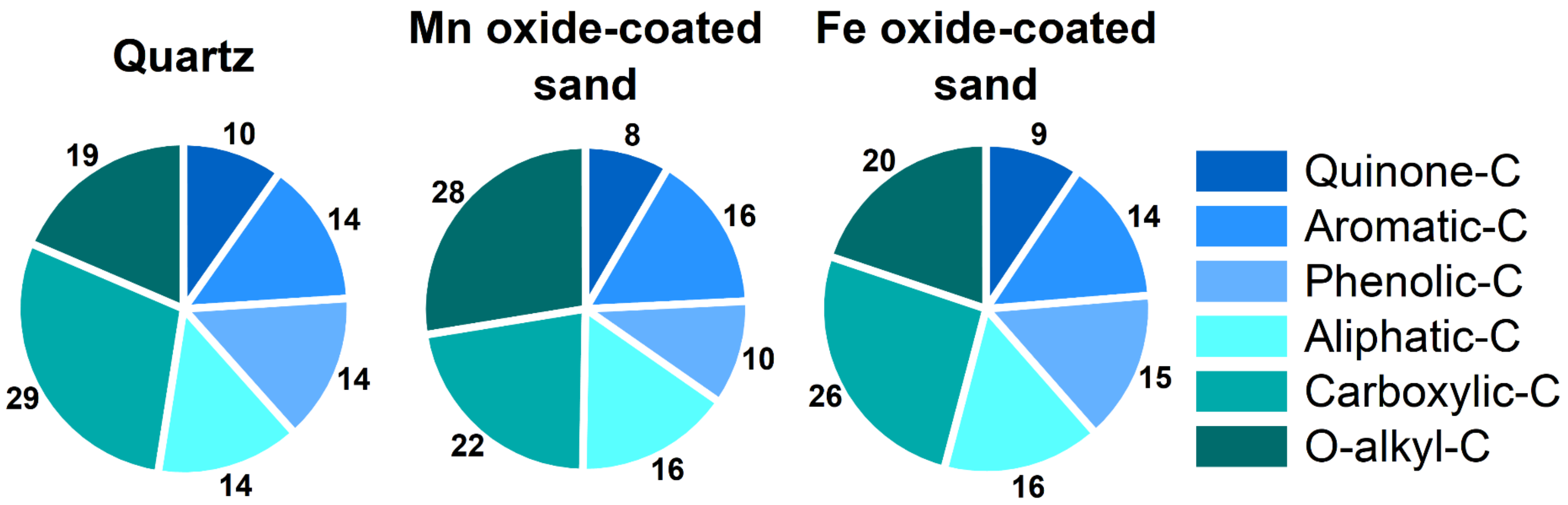


**Figure S13** Qtz, Mn oxide-coated sand and Fe oxide-coated sand had similar percentages of the organic C functional groups after 365 days measured using NEXAFS spectroscopy. Percentages with error presented in **Table S24**.

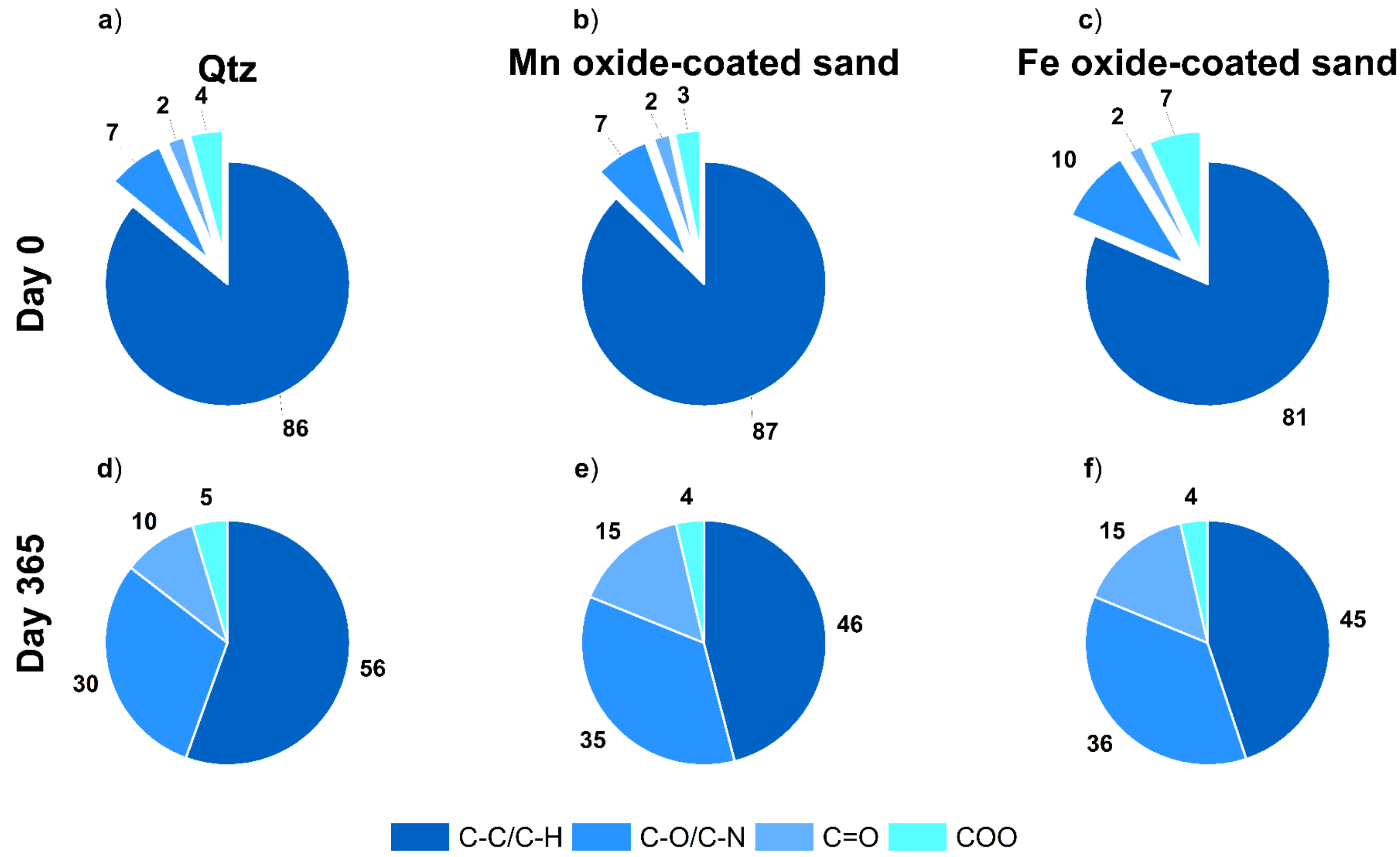


**Figure S14** The organic C functional groups for the Qtz (a, d), Mn oxide-coated sand (b, e) and Fe oxide-coated sand (c, f) for days 0 (a-c) and day 365 (d-f) measured with XPS. These organic C functional groups would be present within the top 10 nm of the mineral-coated sand.

**Table S1** Water-extractable cations extracted from soil.

| **Soil Core** | **Depth cm** | **Median Depth cm** | **pH** | **Al mg/kg** | **P mg/kg** | **Si mg/kg** | **Mn mg/kg** | **Fe mg/kg** | **Ca mg/kg** | **Mg mg/kg** |
|---|---|---|---|---|---|---|---|---|---|---|
| Soil Core 1 | 0-10 | 5 | 5.28 ± 0.099 | <10.1 | <4.42 | <10.1 | 13.2 ± 3.79 | <1.01 | 248 ± 57.3 | 67.3 ± 12.0 |
| | 10-20 | 15 | 4.23 ± 0.021 | <10.1 | <4.42 | <10.1 | 63.8 ± 4.43 | <1.01 | 65.7 ± 8.83 | 19.4 ± 1.65 |
| | 20-30 | 25 | 4.17 ± 0.015 | <10.1 | <4.42 | <10.1 | 41.5 ± 3.15 | <1.01 | 42.3 ± 5.83 | 10.9 ± 1.22 |
| Soil Core 2 | 0-10 | 5 | 4.64 ± 0.044 | <10.1 | <4.42 | <10.1 | 56.1 ± 3.25 | <1.01 | 96.5 ± 8.81 | 30.6 ± 3.12 |
| | 10-20 | 15 | 4.25 ± 0.100 | <10.1 | <4.42 | <10.1 | 54.3 ± 6.88 | <1.01 | 61.3 ± 16.4 | 22.0 ± 3.24 |
| | 20-30 | 25 | 3.94 ± 0.011 | 27.3 ± 2.02 | <4.42 | 5.14 ± 7.28 | 49.9 ± 5.73 | <1.01 | 53.4 ± 6.95 | 15.9 ± 2.24 |
| Soil Core 3 | 0-10 | 5 | 4.52 ± 0.096 | <10.1 | <4.42 | <10.1 | 31.0 ± 7.42 | <1.01 | 147 ± 66.3 | 32.9 ± 15.6 |
| | 10-20 | 15 | 4.19 ± 0.035 | <10.1 | <4.42 | <10.1 | 58.8 ± 8.38 | <1.01 | 35.0 ± 3.84 | 9.46 ± 0.770 |
| | 20-30 | 25 | 4.07 ± 0.008 | 11.7 ± 16.6 | <4.42 | <10.1 | 45.1 ± 0.209 | <1.01 | 39.6 ± 1.03 | 9.27 ± 0.023 |

**Table S2** Soil carbon, nitrogen C/N ratio and water-extractable organic C extracted from soil.

| Soil Core | Depth cm | Median Depth cm | Organic C mg/kg | Organic N mg/kg | C/N Ratio | Water-extractable Organic C mg/kg |
|---|---|---|---|---|---|---|
| Soil Core 1 | 0-10 | 5 | 39,900 ± 971 | 1,973 ± 29.1 | 20.2 ± 0.250 | 396 ± 113 |
| | 10-20 | 15 | 15,767± 433 | 927 ± 44.1 | 17.0 ± 0.356 | 198 ± 41.4 |
| | 20-30 | 25 | 5,500 ± 153 | 427 ± 6.67 | 12.9 ± 0.163 | 96.7 ± 13.7 |
| Soil Core 2 | 0-10 | 5 | 23,315 ± 1,252 | 1,324 ± 23.9 | 17.6 ± 0.911 | 235 ± 14.1 |
| | 10-20 | 15 | 10,708 ± 1,122 | 762 ± 102 | 14.2 ± 0.386 | 124 ± 5.33 |
| | 20-30 | 25 | 6,098 ± 584 | 533 ± 86.1 | 11.9 ± 1.75 | 77.4 ± 11.2 |
| Soil Core 3 | 0-10 | 5 | 23,467 ± 722 | 1,293 ± 20.3 | 18.1 ± 0.468 | 191 ± 37.7 |
| | 10-20 | 15 | 8,033 ± 145 | 547 ± 34.8 | 14.8 ± 0.668 | 79.4 ± 14.5 |
| | 20-30 | 25 | 5,767 ± 186 | 457 ± 3.33 | 12.6 ± 0.470 | 70.7 ± 4.43 |

**Table S3** Dithionite-extractable cations extracted from soil.

| Soil Core | Depth cm | Median Depth cm | Mn mg/kg | Fe mg/kg | Ca mg/kg | Mg mg/kg |
|---|---|---|---|---|---|---|
| Soil Core 1 | 0-10 | 5 | 901 ± 2.73 | 1,235 ± 13.0 | 980 ± 9.29 | 175 ± 1.67 |
| | 10-20 | 15 | 754 ± 35.8 | 1,687 ± 56.0 | 90.1 ± 11.2 | 26.5 ± 2.09 |
| | 20-30 | 25 | 295 ± 27.3 | 2,602 ± 137 | 52.0 ± 1.25 | 14.3 ± 0.981 |
| Soil Core 2 | 0-10 | 5 | 1,113 ± 35.2 | 1,317 ± 31.8 | 211 ± 15.6 | 52.1 ± 2.07 |
| | 10-20 | 15 | 467 ± 19.1 | 2,289 ± 23.7 | 55.6 ± 3.31 | 28.4 ± 1.76 |
| | 20-30 | 25 | 388 ± 20.0 | 2,662 ± 66.8 | 41.0 ± 0.844 | 17.4 ± 0.313 |
| Soil Core 3 | 0-10 | 5 | 861 ± 37.2 | 1,153 ± 44.3 | 269 ± 11.5 | 48.4 ± 2.07 |
| | 10-20 | 15 | 792 ± 8.36 | 1,494 ± 19.1 | <40.0 | 12.9 ± 0.216 |
| | 20-30 | 25 | 446 ± 15.9 | 1,912 ± 91.9 | <40.0 | 11.9 ± 0.069 |

**Table S4** Specific surface areas (SSAs) for the Qtz, Mn oxide- and Fe oxide-coated sands measured using BET analysis and SSA values from the literature

| | **Specific Surface Area** $m^2/g$ | **Specific Surface Area from the Literature** $m^2/g$ |
|---|---|---|
| Qtz | 0.137 ± 0.008 | -- |
| Mn oxide | 0.169 ± 0.006 | 111.7[21] |
| Fe oxide | 1.25 ± 0.011 | 164[20] |

**Table S5** The elements measured using SEM/EDS for the Qtz, Mn oxide- and Fe oxide-coated sand prior to incubation in atomic %. The error represents the relative uncertainty/%.

| Element | Qtz | Mn oxide-coated sand | Fe oxide-coated sand |
|---|---|---|---|
| | Atomic % | | |
| Carbon | 3.6 ± 13.7 | 6.3 ± 13.9 | 10.6 ± 3.3 |
| Oxygen | 67.8 ± 1.7 | 62.2 ± 2.4 | 59.1 ± 1.3 |
| Silicon | 28.5 ± 9.9 | 24.60 ± 11.3 | 24.0 ± 5.1 |
| Aluminum | <1.0 | <1.0 | <1.0 |
| Manganese | <1.0 | 6.9 ± 26.2 | <1.0 |
| Iron | <1.0 | <1.0 | 6.1 ± 8.8 |

**Table S6** The elements measured using SEM/EDS for the Qtz, Mn oxide- and Fe oxide-coated sand after incubating for 1 year in atomic %. The error represents the relative uncertainty/%.

| | Qtz | | Mn oxide-coated sand | | Fe oxide-coated sand | | | |
|---|---|---|---|---|---|---|---|---|
| Element | Map 1 | Map 2 | Map 1 | Map 2 | Map 1 | Map 2 | Map 3 | Map 4 |
| Carbon | 15.9 ± 4.9 | 16.9 ± 28.2 | 22.2 ± 3.7 | 5.4 ± 8.4 | 23.8 ± 5.2 | 15.6 ± 15.1 | 17.1 ± 18.1 | 17.7 ± 13.5 |
| Manganese | <1.0 | <1.0 | 13.9 ± 14.9 | 1.6 ± 23.3 | <1.0 | <1.0 | <1.0 | <1.0 |
| Iron | <1.0 | 3.9 ± 25.9 | 1.2 ± 25.4 | <1.0 | 5.8 ± 22.5 | 11.3 ± 38.7 | 2.8 ± 43.8 | 18.1 ± 33.3 |
| Silicon | 21.8 ± 8.5 | 25.7 ± 42.7 | 1.9 ± 11.1 | 25.4 ± 7.6 | 17.2 ± 9.6 | 12.9 ± 19.3 | 46.9 ± 20.7 | 16.8 ± 20.2 |
| Calcium | <1.0 | <1.0 | 2.0 ± 12.6 | <1.0 | <1.0 | <1.0 | <1.0 | <1.0 |
| Oxygen | 62.3 ± 1.7 | 52.3 ± 23.9 | 58.9 ± 1.9 | 67.6 ± 1.5 | 53.1 ± 2.3 | 39.1 ± 5.7 | 33.2 ± 6.6 | 47.4 ± 5.1 |
| Copper | <1.0 | <1.0 | <1.0 | <1.0 | <1.0 | 21.1 ± 5.0 | <1.0 | <1.0 |
| Chromium | <1.0 | 1.2 ± 25.4 | <1.0 | <1.0 | <1.0 | <1.0 | <1.0 | <1.0 |

**Table S7** Water-extractable and dithionite-extractable cations for the coated sand uncoated sand. n = 3 for all samples unless marked with an asterisk. Error is the standard error of the mean.

| | | Dithionite Extractable | | 0.01 M KCl Extractable | | | | | |
|---|---|---|---|---|---|---|---|---|---|
| **Time Days** | **Mineral** | **Mn mg/kg** | **Fe mg/kg** | **Mn mmol/kg** | **Fe mmol/kg** | **Ca mmol/kg** | **Mg mmol/kg** | **Al mmol/kg** | **Si mmol/kg** |
| 0 | Qtz | <34.3 | <51.8 | <0.114 | <0.018 | 0.269 ± 0.091 | 0.086 ± 0.061 | <0.466 | <0.360 |
| 7 | Qtz | <34.3 | <51.8 | <0.114 | <0.018 | 0.107 ± 0.017 | <0.079 | <0.466 | <0.360 |
| 14 | Qtz | <34.3 | <51.8 | <0.114 | <0.018 | 0.138 ± 0.030 | <0.079 | <0.466 | <0.360 |
| 28 | Qtz | <34.3 | <51.8 | <0.114 | <0.018 | 0.149 ± 0.028 | <0.079 | <0.466 | <0.360 |
| 90 | Qtz | <34.3 | <51.8 | <0.114 | <0.018 | 0.108 ± 0.023 | 0.029 ± 0.029 | <0.466 | <0.360 |
| 180 | Qtz | <34.3 | <51.8 | <0.114 | <0.018 | 0.269 ± 0.0392 | 0.059 ± 0.030 | <0.466 | <0.360 |
| 365 | Qtz | <34.3 | <51.8 | <0.114 | <0.018 | 0.163 ± 0.031 | <0.079 | <0.466 | <0.360 |
| 0 | Mn oxide | 1017 ± 38.7 | <39.1 | 0.125 ± 0.012 | <0.113 | 0.0346 ± 0.004 | 0.014 ± 0.014 | <0.374 | <0.360 |
| 7 | Mn oxide | 1073 ± 36.6 | <39.1 | 0.250 ± 0.063 | <0.113 | 0.414 ± 0.052 | 0.203 ± 0.064 | <0.374 | <0.360 |
| 14 | Mn oxide | 1114 ± 40.3 | <39.1 | 0.140 ± 0.038 | <0.113 | 0.477 ± 0.137 | 0.169 ± 0.033 | <0.374 | <0.360 |
| 28 | Mn oxide | 1033 ± 1.33 | <39.1 | 0.061 ± 0.031 | <0.113 | 0.810 ± 0.164 | 0.179 ± 0.002 | <0.374 | <0.360 |
| 90 | Mn oxide | 983 ± 63.4 | <39.1 | 0.040 ± 0.023 | <0.113 | 0.641 ± 0.074 | 0.169 ± 0.013 | <0.374 | <0.360 |
| 180 | Mn oxide | 981 ± 50.3 | <39.1 | <0.019 | <0.113 | 0.771 ± 0.022 | 0.186 ± 0.015 | <0.374 | <0.360 |
| 365 | Mn oxide | 959 ± 56.3 | <39.1 | 0.019 ± 0.001 | <0.113 | 0.683 ± 0.110 | 0.183 ± 0.012 | <0.374 | <0.360 |
| 0 | Fe oxide | <41.2 | 3629 ± 204 | <0.019 | <0.113 | 0.009 ± 0.009 | 0 ± 0 | <0.374 | <0.360 |
| 7 | Fe oxide | <41.2 | 3475 ± 92.3 | <0.019 | <0.113 | 0.231 ± 0.032 | 0.072 ± 0.010 | <0.374 | 0.411 ± 0.064 |
| 14 | Fe oxide | <41.2 | 3816 ± 342 | <0.019 | <0.113 | 0.316 ± 0.039 | 0.118 ± 0.027 | <0.374 | 0.431* |
| 28 | Fe oxide | <41.2 | 3606 ± 20.4 | <0.019 | <0.113 | 0.430 ± 0.014 | 0.147 ± 0.007 | <0.374 | <0.360 |
| 90 | Fe oxide | <41.2 | 3944 ± 322 | <0.019 | <0.113 | 0.475 ± 0.057 | 0.207 ± 0.015 | <0.374 | <0.360 |
| 180 | Fe oxide | <41.2 | 3842 ± 199 | <0.019 | <0.113 | 0.491 ± 0.004 | 0.192 ± 0.011 | <0.374 | <0.360 |
| 365 | Fe oxide | <41.2 | 3943 ± 37.7 | <0.019 | <0.113 | 0.465 ± 0.041 | 0.176 ± 0.003 | <0.374 | <0.360 |

*Only detected in one field replicate.

**Table S8** Water-extractable and dithionite-extractable cations for mineral bags buried at 0, 10 and 30 cm depth. Error is the standard error of three replicates. Limits of detection are in ppm units.

| | | **Dithionite-Extractable** | | **0.01 M KCl Extractable** | | | | | |
|---|---|---|---|---|---|---|---|---|---|
| **Depth cm** | **Mineral** | **Mn mg/kg** | **Fe mg/kg** | **Mn mmol/kg** | **Fe mmol/kg** | **Ca mmol/kg** | **Mg mmol/kg** | **Al mmol/kg** | **Si mmol/kg** |
| 0 | Qtz | <34.3 | <51.8 | <0.500 | <0.500 | 4.47 ± 0.919 | 1.4006* | <0.466 | <0.360 |
| 10 | Qtz | <34.3 | <51.8 | <0.500 | <0.500 | 1.21± 0.527 | <0.100 | <0.466 | <0.360 |
| 30 | Qtz | <34.3 | <51.8 | <0.500 | <0.500 | 0.789 ± 0.518 | <0.100 | <0.466 | <0.360 |
| 0 | Mn oxide | 983 ± 63.4 | <51.8 | 3.32 ± 1.14** | <0.500 | 25.7 ± 2.98 | 4.10 ± 0.324 | <0.466 | <0.360 |
| 10 | Mn oxide | 1017 ± 57.7 | <51.8 | 16.7 ± 7.41** | <0.500 | 11.3 ± 5.43 | 1.94 ± 0.893 | <0.466 | <0.360 |
| 30 | Mn oxide | 887 ± 53.0 | <51.8 | 22.5 ± 2.14 | <0.500 | 4.63 ± 0.249 | <0.100 | <0.466 | <0.360 |
| 0 | Fe oxide | <34.3 | 3944 ± 322 | <0.500 | <0.500 | 19.0 ± 2.27 | 5.03 ± 0.376 | <0.466 | <0.360 |
| 10 | Fe oxide | <34.3 | 3523 ± 40.6 | <0.500 | <0.500 | 5.34 ± 3.11 | 1.14 | <0.466 | <0.360 |
| 30 | Fe oxide | <34.3 | 3755 ± 148 | <0.500 | <0.500 | 2.46 ± 0.833 | <0.100 | <0.466 | <0.360 |

*Only detected in one field replicate.
**Only detected in two field replicates.

**Table S9** XANES and XPS values for the Mn oxide-coated sand. Error represents the standard deviation of two replicates.

| | XANES | | | | XPS | | |
|---|---|---|---|---|---|---|---|
| **Time Days** | **Mn(IV) %** | **Mn(III) %** | **Mn(II) %** | **AOS** | **Mn(IV) %** | **Mn(III) %** | **AOS** |
| 0 | 19.5* | 56.9* | 23.6* | 2.959* | 26.2* | 73.8* | 3.26 |
| 7 | 29.6 ± 3.89 | 49.6 ± 0.071 | 20.9 ± 3.54 | 3.09± 0.083 | | | |
| 14 | 29.7 ± 0.636 | 51.0 ± 1.13 | 19.4 ± 0.354 | 3.10 ± 0.001 | | | |
| 28 | 30.3 ± 6.29 | 49.8 ± 0.707 | 19.9 ± 7.07 | 3.10 ± 0.132 | | | |
| 90 | 29.3 ± 2.12 | 50.2 ± 0.990 | 20.6 ± 1.13 | 3.09 ± 0.033 | | | |
| 180 | 35.4 ± 0.071 | 49.1 ± 0.424 | 15.5 ± 0.424 | 3.20 ±0.001 | | | |
| 365 | 36.3* | 51.5* | 12.4* | 3.25* | 10.2* | 89.8* | 3.10 |

*Only one field replicate.

**Table S10** XANES and XPS values for the Fe oxide-coated sand. The error represents the standard deviation of two replicates.

| | XANES | | | XPS | | |
|---|---|---|---|---|---|---|
| **Time Days** | **Fe(III) %** | **Fe(II) %** | **AOS** | **Fe(III) %** | **Fe(II) %** | **AOS** |
| 0 | 95.0 | 5.00 | 2.95 | 65.1 | 34.9 | 2.65 |
| 7 | 90.0 ± 2.47 | 10.0 ± 2.40 | 2.90 ± 0.026 | -- | -- | -- |
| 14 | 95.0 ± 0.354 | 5.00 ± 0.424 | 2.95 ± 0.002 | -- | -- | -- |
| 28 | 92.6 ± 1.84 | 7.35 ± 1.91 | 2.93 ± 0.017 | -- | -- | -- |
| 90 | 93.5 ± 0.212 | 6.60 ± 0.141 | 2.94 ± 0.004 | -- | -- | -- |
| 180 | 92.3 ± 4.10 | 7.75 ± 4.03 | 2.92 ± 0.042 | -- | -- | -- |
| 365 | 95.3 ± 6.86 | 4.80 ± 4.80 | 2.95 ± 6.79 | 59.1 ± 0.699 | 40.9 ± 0.697 | 2.59 ± 0.007 |

**Table S11** Mn speciation and average oxidation state (AOS) for Mn oxide-coated sand buried at 0, 10 and 30 cm. The error is the standard deviation of two replicates.

| **Depth cm** | **Mn(IV) %** | **Mn(III) %** | **Mn(II) %** | **Mn AOS** |
|---|---|---|---|---|
| 0 | 29.3 ± 2.12 | 50.2 ± 0.990 | 20.6 ± 1.13 | 3.09 ± 0.033 |
| 10 | 28.4 ± 2.69 | 48.9 ± 0.495 | 22.9 ± 3.11 | 3.06 ± 0.060 |
| 30 | 23.1 ± 8.06 | 52.8 ± 4.24 | 24.2 ± 3.75 | 2.99 ± 0.120 |

**Table S12** Fe speciation and average oxidation state (AOS) for Fe oxide-coated sand buried at 0, 10 and 30 cm. The error is the standard deviation of two replicates.

| **Depth cm** | **Fe(III) %** | **Fe(II) %** | **Fe AOS** |
|---|---|---|---|
| 0 | 93.5 ± 0.212 | 6.60 ± 0.141 | 2.94 ± 0.004 |
| 10 | 95.7 ± 1.06 | 4.40 ± 0.990 | 2.96 ± 0.012 |
| 30 | 95.0 ± 1.91 | 5.05 ± 2.05 | 2.95 ± 0.016 |

**Table S13** The LCF fits for each Fe standard and the reduced $\chi^2$ values for the Fe oxide-coated sand time series. Dashed lines indicate that the standard was removed from the fit.

| | 3+ Oxidation State | | | | | 2+ Oxidation State | | | | | | |
|---|---|---|---|---|---|---|---|---|---|---|---|---|
| **Time Days** | **Goethite** | **Hematite** | **Ferrihydrite 2L** | **$FeCl_3$** | **Lepidocrocite** | **Vivianite** | **Biotite** | **Chlorite** | **FeO** | **$FeSO_4$ (s)** | **Sum** | **Reduced $\chi^2$** |
| 0 | 0 | 0 | 0.868 | 0.06 | 0.022 | 0.018 | 0.014 | -- | 0.018 | -- | 1.00 | 0.0000246 |
| 7 | 0 | 0.22 | 0.559 | 0.103 | -- | - | 0.039 | -- | 0.078 | -- | 0.999 | 0.00041 |
| 7 | 0.021 | 0.127 | 0.462 | 0.102 | 0.205 | 0.026 | 0 | -- | 0.057 | -- | 1.00 | 0.0001637 |
| 14 | 0 | 0 | 0.86 | 0.065 | 0.022 | 0.019 | 0.022 | -- | 0.012 | -- | 1.00 | 0.0000554 |
| 14 | 0 | 0 | 0.834 | 0.081 | 0.037 | 0.01 | 0.017 | -- | 0.02 | -- | 0.999 | 0.0000434 |
| 28 | 0.001 | 0.09 | 0.485 | 0.107 | 0.23 | 0.029 | 0 | -- | 0.058 | -- | 1.00 | 0.0001207 |
| 28 | 0 | 0 | 0.781 | 0.041 | 0.117 | 0.043 | 0.001 | -- | 0.016 | -- | 0.999 | 0.0000468 |
| 90 | 0 | 0.048 | 0.294 | 0.272 | 0.322 | 0 | 0 | -- | 0.056 | 0.009 | 1.00 | 0.0002624 |
| 90 | 0.001 | 0.106 | 0.342 | 0.181 | 0.303 | 0 | 0 | -- | 0.054 | 0.013 | 1.00 | 0.0001426 |
| 180 | 0 | 0.03 | 0.279 | 0.293 | 0.35 | 0 | 0 | -- | 0.024 | 0.025 | 1.00 | 0.0003358 |
| 180 | 0.1 | 0.146 | 0.228 | 0.216 | 0.204 | 0.005 | 0 | -- | 0.101 | -- | 1.00 | 0.0005489 |
| 365 | 0 | 0.015 | 0.273 | 0.28 | 0.336 | 0 | 0 | -- | 0.008 | 0.088 | 1.00 | 0.0004965 |
| 365 | 0 | 0.296 | 0.444 | 0.015 | 0.246 | -- | 0 | -- | 0 | -- | 1.00 | 0.0021599 |

**Table S14** The LCF fits for each Fe standard and the reduced $\chi^2$ values for the Fe oxide-coated sand buried at 0, 10 and 30 cm. Dashed lines indicate that the standard was removed from the fit.

| | 3+ Oxidation State | | | | | 2+ Oxidation State | | | | | |
|---|---|---|---|---|---|---|---|---|---|---|---|
| **Depth cm** | **Goethite** | **Hematite** | **Ferrihydrite 2L** | **$FeCl_3$** | **Lepidocrocite** | **Vivianite** | **Biotite** | **Chlorite** | **FeO** | **$FeSO_4$ (s)** | **Reduced $\chi^2$** |
| 0 | 0 | 0.048 | 0.294 | 0.272 | 0.322 | 0 | 0 | - | 0.056 | 0.009 | 0.0002624 |
| 0 | 0.001 | 0.106 | 0.342 | 0.181 | 0.303 | 0 | 0 | - | 0.054 | 0.013 | 0.0001426 |
| 10 | 0.123 | 0.13 | 0.222 | 0.242 | 0.247 | 0 | 0 | - | 0.012 | 0.025 | 0.0007497 |
| 10 | 0 | 0.039 | 0.273 | 0.293 | 0.344 | 0 | 0 | - | 0.025 | 0.026 | 0.0002881 |
| 30 | 0.109 | 0.12 | 0.229 | 0.249 | 0.256 | 0 | 0 | - | 0.013 | 0.023 | 0.0006255 |
| 30 | 0 | 0.045 | 0.281 | 0.289 | 0.321 | 0 | 0 | - | 0.052 | 0.013 | 0.0002654 |

**Table S15** The LCF fits for each $Mn^{4+}$ and $Mn^{3+}$ standards and the reduced $\chi^2$ values for the Mn oxide-coated sand time series. Dashed lines indicate that the standard was removed from the fit.

| | **4+ Oxidation State** | | | | **3+ Oxidation State** | | | | |
|---|---|---|---|---|---|---|---|---|---|
| **Time Days** | **Pyrosulite** | **Ramsdellite** | **$Ca_2Mn_3O_8$** | **Kbi** | **Groutite** | **Feitknechtite** | **Manganite** | **$Mn_2O_3$** | **$MnPO_4$** |
| 0 | -- | -- | 0 | 0.195 | -- | 0.569 | -- | -- | -- |
| 7 | 0.043 | 0.077 | 0.087 | 0.116 | 0.103 | 0.135 | 0.071 | 0.095 | 0.091 |
| 7 | -- | -- | 0.093 | 0.175 | 0 | 0.464 | 0 | 0 | 0.032 |
| 14 | 0.012 | 0.069 | 0.087 | 0.133 | 0.105 | 0.16 | 0.049 | 0.095 | 0.093 |
| 14 | 0.017 | 0.067 | 0.085 | 0.123 | 0.109 | 0.156 | 0.055 | 0.098 | 0.1 |
| 28 | 0.058 | 0.084 | 0.092 | 0.113 | 0.105 | 0.128 | 0.081 | 0.097 | 0.092 |
| 28 | 0.005 | 0.06 | 0.076 | 0.117 | 0.104 | 0.156 | 0.043 | 0.09 | 0.1 |
| 90 | 0.035 | 0.073 | 0.085 | 0.115 | 0.103 | 0.136 | 0.065 | 0.096 | 0.095 |
| 90 | 0.009 | 0.064 | 0.082 | 0.123 | 0.106 | 0.157 | 0.047 | 0.098 | 0.101 |
| 180 | 0.057 | 0.085 | 0.094 | 0.117 | 0.103 | 0.128 | 0.08 | 0.095 | 0.088 |
| 180 | 0.058 | 0.085 | 0.094 | 0.117 | 0.101 | 0.126 | 0.077 | 0.092 | 0.092 |
| 365 | 0.059 | 0.087 | 0.097 | 0.12 | 0.105 | 0.136 | 0.077 | 0.102 | 0.095 |

**Table S16** The LCF fits for the $Mn^{2+}$ standards and the reduced $\chi^2$ values for the Mn oxide-coated sand time series. Dashed lines indicate that the standard was removed from the fit.

| | 2+ Oxidation State | | | | | | | | | |
|---|---|---|---|---|---|---|---|---|---|---|
| **Time Days** | **Hureaulite** | **Fungi** | **Rhodochrosite** | **Manganosite** | **Pyroxmangite** | **$MnSO_4$ (aq)** | **$MnSO_4$ (s)** | **Tephroite** | **Sum** | **Reduced $\chi^2$** |
| 0 | 0.04 | -- | -- | -- | -- | 0.079 | 0.117 | -- | 1.00 | 0.0029572 |
| 7 | 0 | 0 | 0.042 | 0 | 0 | 0 | 0.142 | -- | 1.00 | 0.0008853 |
| 7 | 0.047 | 0.056 | | 0.003 | | 0.055 | 0.073 | -- | 0.998 | 0.0015748 |
| 14 | 0 | 0 | 0.03 | 0 | 0.007 | 0 | 0.159 | -- | 0.999 | 0.0013607 |
| 14 | 0 | 0 | 0.003 | 0 | 0 | 0.001 | 0.187 | -- | 1.00 | 0.0026298 |
| 28 | 0 | 0 | 0.042 | 0 | 0 | 0 | 0.107 | -- | 0.999 | 0.0010452 |
| 28 | 0 | 0 | 0.005 | 0 | 0 | 0 | 0.244 | -- | 1.00 | 0.0026112 |
| 90 | 0 | 0 | 0.028 | 0 | 0 | 0 | 0.17 | -- | 1.00 | 0.0020731 |
| 90 | 0 | 0 | 0.005 | 0 | 0 | 0 | 0.209 | -- | 1.00 | 0.0026152 |
| 180 | 0 | 0 | 0.052 | 0 | 0 | 0 | 0.1 | -- | 0.999 | 0.0010342 |
| 180 | 0 | 0 | 0.051 | 0 | 0.007 | 0 | 0.1 | -- | 1.00 | 0.0009217 |
| 365 | 0 | 0 | 0.044 | 0 | 0 | 0 | 0.08 | -- | 1.00 | 0.0021344 |

**Table S17** The LCF fits for each $Mn^{4+}$ and $Mn^{3+}$ standards and the reduced $\chi^2$ values for the Mn oxide-coated sand buried at 0, 10 and 30 cm. Dashed lines indicate that the standard was removed from the fit.

| | 4+ Oxidation State | | | | 3+ Oxidation State | | | | |
|---|---|---|---|---|---|---|---|---|---|
| **Depth cm** | **Pyrosulite** | **Ramsdellite** | **$Ca_2Mn_3O_8$** | **Kbi** | **Groutite** | **Feitknechtite** | **Manganite** | **$Mn_2O_3$** | **$MnPO_4$** |
| 0 | -0.303 | 0.345 | 0.304 | 0.107 | -0.498 | 1.004 | -0.185 | -0.064 | 0.094 |
| 0 | -0.34 | 0.507 | 0.488 | -0.072 | -0.235 | 1.227 | -0.653 | -0.181 | 0.033 |
| 10 | -0.295 | 0.62 | 0.714 | -0.313 | 0.141 | 1.122 | -0.916 | -0.296 | -0.052 |
| 10 | -0.208 | 0.301 | 0.312 | 0.032 | -0.372 | 0.881 | -0.155 | -0.066 | 0.093 |
| 30 | -0.295 | 0.634 | 0.594 | -0.313 | 0.072 | 1.597 | -1.163 | -0.336 | -0.083 |
| 30 | -0.169 | 0.4 | 0.526 | -0.194 | 0.04 | 0.961 | -0.514 | -0.201 | -0.061 |

**Table S18** The LCF fits for the $Mn^{2+}$ standards and the reduced $\chi^2$ values for the Mn oxide-coated sand buried at 0, 10 and 30. Dashed lines indicate that the standard was removed from the fit.

| | 2+ Oxidation State | | | | | | | | | |
|---|---|---|---|---|---|---|---|---|---|---|
| **Depth cm** | **Hureaulite** | **Fungi** | **Rhodochrosite** | **Manganosite** | **Pyroxmangite** | **$MnSO_4$ (aq)** | **$MnSO_4$ (s)** | **Tephroite** | **Sum** | **Reduced $\chi^2$** |
| 0 | -0.331 | 0.04 | 0.028 | 0.065 | 0.13 | -0.255 | 0.478 | 0.041 | 1 | 0.000169 |
| 0 | -0.319 | 0.049 | 0.026 | 0.078 | 0.141 | -0.353 | 0.665 | -0.062 | 0.999 | 0.000205 |
| 10 | -0.32 | 0.136 | 0.004 | 0.07 | 0.002 | -0.303 | 0.792 | -0.106 | 1 | 0.000156 |
| 10 | -0.303 | -0.046 | 0.029 | 0.003 | 0.224 | -0.264 | 0.608 | -0.07 | 0.999 | 0.000124 |
| 30 | -0.423 | 0.132 | 0.002 | 0.072 | 0.057 | -0.3 | 0.77 | -0.019 | 0.998 | 0.000288 |
| 30 | -0.285 | 0.294 | 0.007 | 0.059 | -0.099 | -0.197 | 0.474 | -0.041 | 1 | 0.000201 |

**Table S19** Total C and WEOC for Fe oxide- and Mn oxide-coated and uncoated quartz (QTZ) sand over time. Error equals standard error of the mean for three replicates except where noted.

| Time Days | Mineral | 0.01 M KCl Extractable Organic C mg/kg | Total Organic C mg/kg |
|---|---|---|---|
| 0 | Qtz | 4.60 ± 0.582 | 40.0* |
| 7 | Qtz | 9.45 ± 2.87 | 70.0 ± 5.77 |
| 14 | Qtz | 9.26 ± 2.33 | 93.3 ± 12.0 |
| 28 | Qtz | 5.78 ± 2.15 | 93.3 ± 8.82 |
| 90 | Qtz | 13.8 ± 7.11 | 147 ± 24.0 |
| 180 | Qtz | 35.6 ± 8.06 | 210 ± 32.1 |
| 365 | Qtz | 15.0 ± 0.898 | 280 ± 15.3 |
| 0 | Mn oxide | 45.2 ± 3.52 | 60.0 ± 10** |
| 7 | Mn oxide | 93.3 ± 21.0 | 230 ± 64.3 |
| 14 | Mn oxide | 77.4 ± 2.09 | 220 ± 26.5 |
| 28 | Mn oxide | 65.5 ± 3.99 | 280 ± 25.2 |
| 90 | Mn oxide | 51.4 ± 2.34 | 340 ± 43.6 |
| 180 | Mn oxide | 54.5 ± 6.80 | 343 ± 29.1 |
| 365 | Mn oxide | 44.7 ± 4.05 | 410 ± 49.3 |
| 0 | Fe oxide | 18.1 ± 0.288 | 70.0* |
| 7 | Fe oxide | 51.9 ± 2.52 | 313 ± 21.9 |
| 14 | Fe oxide | 57.6 ± 7.37 | 527 ± 47.0 |
| 28 | Fe oxide | 35.0 ± 1.04 | 563 ± 6.67 |
| 90 | Fe oxide | 41.0 ± 4.12 | 610 ± 55.7 |
| 180 | Fe oxide | 37.5 ± 3.09 | 673 ± 17.6 |
| 365 | Fe oxide | 31.8 ± 2.11 | 733 ± 41.8 |

*No replicates analyzed
**n = 2 replicates

**Table S20** Total C and WEOC for Fe oxide- and Mn oxide-coated and uncoated Qtz sand buried at 0, 10 and 30 cm depth. Error equals standard error of the mean for three replicates except where noted.

| Time Days | Mineral | 0.01 M KCl Extractable Organic C mg/kg | Total Organic C mg/kg |
|---|---|---|---|
| 0 | Qtz | 13.6 ± 7.11 | 147 ± 24.0 |
| 10 | Qtz | 1.81 ± 1.04 | 70.0 ± 5.77 |
| 30 | Qtz | 0.776 ± 0.303 | 93.3 ± 12.0 |
| 0 | Mn oxide | 51.4 ± 2.34 | 340 ± 43.6 |
| 10 | Mn oxide | 51.5 ± 6.31 | 230 ± 64.3 |
| 30 | Mn oxide | 40.3 ± 2.92 | 220 ± 26.5 |
| 0 | Fe oxide | 40.6 ± 4.12 | 610 ± 55.7 |
| 10 | Fe oxide | 29.7 ± 3.88 | 313 ± 21.9 |
| 30 | Fe oxide | 26.8 ± 2.01 | 527 ± 47.0 |

**Table S21** Concentrations of elements on the Qtz, Mn oxide- and Fe oxide-coated sand measured by XPS. Error represents the standard error of two replicates.

| Time Days | Treatment | C Wt. % | N Wt. % | O Wt. % | Mg Wt. % | Al Wt. % | Si Wt. % | Mn Wt. % | Fe Wt. % |
|---|---|---|---|---|---|---|---|---|---|
| 0 | Qtz | 15.0 ± 0.570 | 0.135 ± 0.135 | 49.7 ± 1.05 | 1.09 ± 0.145 | 1.92 ± 0.250 | 29.7 ± 0.345 | 0.035 ± 0.035 | 2.38 ± 0.835 |
| 365 | Qtz | 24.2 ± 0.745 | 1.36 ± 0.225 | 46.1 ± 0.695 | 0.545 ± 0.215 | 1.47 ± 0.48 | 25.1 ± 0.340 | 0.610 ± 0.610 | 0.545 ± 0.145 |
| 0 | Mn oxide | 9.42 ± 1.01 | 0.325 ± 0.165 | 42.1 ± 0.100 | 9.97 ± 0.230 | 0.750 ± 0.750 | 24.7 ± 0.055 | 11.8 ± 0.195 | 0.865 ± 0.235 |
| 365 | Mn oxide | 19.1 ± 2.98 | 1.46 ± 0.820 | 42.1 ± 1.64 | 7.47 ± 0.415 | 1.72 ± 0.385 | 18.9 ± 2.85 | 8.87 ± 0.415 | 0.460 ± 0.320 |
| 0 | Fe oxide | 8.74 ± 0.325 | 0 ± 0 | 47.6 ± 1.85 | 0 ± 0 | 1.65 ± 0.840 | 23.4 ± 1.52 | 0.455 ± 0.245 | 18.2 ± 2.60 |
| 365 | Fe oxide | 24.4 ± 0.916 | 1.41 ± 0.397 | 45.1 ± 0.745 | 0 ± 0 | 1.78 ± 0.404 | 15.3 ± 1.15 | 0.768 ± 0.273 | 11.4 ± 0.580 |

**Table S22** Normalized organic C values and standard error for Mn oxide- and Fe oxide-coated sand (n = 3).

| | Mn oxide-coated Sand | | | Fe oxide-coated Sand | | |
|---|---|---|---|---|---|---|
| **Time** | **Normalized Organic C Literature SSA** | **Normalized Organic C ESSA** | **OC:Mn** | **Normalized Organic C Literature SSA** | **Normalized Organic C ESSA** | **OC:Fe** |
| **Days** | **mg C/m² oxide** | **mg C/m² oxide** | **Molar Ratio** | **mg C/m² oxide** | **mg C/m² oxide** | **Molar Ratio** |
| 0 | 0.129 ± 0.037 | 7.46 ± 2.2 | 0.042 ± 0.042 | 0.0347 ± 0.002 | 0.270 ± 0.014 | 0.013 ± 0.013 |
| 7 | 0.884 ± 0.369 | 50.8 ± 20.7 | 0.715 ± 0.291 | 0.299 ± 0.0253 | 2.33 ± 0.18 | 0.333 ± 0.026 |
| 14 | 0.645 ± 0.133 | 37.3 ± 8.9 | 0.525 ± 0.126 | 0.473 ± 0.009 | 3.68 ± 0.17 | 0.527 ± 0.024 |
| 28 | 0.993 ± 0.0735 | 57.1 ± 7.0 | 0.804 ± 0.098 | 0.544 ± 0.020 | 4.23 ± 0.08 | 0.606 ± 0.011 |
| 90 | 1.10 ± 0.305 | 64.1 ± 12.3 | 0.903 ± 0.173 | 0.503 ± 0.070 | 3.95 ± 0.72 | 0.565 ± 0.103 |
| 180 | 0.743 ± 0.208 | 43.8 ± 10.9 | 0.616 ± 0.154 | 0.503 ± 0.046 | 3.89 ± 0.09 | 0.558 ± 0.012 |
| 365 | 0.743 ± 0.161 | 42.0 ± 14.3 | 0.591 ± 0.201 | 0.478 ± 0.030 | 3.72 ± 0.37 | 0.533 ± 0.053 |

**Table S23** Normalized organic C values and standard error for the depth series Mn oxide- and Fe oxide-coated sand (n =3).

| | Mn oxide-coated Sand | | | Fe oxide-coated Sand | | |
|---|---|---|---|---|---|---|
| **Depth** | **Normalized Organic C Literature SSA** | **Normalized Organic C ESSA** | **OC:Mn** | **Normalized Organic C Literature SSA** | **Normalized Organic C ESSA** | **OC:Fe** |
| **cm** | **mg C/m² oxide** | **mg C/m² oxide** | **Molar Ratio** | **mg C/m² oxide** | **mg C/m² oxide** | **Molar Ratio** |
| 0 | 1.12 ± 0.214 | 64.1 ± 12.3 | 0.903 ± 0.173 | 0.507 ± 0.092 | 3.95 ± 0.716 | 0.565 ± 0.103 |
| 10 | 0.410 ± 0.094 | 23.6 ± 5.39 | 0.332 ± 0.076 | 0.340 ± 0.138 | 2.65 ± 1.073 | 0.379 ± 0.154 |
| 30 | 0.299 ± 0.010 | 17.2 ± 0.591 | 0.242 ± 0.008 | 0.101 ± 0.027 | 0.786 ± 0.208 | 0.113 ± 0.030 |

**Table S24** Organic C functional groups for the 365 day mineral-coated sand. Error bars represent the standard error of two replicates.

| | **Quinone-C** | **Aromatic-C** | **Phenolic-C** | **Aliphatic-C** | **Carboxylic-C** | **O-alkyl-C** |
|---|---|---|---|---|---|---|
| | **%** | **%** | **%** | **%** | **%** | **%** |
| Qtz | 9.8 ± 1.0 | 14.2 ± 1.0 | 14.4 ± 0.1 | 14.1 ± 0.3 | 29.0 ± 1.0 | 18.6 ± 1.4 |
| Mn oxide | 8.4 ± 0.4 | 15.8 ± 1.6 | 10.4 ± 2.6 | 15.6 ± 0.6 | 22.2 ± 5.3 | 27.6 ± 6.1 |
| Fe oxide | 9.4 ± 1.7 | 14.3 ± 0.3 | 14.8 ± 0.2 | 15.6 ± 0.4 | 26.1 ± 1.4 | 19.9 ± 2.6 |

**Table S25** Organic C functional group peak areas and reduced $\chi^2$ for the Qtz, Mn oxide- and Fe oxide-coated sands measured using NEXAFS.

| **Time Days** | **Mineral** | **Reduced $\chi^2$** | **Quinone-C** | **Aromatic-C** | **Phenolic-C** | **Aliphatic-C** | **Carboxylic-C** | **O-alkyl-C** |
|---|---|---|---|---|---|---|---|---|
| 365 | Qtz | 0.0006827 | 0.373 | 0.522 | 0.499 | 0.495 | 0.964 | 0.59 |
| 365 | Qtz | 0.0005421 | 0.269 | 0.407 | 0.439 | 0.422 | 0.919 | 0.615 |
| 365 | Mn oxide | 0.000577 | 0.295[1] | 0.472 | 0.431 | 0.498 | 0.913 | 0.713 |
| 365 | Mn oxide | 0.0002371 | 0.211 | 0.458 | 0.207 | 0.426 | 0.444 | 0.886 |
| 365 | Fe oxide | 0.0004346 | 0.231 | 0.435 | 0.436 | 0.478 | 0.736 | 0.671 |
| 365 | Fe oxide | 0.0009325 | 0.43 | 0.544 | 0.581 | 0.587 | 1.065 | 0.669 |

**Table S26** Organic C functional groups for the Mn oxide- and Fe oxide-coated sand after the Qtz organic C functional groups were subtracted (see **Supplementary Methods**). Error represents the standard error between two replicates.

| **Mineral-coated Sand** | **Quinone-C** | **Aromatic-C** | **Phenolic-C** | **Aliphatic-C** | **Carboxylic-C** | **O-alkyl-C** |
|---|---|---|---|---|---|---|
| | % | % | % | % | % | % |
| Mn oxide | 5.29 ± 1.59 | 17.71 ± 6.04 | 3.48 ± 5.46 | 18.3 ± 1.65 | 11.5 ± 14.6 | 43.7 ± 10.8 |
| Fe oxide | 9.19 ± 3.38 | 14.4 ± 0.168 | 15.0 ± 0.383 | 16.5 ± 0.493 | 24.2 ± 1.69 | 20.6 ± 5.13 |

**Table S27** The percentages of each organic C functional group on the surface (top 10 nm) of the Qtz, Mn oxide- and Fe oxide-coated sands prior to incubation (day 0) and after incubation for 365 days measured using XPS. Each column represents an organic C functional group, with the name of that group and its respective energy.

| Time | Oxide-coated Sand | C-C/C-H | C-O/C-N | C=O | COO |
|---|---|---|---|---|---|
| | | 284.6 eV | 286.1 eV | 287.7 eV | 288.8 eV |
| | | Aromatic | Alcohol/ polysaccharide/amine | Carbonyl | Ester/carboxylic |
| Days | | % | | | |
| 0 | Qtz | 86.0 | 7.4 | 2.2 | 4.4 |
| | Mn oxide | 87.4 | 7.1 | 2.1 | 3.4 |
| | Fe oxide | 81.5 | 9.8 | 1.8 | 6.9 |
| 365 | Qtz | 55.5 | 30.0 | 9.9 | 4.6 |
| | Mn oxide | 45.9 | 35.2 | 15.2 | 3.6 |
| | Fe oxide | 44.0 | 37.5 | 15.5 | 2.9 |
| | Fe oxide | 45.7 | 35.1 | 15.0 | 4.2 |

**Table S28** Organic C functional groups for leaf litter collected at WBW.

| Sample ID | Quinone-C % | Aromatic-C % | Phenolic-C % | Aliphatic-C % | Carboxylic-C % | O-alkyl-C % |
|---|---|---|---|---|---|---|
| C2Mn0T3 | 16.5 | 27.9 | 14.5 | 13.2 | 19.3 | 8.56 |
| C3Mn0T3 | 16.7 | 27.9 | 16.3 | 13.5 | 18.5 | 7.06 |
| C4Mn0T1 | 13.4 | 25.3 | 17.5 | 13.4 | 22 | 8.44 |
| C4Mn0T3 | 16.2 | 29.5 | 16.5 | 13.1 | 17.3 | 7.44 |
| **Average** | 15.4 ± 0.687 | 27.1 ± 0.888 | 16.2 ± 0.481 | 13.4 ± 0.382 | 19.8 ± 0.96 | 7.82 ± 0.292 |

**Table S29** The scattering intensity for the Qtz, Mn oxide- and Fe oxide-coated sand before incubation and after incubating for 365 days. The intensities are from the scattering vector Q 0.32 Å$^{-1}$, which is in the middle of the high Q range (0.1-0.6 Å$^{-1}$).

| | **Initial Material** | **Incubated for 365** |
|---|---|---|
| | **Scattering Intensity** | |
| | **cm$^{-1}$** | **cm$^{-1}$** |
| **Qtz** | 2.62 x $10^{-4}$ ± 8.22 x $10^{-5}$ | 5.77 x $10^{-3}$ ± 9.75 x $1^{0-5}$ |
| **Mn oxide-coated sand** | 4.43 x $10^{-4}$ ± 7.35 x $10^{-5}$ | 4.05 x $10^{-3}$ ± 1.04 x $10^{-4}$ |
| **Fe oxide-coated sand** | 3.98 x $10^{-3}$ ± 1.49 x $10^{-4}$ | 8.01 x $10^{-3}$ ± 1.54 x $10^{-4}$ |
